# Amino Acids as Chelating Ligands for Platinum: Enhanced Stability in an Aqueous Environment Promoted by Biocompatible Molecules

**DOI:** 10.1021/acs.jmedchem.3c01340

**Published:** 2023-11-08

**Authors:** Andrea Cucchiaro, Amelie Scherfler, Davide Corinti, Giel Berden, Jos Oomens, Klaus Wurst, Ronald Gust, Maria Elisa Crestoni, Brigitte Kircher, Monika Cziferszky

**Affiliations:** † Institute of Pharmacy, Pharmaceutical Chemistry, Center for Molecular Biosciences Innsbruck, University of Innsbruck, Innrain 80-82, A-6020 Innsbruck, Austria; ‡ Dipartimento di Chimica e Tecnologie del Farmaco, Università di Roma “La Sapienza”, P. le A. Moro 5, I-00185 Roma, Italy; § Institute for Molecules and Materials, FELIX Laboratory, Radboud University, Toernooiveld 7, 6525ED Nijmegen, The Netherlands; ∥ Institute of General, Inorganic and Theoretical Chemistry, CCB-Centrum for Chemistry and Biomedicine, University of Innsbruck, Innrain 80-82, 6020 Innsbruck, Austria; ⊥ Tyrolean Cancer Research Institute, Innrain 66, 6020 Innsbruck, Austria; # Immunobiology and Stem Cell Laboratory, Department of Internal Medicine V (Hematology and Oncology), Medical University of Innsbruck, Anichstraße 35, 6020 Innsbruck, Austria

## Abstract

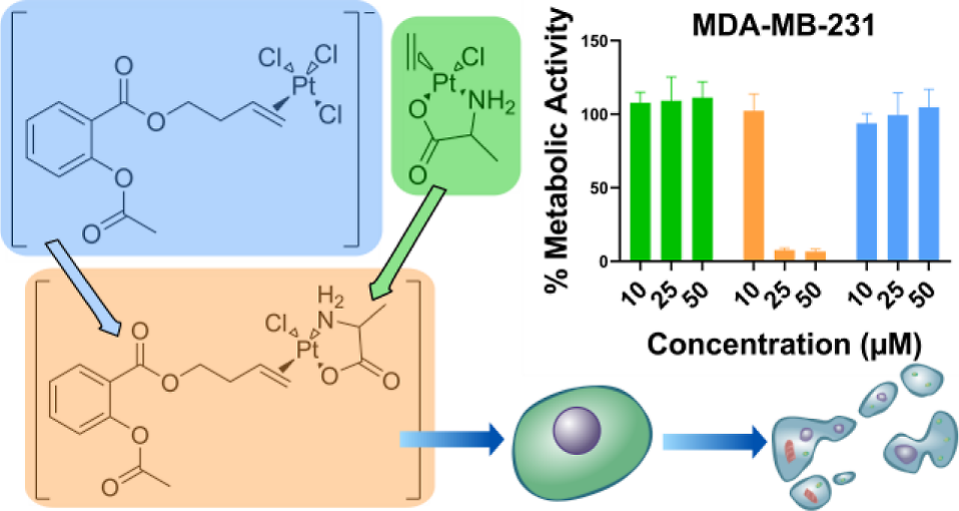

Platinum-based chemotherapeutics are a cornerstone in the treatment of many malignancies. However, their dose-limiting side effects have rooted efforts to develop new drug candidates with higher selectivity for tumor tissues and less problematic side effects. Here, we developed a cytotoxic platinum(II) complex based on Zeise’s salt, containing the nonsteroidal anti-inflammatory drug acetylsalicylic acid and alanine as ligands (**4**). The previously developed complex (**5**) displayed high reactivity against sulfur-containing biomolecules; therefore, we put the focus on the optimization of the structure regarding its stability. Different amino acids were used as biocompatible chelating ligands to achieve this aim. Differences in the coordination sphere caused pronounced changes in the stability of Zeise-type precursors **1–3**. Coordination with l-Ala through N in the *trans* position to ethylene showed the most promising results and was employed to stabilize **5**. As a result, complex **4** showed improved stability and cytotoxicity, outperforming both **5** and **1**.

## Introduction

Cisplatin is the first and most widely used platinum-based anticancer drug for chemotherapy in standard healthcare.^1,2^ However, the use of this drug is limited by severe side effects, for example, nephrotoxicity, hepatotoxicity, cardiotoxicity, nausea, vomiting, and ototoxicity.^2,3^ Furthermore, various cancers show an intrinsic or acquired resistance to cisplatin and, due to the similar mechanism of action, exhibit cross-resistance against other platinum-based antitumor agents as well, that is, oxaliplatin and carboplatin.^4^ For these reasons, the motivation to improve the clinical performance of platinum-based chemotherapeutics leads to continuous research efforts to develop new drugs with higher efficacy, fewer side effects, and the capability to overcome intrinsic and acquired resistance.

A reasonable strategy to overcome the aforementioned drawbacks is to consider a different therapeutic target, which also provides higher selectivity for tumor cells. Particularly interesting candidates are cyclooxygenases (COXs) and especially COX-2.^5^ This is an inducible enzyme not detected in most tissues (except for the kidney, seminal vessels, and central nervous system) and is usually related to an inflammatory response.^5^ COXs are responsible for the biosynthesis of prostanoids, the most abundant of which is PGE_2_.^6^

COX-2 is overexpressed in several tumors, including colorectal, breast, stomach, lung, and pancreatic cancer.^7^ Moreover, there are suggestions that higher levels of COX-2 may be related to a bad prognosis for patients and that increased prostaglandin (PG) levels in cancer cells can also be caused by chemotherapy and radiotherapy.^8^ Several studies highlight how COX-2 is likely involved in carcinogenesis and cancer progression, affecting aspects like xenobiotic metabolism, angiogenesis, inhibition of apoptosis, immunosuppression, and invasiveness.^9^ Also, there is evidence for PGE_2_ to contribute to angiogenesis, tumor promotion, and cellular apoptosis resistance.^10^ The use of COX-2 selective inhibitors is often addressed as a powerful tool in the fight against cancer.^11,12^ Moreover, several preclinical studies suggest the possibility for COX-2 selective inhibitors to enhance the effects of chemotherapy and radiotherapy.^8^ All these studies support the hypothesis that COX-2 selective inhibitors may be an interesting class of compounds as chemo-preventive, cytostatic, or cytotoxic agents, given the well-known low side effect profile compared to classic and nonselective nonsteroidal anti-inflammatory drugs (NSAIDs).^13^

**Scheme 1:**
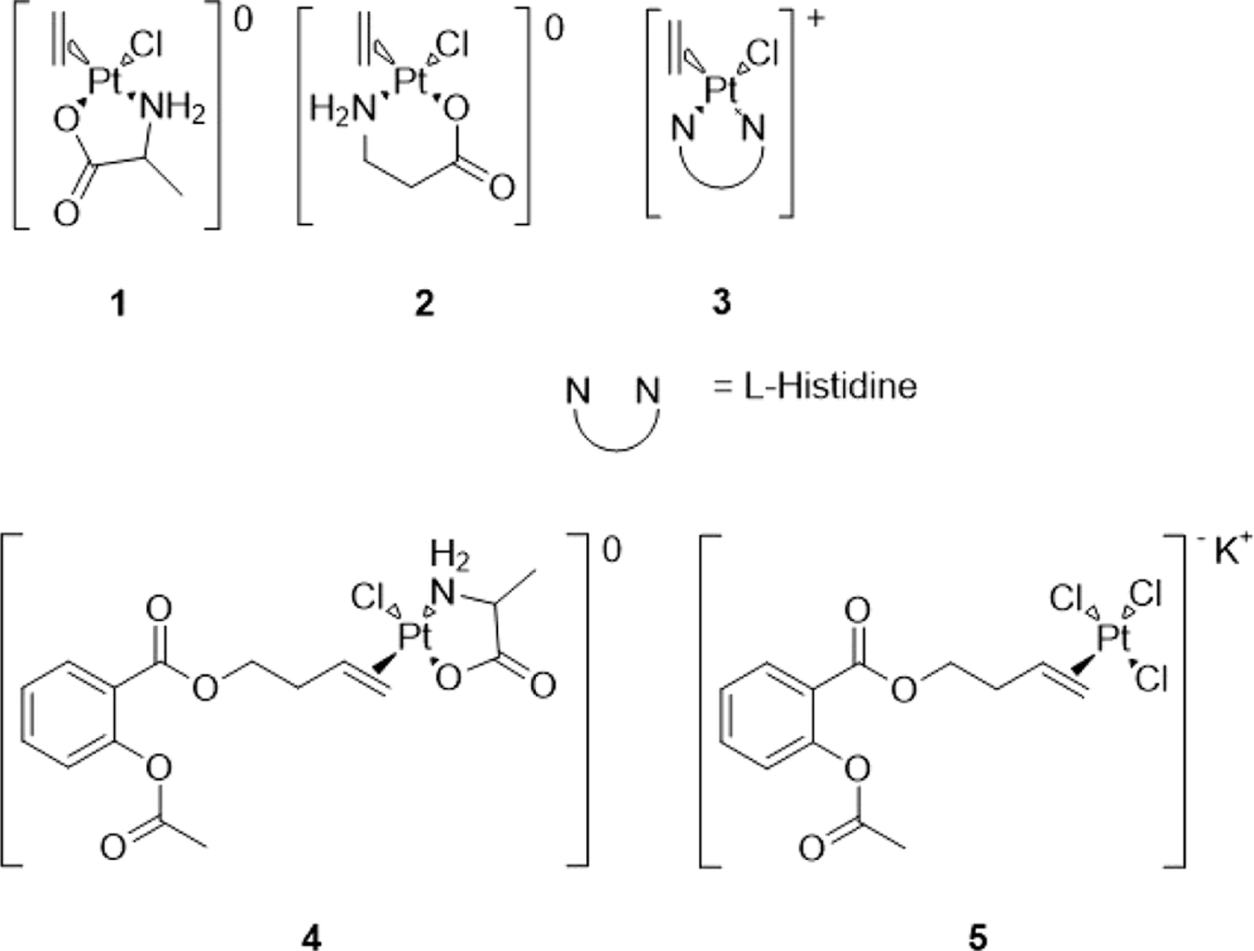
Structure of Complexes **1–4** with Three Different Coordination Motifs of the Amino Acid Ligands and the Structure of the Lead Compound (**5**) Based on the reaction conditions and on the net charge, we suspect **3** to have a chloride counter ion.

In our research group, potassium trichloro(ethylene)platinate(II) or Zeise’s salt (ZS), was investigated as a metal core for a possible new drug concept.^14^ A promising compound was obtained by combining ZS with acetylsalicylic acid (ASA). In a series of ASA-modified Zeise derivatives with different linker lengths, potassium {trichlorido[η^2^-(but-3-en-1-yl)-2-acetoxybenzoate]platinate(II)} (Pt-butene-ASA, **5**) (Scheme 1) exhibited high inhibition of COX-1, moderate inhibition of COX-2, and moderate activity against different tumor cell lines.^4,14^

These inspiring results oriented the research of our group on optimization of the lead structure. A desirable feature is an increased selectivity for COX-2 inhibition together with higher activity. We observed that, especially for the complexes with a propene linker, the presence of water is detrimental for the stability of these substances.^4,14^ Moreover, the reaction of complex **5** with sulfur-containing biomolecules, for example, ubiquitin or substance P, results in an immediate loss of the olefinic ligand. The main reason behind this degradation pathway is the strong *trans*-labilizing effect of the olefin, which causes the chlorido ligand in the *trans* position to be easily exchanged with a suitable nucleophile. If the new donor also exhibits a strong *trans*-labilizing effect, like, for example, the sulfur of methionine, fast cleavage of the platinum-olefin bond is observed.^15^ Considering the importance of solubility and stability in an aqueous environment for a drug candidate, the improvement of these parameters is essential and may also improve the cytotoxicity profile.

The degradation reaction of ZS in an aqueous environment is mediated by the exchange of the labile chlorido ligand *trans* to ethylene with a molecule of water.^16,17^ A possible strategy to protect this point of weakness is to exchange the *trans*-ligand with a less labile one, such as an amine. The use of polydentate ligands should further improve the stability of this kind of complex due to the chelating effect. Amino acids can be particularly suitable ligands for this purpose on account of their high biocompatibility and their hydrogen bond donor and acceptor groups, which may improve their water solubility. Moreover, they can be highly modulated by changing the side chain or the distance between the amino and carboxylic groups.

Panunzi et al. reported the first synthesis for three Zeise-type complexes bearing an amino acid as a chelating ligand in 1966. They chose glycine, racemic alanine, and β-phenylalanine and obtained the respective products as “yellow, stable, non-ionic complexes corresponding to the general formula: chloro-ethylene-amino-acid-platinum(II)”.^18^ A few years later, the configuration of the complex [Pt(C_2_H_4_)Cl(Gly)] was investigated, and it was found to be the *N-trans* isomer, referring to the position of ethylene.^19^ In 1970, the ethylene,*O-trans* isomer of the same complex was prepared to confirm the previous study.^20^ The mechanism and kinetics of the formation of the (ethylene,*N-trans*)-[Pt(C_2_H_4_)(Ala)Cl] complex were investigated in depth using UV spectroscopy at a fixed pH range (3–4) and at variable concentrations of the alaninato ion, controlled by modulation of the pH.^21^ About 10 years later, Erickson and Brower discovered a thermodynamic preference for the ethylene,*O-trans* isomer over the corresponding *N-trans* isomer for [Pt(olefin)Cl(Gly)] complexes using NMR spectroscopy.^22^ Cavoli et al. succeeded in 1986 in obtaining the first crystal structure of the (ethylene,*N-trans*)-[Pt(C_2_H_4_)Cl(β-Ala)] and, based on this and on the UV spectra, proposed the mechanism of formation of the complex as well as the kinetics of the reaction.^23^ An application of Zeise-type complexes with amino acid ligands in a biological or medical field has not been reported in the literature to date.

In the current study, we synthesized ZS derivatives with l-alanine, β-alanine, and l-histidine (complexes **1–3**, Scheme 1) and investigated their stability and biological properties. The ethylene on the ZS-alanine derivative was also successfully exchanged with a butene-modified ASA ligand (complex **4**, Scheme 1). We investigated both the effect of the chelating amino acid ligands on the stability profiles as well as the in vitro anticancer activity of the ZS derivatives on MCF-7 (breast cancer, COX positive), HT-29 (colon cancer, COX positive), MDA-MB-231 (breast cancer, COX positive), and A2780cis (ovarian cancer, COX negative, cisplatin-resistant) tumor cell lines. Also, the COX inhibitory activity of the complexes was tested to establish more insight into the structure activity relationship.

## Results and Discussion

### Synthesis and Characterization

Complex **1** was synthesized using the procedure reported by Fujita et al.,^20^ and cubic bright-yellow crystals were obtained within hours. The amount of solvent, namely, water, used for the reaction appears to be a critical factor for the isolation of the product since complex **1** is moderately soluble in water. When a slightly lower concentration was used (0.4 mmol of platinum precursor instead of 0.5 mmol), keeping the amount of solvent constant, no crystallization occurred within a short time (3–4 h). However, some colorless needle-shaped crystals formed after an extended period of time (5–6 weeks). The crystals obtained from both conditions were analyzed via single-crystal X-ray diffractometry.

**Figure 1:**
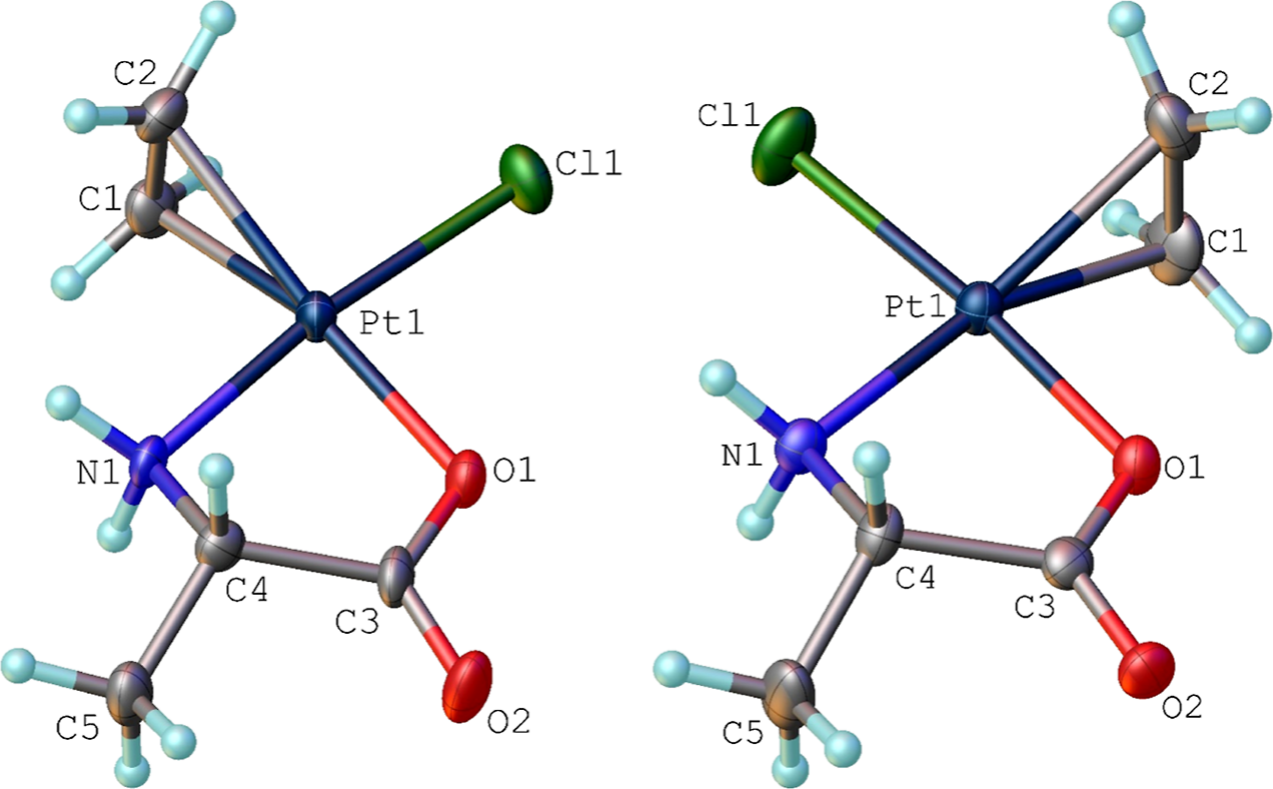
Oak Ridge Thermal-Ellipsoid Plot (ORTEP) of complexes **1b** (left, CCDC 2262096) and **1a** (right, CCDC 2262097).

The difference in concentration led to the formation of two isomers. In particular, the *N-trans* isomer (**1a**, Figure 1) was obtained by complying with the procedure reported in the literature, while the *O-trans* isomer (**1b**, Figure 1) crystallized much slower from the solution with a lower concentration. These results are also in agreement with the findings of Erickson and Brower,^22^ where the *N-trans* isomer was identified as the kinetic product and the *O-trans* isomer as the thermodynamic product of complexes of the type [PtCl(O–N)(olefin)].

Table 1 states the bond lengths and angles for isomers **1a** and **1b**, in comparison to the structures of ZS and the *N-trans* isomer of complex **2** (**2a**), both reported in the literature.

**Table 1:** Comparison of the Structural Information of the Two Isomers of Complex **1** (**1a** and **1b**), ZS, and Complex **2a**

	complex	ZS^24^	**1a**	**1b**	**2a**(23)
distances (Å)	C1=C2	1.37(3)	1.370(11)	1.382(7)	1.53(4)
	Pt–C1	2.121(19)	2.148(7)	2.125(7)	2.14(3)
	Pt–C2	2.134(19)	2.167(6)	2.136(7)	2.20(3)
	Pt-A (trans)	2.327(5)	2.054(5)	2.035(3)	2.09(2)
	Pt-A (cis)	2.314(7)	2.027(4)	2.037(4)	2.01(1)
	Pt–Cl	2.296(7)	2.2775(16)	2.3018(12)	2.277(8)
angles (deg)	Cl–Pt-A(trans)	90.2(3)	92.50(16)	92.10(9)	89.2(6)
	A(trans)-Pt-A(cis)	90.1(3)	82.44(19)	81.54(13)	92.1(7)

Both isomers **1a** and **1b** have a C=C bond length comparable to that for ZS, meaning that the total effect of the σ-bonding and of the π-back bonding is not significantly affected. The platinum–carbon bond length is slightly increased for isomer **1a**, which suggests that the coordination of ethylene to platinum is weaker. In comparison to complex **2a**, the Pt–C distances are similar, but there is a higher C=C distance in the isomer with β-alanine, indicating a higher π back-donation. The distances of nitrogen and oxygen to the metal center highlight the stabilization induced by the chelating effect in all the derivatives. In fact, these distances are significantly shorter compared to the corresponding positions in ZS. Also, a significant distortion in the chelating angle of **1a** and **1b** was observed, it is about 10° narrower than the ideal value for a square planar geometry. This difference is related to the formation of a 5-membered ring in the case of complex **1**. In fact, the corresponding chelating angle in **2a** is slightly above 90°, where the nonessential amino acid generates a 6-membered ring instead.

Table 2 lists the differences in chemical shifts (^1^H-, ^13^C-, and ^195^Pt-NMR) for isomer **1a** in comparison to ZS and l-alanine. The protons of the ethylene in **1a** are deshielded in comparison to the ones of ZS. This, together with the lower *J*_Pt–H_, suggests a weaker coordination for ethylene, in agreement with the Pt–C distances (see Table 1). As expected, more or less every signal referring to the coordinated amino acid is shifted downfield, due to the σ-donation of the amino group and the π-donation of the carboxylate.^25,26^ The only exceptions are the protons of the methyl group, which remain unaffected. The ^195^Pt-NMR spectrum shows that the metal ion is strongly deshielded, compared to ZS.

**Table 2:** Comparison of the Chemical Shifts of the ^1^H-, ^13^C-, and ^195^Pt-NMR Spectra of ZS, Complex **1a**, Complex **2**, l-Alanine, and β-Alanine (All Spectra Recorded in CD_3_OD)

	ZS	**1a**	**2**	l-alanine	β-alanine
C_2_H_4_ [ppm]	4.39	4.62	4.55	N/A	N/A
^2^*J*_H–Pt_ (C_2_H_4_) [Hz]	32.6	29.2	29.3	N/A	N/A
C_α_H [ppm]	N/A	3.78	N/A	3.58	N/A
–CH_3_ [ppm]	N/A	1.46	N/A	1.46	N/A
–CH_2_–NH_2_ [ppm]	N/A	N/A	3.19	N/A	3.07
–CH_2_–COO [ppm]	N/A	N/A	2.79	N/A	2.45
C_2_H_4_ [ppm]	68.9	76.6	75.4	N/A	N/A
C_α_H [ppm]	N/A	54.9	N/A	51.8	N/A
–CH_3_ [ppm]	N/A	19.4	N/A	17.3	N/A
–CH_2_–NH_2_ [ppm]	N/A	N/A	41.7	N/A	38.1
–CH_2_–COO [ppm]	N/A	N/A	34.7	N/A	34.3
–COO [ppm]	N/A	188.6	175.1	173.1	178.0
^195^Pt-NMR	–2769	–2557	–3032	N/A	N/A

Using the same reaction conditions as mentioned above, it was not possible to obtain complex **2**. Different strategies were tested (different bases, isolation of the potassium β-alaninate before the reaction with ZS), but in all cases, the solution turned brownish dark very quickly, indicating degradation of the precursor. However, an approach without the employment of a base was successful. Here, ZS and β-alanine were mixed in cold water and yielded the product as a bright yellow powder within 15 min. It was not possible to obtain crystals suitable for the analysis with X-ray diffractometry, but the complex was fully investigated via NMR spectroscopy (see Table 2).

The protons of the ethylene in **2** are slightly downfield shifted compared to those in ZS, similar to complex **1a**, but to a lesser extent. The coupling constant between platinum and the olefinic protons in **2** is almost equal to the one of **1a**. However, in contrast to **1a**, the ^13^C signal of the carboxylate in **2** is more shielded than that for the free β-alanine. Also, the platinum signal is shifted upfield in comparison to ZS.

**Scheme 2:**
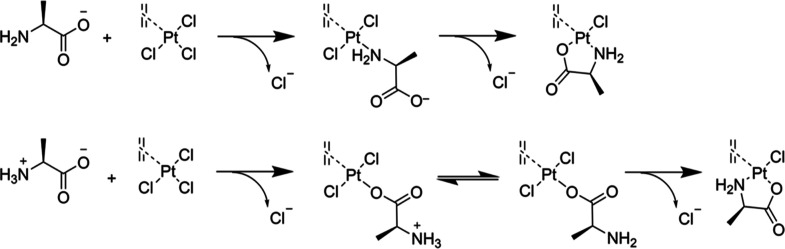
Proposed Mechanism for the Synthesis of the *N-trans* Isomer **1a** (Top) and the *O-trans* Isomer **1b** (Bottom)

Based on these findings, we conclude that complexes **1a** and **2** were obtained as different isomers. This highlights the critical role of the base on the selectivity of this reaction (Scheme 2). To confirm this hypothesis, we decided to follow the synthesis of complex **1** via NMR spectroscopy (^1^H-, ^13^C-, and ^195^Pt-, Figures S30–S32, respectively) with 1 equiv and without potassium bicarbonate. For this purpose, the reaction was set up with deuterium oxide as a solvent. The results are summarized in Table 3.

**Table 3:** Comparison of the Chemical Shifts Obtained from ^1^H-, ^13^C-, and ^195^Pt-NMR of l-Alanine, ZS, and Complex **1** Obtained with and without Base (All Spectra Recorded Using D_2_O as Solvent)

		C_2_H_4_	C_α_H	–CH_3_	–COO	Pt
l-Ala	^1^H [ppm]		3.77	1.46		
	^13^C [ppm]		50.4	16.1	175.7	
ZS	^1^H [ppm]	4.66				
	^13^C [ppm]	70.5				
	^195^Pt [ppm]					–2796
**1b** w/o base	^1^H [ppm]	4.68	4.00	1.58		
	^13^C [ppm]	71.1	49.8	15.9	174.3	
	^195^Pt [ppm]					–2793
**1a** w base	^1^H [ppm]	4.77	4.00	1.52		
	^13^C [ppm]	77.4	53.6	18.3	189.6	
	^195^Pt [ppm]					–2535

The signals of the amino acid protons and carbons are shifted in both cases, confirming the coordination of the amino acid to platinum. The signals of the ethylene, carboxylate, and platinum are significantly shifted when a base is used, and they correspond to the signals of the *N-trans* isomer **1a**. In the absence of a base, the signals of the carboxylic carbon and of the platinum are both shifted to a lower frequency, while they are shifted to a higher frequency when a base is used (Table 3). This evidence suggests that two different isomers **1a** and **1b** (Figure 1) were obtained, depending on the presence or absence of a base. Comparing these results with the chemical shifts as reported in Table 2, a similar trend for the isomer **1b** and complex **2** suggests that it was obtained as the *O-trans* isomer (**2b**).

**Figure 2:**
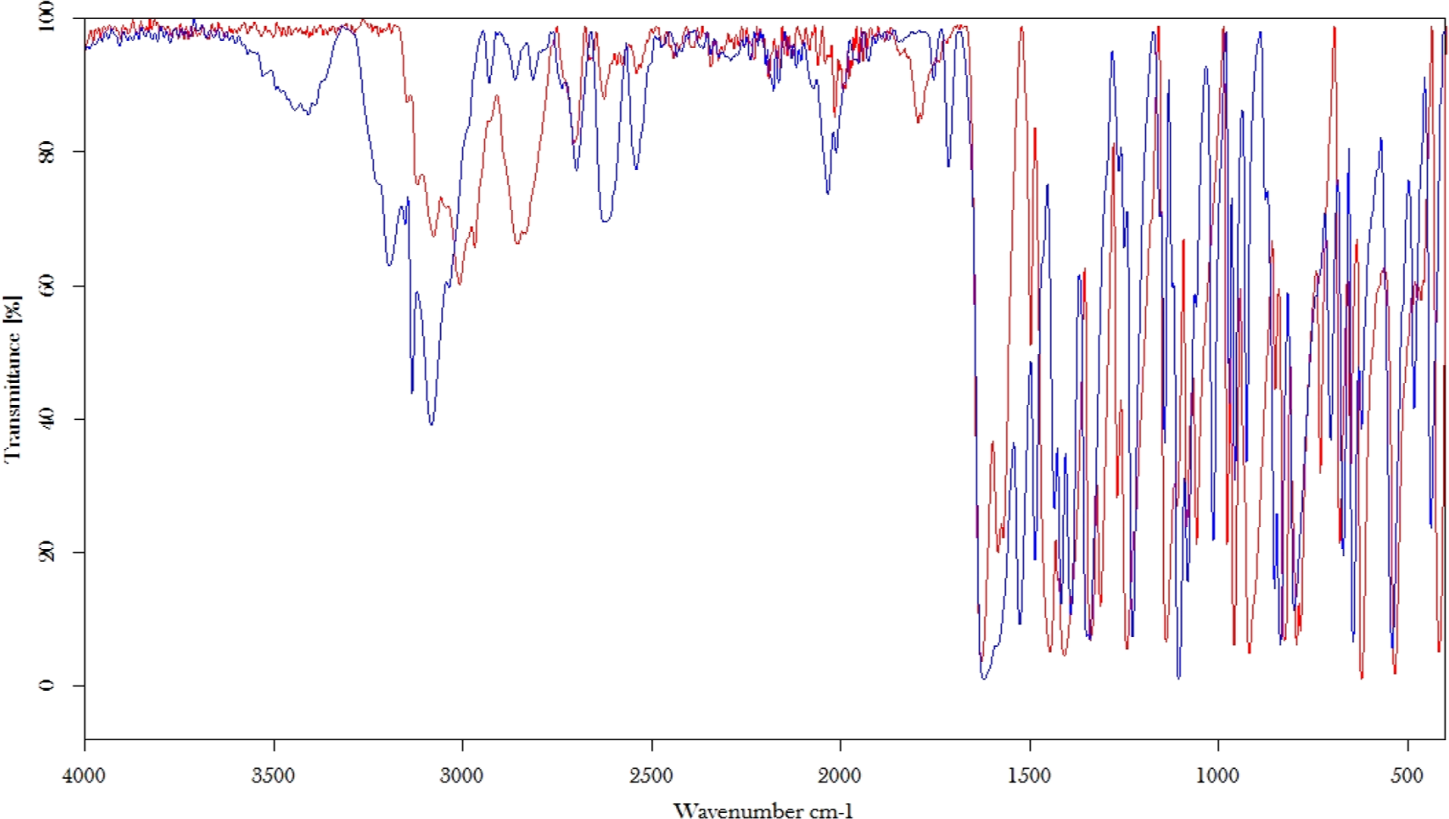
IR spectra of l-histidine (red) and complex **3** (blue) superimposed.

The characterization and structure elucidation for complex **3** are strongly affected by its solubility. Despite trying an array of usual solvents, no solution with a suitable concentration for characterization via NMR spectroscopy was obtained. A partial formation of oligo-nuclear species might explain the bad solubility. The comparison of the IR spectra of complex **3** and of l-histidine (Figure 2) recorded from the neat solids proves that the amino acid is coordinated to the platinum core, as demonstrated by the shifts of the bands. In particular, two new bands at about 3450 and 1715 cm^–1^ can be assigned to the O–H stretching and to the –C=O stretching of the carboxylic group, respectively, indicating that the amino acid is not in the zwitterionic form. The complex band created by the stretching of the imidazole and the side chain’s C–H stretching is shifted from about 3000–3100 to about 3100–3200 cm^–1^ as a consequence of the coordination. The band around 2800 cm^–1^ in the histidine spectrum is also affected by coordination to the platinum.

Further proof for the identity of complex **3** was obtained through the HR-MS spectrum, where a characteristic cluster of signals at *m*/*z* 412–416 is visible and in agreement with the calculated isotopic distribution of complex **3** (see Figure S16).

**Figure 3:**
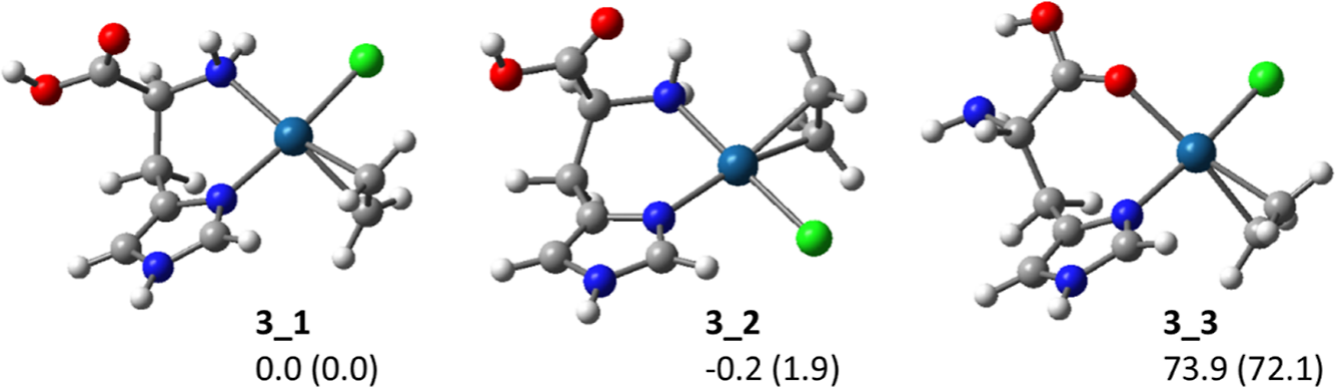
Optimized structures at the B3LYP level of isomers **3_1–3** of complex 3. Relative enthalpies (free energies) at 298 K calculated at the M06-2X level are reported in units of kJ mol^–1^. Both thermodynamic and spectroscopic evidence agree in indicating **3_1** and **3_2** as the only isomeric forms assayed.

In theory, histidine has four possible donor sites that can coordinate to platinum: the two N-atoms of the imidazole group (N_π_ and N_τ_ being the closer and farther to the amino acidic chain, respectively), the carboxylic oxygen, and the amino nitrogen (N_a_). However, binding of the metal to N_τ_ does not allow the chelation of histidine to the metal due to steric constraints;^27,28^ therefore, three isomers presenting the N_π_ atom interacting with platinum but differing for the other coordinating atoms were considered for further investigation. Figure 3 depicts the optimized structures of the three isomeric forms of complexes **3** (**3_1–3**). The two lowest-energy structures show the amino nitrogen bound to platinum and are practically isoenergetic. They differentiate for the relative position of the ligand in the complex. In particular, **3_1** shows the amino group of histidine bound to platinum in *trans* position to the ethylene ligand, vice versa, **3_2** presents the imidazole N_π_ atom in *trans* to C_2_H_4_. Isomer **3_3** simulates the coordination of the histidine carboxylic group to platinum, but the structure is significantly higher in energy, with a relative Gibbs free energy of 73.9 kJ/mol. To unequivocally assign the structure of complex **3** to a specific isomer, IR multiple-photon dissociation (IRMPD) spectroscopy was exploited.^27,29−31^ Mass selected ions were submitted to IR photons of variable energy. Figure S17 shows the IRMPD spectrum together with the calculated spectra of isomers **3_1–3**. The experimental bands are in good agreement with the calculated vibrational features of both **3_1** and **3_2**, allowing us to attribute the structure of the sampled ions to either one of them or a combination of the two and to confirm that histidine coordinates through the N_π_ and N_a_ atoms to Pt(II). Additional details on the assignment of vibrational bands are reported in the Supporting Information in Table S1 and in the description in Figure S17. Interestingly, the attributed structures **3_1** and **3_2** both present an open coordination site for an incoming platinum atom: the N_τ_ atom. The resulting dinuclear and polinuclear species may be responsible for the reduced solubility of the product of the synthesis; however, no clear indication of their formation has been gathered at present.

Complex **4** was synthesized following the published procedure for complex **5**^4^, and using complex **1a** as the platinum precursor instead of ZS. NMR spectroscopy reveals a single set of signals for the coordinated amino acid and only one peak in the ^195^Pt-NMR; hence, complex **4** was obtained as the *N-trans* isomer only. In general, the peaks of the olefinic protons of **4** are deshielded in comparison to the complex **5**, confirming the trend of the amino group in *trans* to destabilize the coordination of the olefin. Moreover, the electron density on the metal ion is lower in the case of complex **4**, as highlighted by the chemical shift toward a lower field compared to complex **5**.

### Stability

The structure model^32^ and the chemistry of ZS in aqueous solution^17,33,34^ were investigated in depth in the past. The pronounced *trans* effect exerted by the ethylene causes the *trans*-chlorido ligand to be far more labile than the *cis*-chlorido ligand.^34^ The kinetics of the exchange reaction of the *trans*-chlorido ligand with a water molecule are so fast that ZS is converted to the *trans*-aquo complex quantitatively within 2 min of dissolution in water.^33^ This intermediate is stable over weeks in acidic conditions but undergoes quick reductive degradation in neutral or basic solutions.^33^ The nature of the intermediates and products of these degradation pathways is still elusive.^17^

### Water

The stability of complexes **1–4** was tested in an aqueous solution, to investigate how the different ways of amino acid coordination impacts aqueous degradation (see Table 4). The stability of complex **5** was previously measured in a 50% methanolic solution.^4^ The half-life of this compound under the abovementioned conditions was found to be 69.6 ± 3.0 h.

**Table 4:** Comparison of the Half-life Time (τ_1/2_) Determined in an Aqueous Environment (with or without TMG) for Complexes **1a**, **2b**, **3**, and **4**^a^

	**1a** (h)	**2b** (h)	**3**	**4** (h)
water	>72	1.7 ± 0.2	N/A	>72^a^
TMG	24.3 ± 2.6 h	0.77 ± 0.08	12.0 ± 3.0 h	>72

a These values are obtained from a 50% methanol solution.

Complex **1a** is remarkably stable in water, with a half-life time greater than 72 h. No degradation products were detected at the electropherograms, which suggests precipitation of the complex or the degradation products.

Complex **2b**, however, showed much lower stability (τ_1/2_ = 1.7 ± 0.2 h) and fast conversion into another species with a higher effective mobility (−6.13 × 10^–5^ vs −3.42 × 10^–4^ cm^2^/s V for **2b**).

**Scheme 3:**
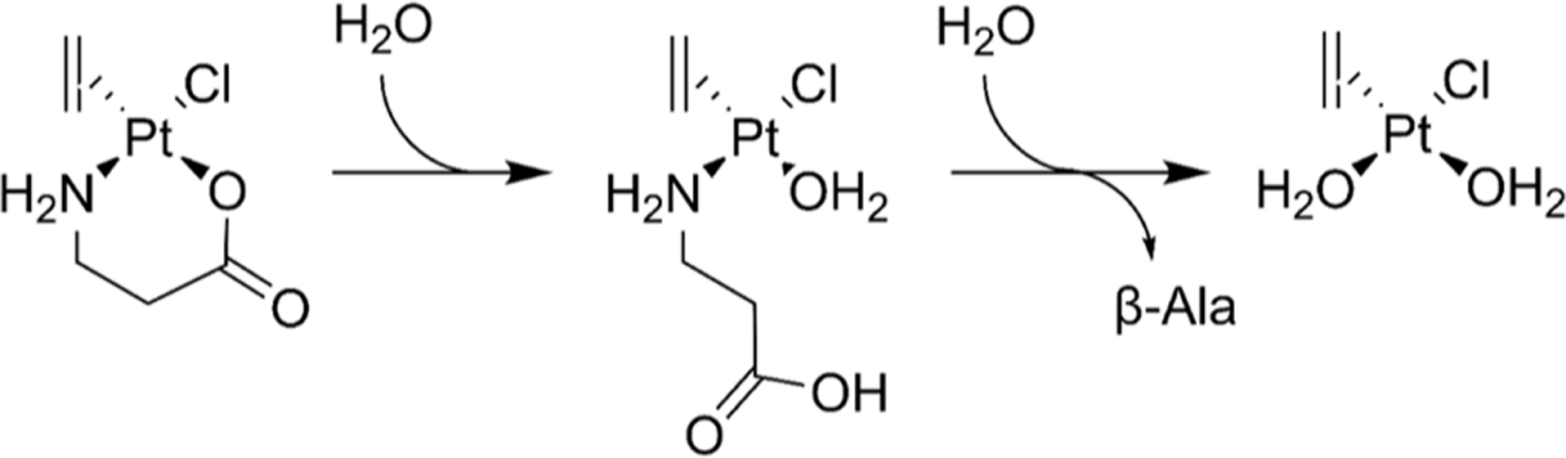
Proposed Reaction of Complex **2b** in an Aqueous Environment

To better understand the nature of this intermediate, the reaction of complex **2b** with a large excess of water was followed by NMR spectroscopy (50% D_2_O, Figures S35 and S36). The results highlight how the signal of the methylene in the α-position to the carboxylic group is the first to decrease. Simultaneously, a peak corresponding to the same methylene of the free amino acid is forming and increasing over time. A similar process is observed for the methylene in the α-position to the amino group, but the reaction is slower in this case. The signal of the ethylene ligand shows fewer changes in intensity and no overall loss of ethylene. Only two signals were detected in the ^195^Pt-NMR experiments, probably for the intermediate and the final degradation product, as shown in Scheme 3. Based on the spectroscopic data, we conclude that the carboxylic group *trans* to the ethylene detached from the platinum in a first step, followed by the cleavage of the coordination bond between the amino group and the metal ion, forming the diaquo complex.

Due to the solubility issues of complex **3**, it was not possible to obtain any information about the stability profile in purely aqueous conditions, and a different approach was required (see below, TMG).

The stability profile of complex **4** is comparable to that of its precursor (complex **1a**). However, the release of olefin in an aqueous environment was detected to an extent of about 20% after 72 h. The chelating amino acid enhances the stability of complex **4** (>72 h) in comparison to compound **5** (69.6 ± 3.0 h). Also, complex **4** exhibited a better solubility in water.

### Trimethylglycine

Kadokawa et al.^35^ reported that a highly concentrated solution of trimethyl glycine (TMG, also known as betaine) is able to improve the solubility of cisplatin in water without affecting the pharmacological proprieties of this drug. The mechanism behind this effect seems to lie in the hydrogen bond acceptor property of the carboxylate of TMG, which establishes hydrogen bonds with the amino groups of cisplatin. Betaine is a nontoxic compound and is highly soluble in water. On the basis of this study, we decided to test the solubility of **3** in a highly concentrated (50% w/v) TMG aqueous solution. The platinum compound was soluble enough to allow investigations with the established protocol for capillary electrophoresis. TMG has a strong absorption at low wavelengths, and therefore, a wavelength of 230 nm was chosen instead of 195 nm for recording the electropherograms with TMG.

Complexes **1a**, **2b**, **3**, and **4** were dissolved in a 50% w/v TMG solution, diluted to a final TMG concentration of 25%, and followed over time with the established CE protocol (see Table 4).

Complex **3** exhibited a half-life time of 12.0 ± 3.0 h under these experimental conditions. A degradation product was detected at a lower effective mobility (−4.19 × 10^–4^ vs −2.90 × 10^–4^ cm^2^/s V for **3**). The identification of this intermediate was not possible due to solubility issues. The stabilities of the other organometallic complexes follow the same trend as for the samples in aqueous solution (τ_1/2_(**1a**) = 24.3 ± 2.6 h, τ_1/2_(**2b**) = 0.77 ± 0.08 h, and τ_1/2_(**4**) > 72 h).

In general, we observed that stability is negatively affected by the use of TMG. A possible explanation for this phenomenon is that the 50% TMG solution is slightly alkaline (pH = 8.28 ± 0.04) and the basic environment can probably catalyze the degradation reaction of the Zeise-derivative complexes. The deprotonation of the amino groups involved in the coordination of amino acids can also play a role.

To test whether TMG can coordinate to the platinum center, ZS was dissolved in a solution of TMG in deuterium oxide, and ^195^Pt-NMR spectra were recorded overtime (Figure S37). Based on these data, TMG does not interfere with the inner coordination sphere of ZS-derivatives.

Taken together, the results collected from the experiments in water and concentrated TMG solution suggest that the chelating effect exerted by the amino acid stabilized the labile position *trans* to the olefin. However, the isomerization strongly affects the stability of the entire complex. In fact, the *O-trans* complex exhibits a lower half-life time compared to the *N-trans* complexes due to the substitution of the oxygen trans to the olefin.

### Biological Tests

#### Cytotoxic Effects against Cancer Cell Lines

In order to determine the antitumor activity of the platinum complexes, in vitro cytotoxicity tests were performed. Compounds **1–4** were tested with cisplatin as a reference on the breast cancer cell line MCF-7 and the colon carcinoma cell line HT-29. Complexes **1**, **4**, and **5**, as well as the ligand (but-3-en-1-yl 2-acetoxybenzoate, **6**) were additionally evaluated on MDA-MB-231 cells and A2780cis cells, also using cisplatin as a reference. The metabolic activity as an indicator of cytotoxicity was determined by a classical 3-(4,5-dimethylthiazol-2-yl)-2,5-diphenyltetrazoliumbromid (MTT) assay.

The amino acid derivatives of ZS **1–3** showed almost no cytotoxicity, with an IC_50_ value above 100 μM against all of the cancer cell lines tested. Compound **4**, instead, was found to be active against the two cell lines, with IC_50_ values comparable to cisplatin (Table 5).

**Table 5:** Metabolic Activity of Complexes **1–4** against MCF-7 and HT-29 Tumor Cell Lines

	metabolic activity IC_50_ [μM]	
compound	MCF-7	HT-29
**1**	>100	>100
**2**	>100	>100
**3**	>100	>100
**4**	22.51 ± 3.23	20.63 ± 0.11
cisplatin (IC_50_)	29.04 ± 4.28	13.93 ± 5.16

**Figure 4:**
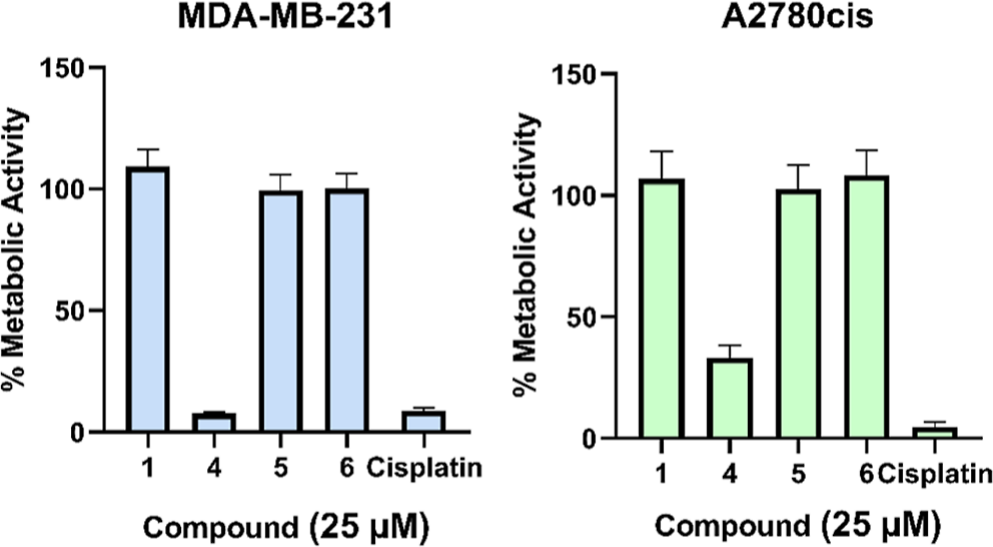
Metabolic activity of **1**, **4**, **5**, and **6**, and cisplatin at 25 μM on MDA-MB-231 and A2780cis cells; the mean of five independent experiments ± SEM. The metabolic activity without compounds was set at 100%. The IC_50_ values for cisplatin are reported as reference.

Compounds **1**, **4**, **5**, and **6** were tested on two further cell lines, MDA-MB231 and A2780cis, at 25 μM. As demonstrated in Figure 4, only the stability-optimized complex **4** was highly cytotoxic, with a metabolic activity of 7.71% similar to that of cisplatin (8.71%). At A2780cis, its activity was slightly weaker (33.13 vs 4.71%). The difference between the activity of complex **4** and compounds **1**, **5**, and **6** was statistically significant in both cell-lines. Noteworthy, the cytotoxic activity of complex **4** was statistically comparable to that of cisplatin against both MDA-MB-231 and A2780cis cancer cells.

**Figure 5:**
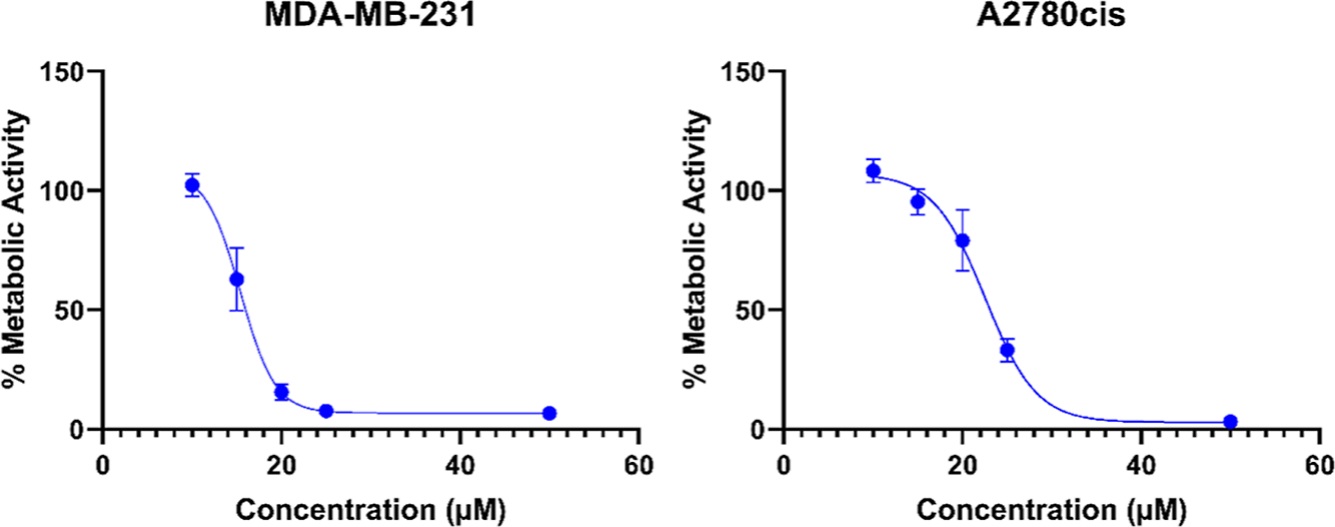
Metabolic activity profile of **4** on MDA-MB-231 and A2780cis cells; the mean of five independent experiments ± SEM.

Therefore, concentration-dependent cytotoxicity evaluations for compound **4** were performed (Figure 5), resulting in IC_50_ values comparable to the values obtained for cisplatin in each cell line tested (Tables 5 and 6). A comparison with the data reported for other ZS-ASA derivatives^4,36^ confirms the enhanced potency of complex **4** against HT-29 and MCF-7 since no other derivative had an IC_50_ value comparable with cisplatin.

**Table 6:** IC_50_ Values Calculated for Complex **4** and the Corresponding Values of Cisplatin (Reference)

	metabolic activity IC_50_ [μM]	
compound	MDA-MB-231	A2780cis
cisplatin	13.27 ± 0.87	14.81 ± 1.23
**4**	15.41 ± 0.74	22.54 ± 0.97

The increased stability of complex **4** probably plays a role in its increased cytotoxicity compared to the precursor complex **5**, which possesses a labile chlorido ligand trans to the olefin, representing a reactive site that mediates the activity of the complex. This position can undergo substitution by peptides or proteins.^15^ The respective position in complex **4** is stabilized by the amino acid ligand. Slow release of the olefinic ligand has been observed for complex **4**, but not **5**. Also, compound **4** is neutral, whereas compound **5** is negatively charged, which will likely impact the cellular uptake of these platinum complexes.

**Figure 6:**
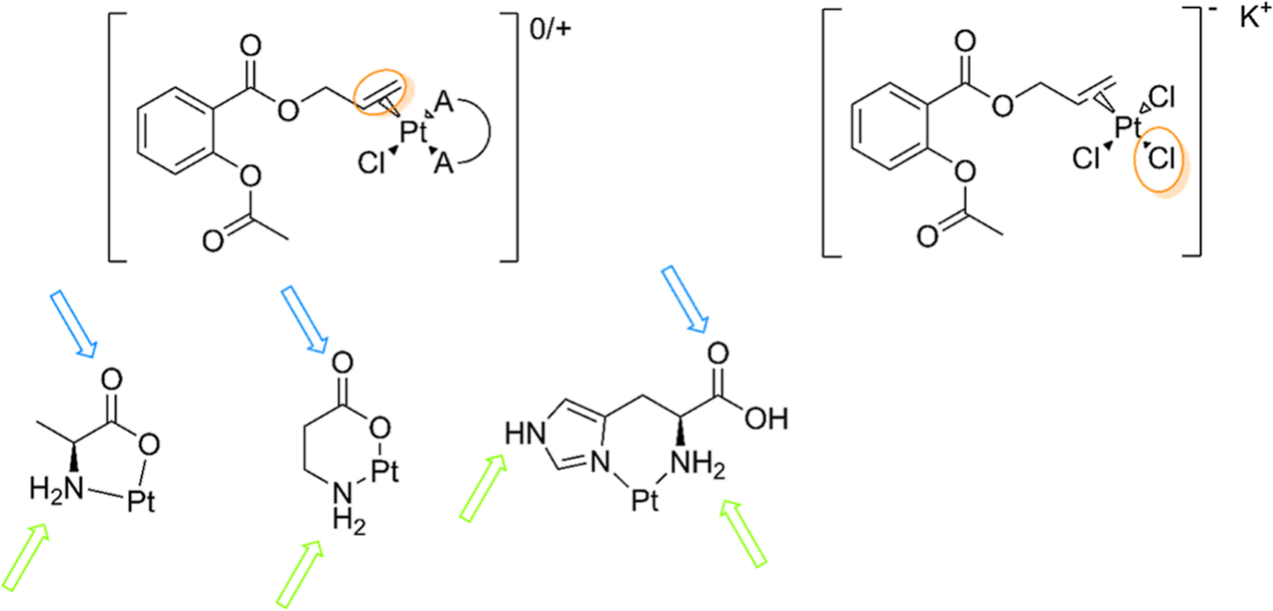
Overview of SAR considerations. Most reactive sites for complexes **4** and **5** are highlighted in orange. H-bond donors and acceptors on the amino acidic ligands are highlighted in green and blue, respectively.

The improved in vitro performance of **4** over **5** may be explained by several factors. The cellular uptake might be enhanced due to the different charge of the complex and enhanced lipophilicity (see Table 7). The bioavailability may be improved due to the presence of H-bond donors and acceptors of the amino acidic ligand (Figure 6), and possibly a synergistic effect of the platinum core and the olefinic ligand occurs.

**Table 7:** Coefficient of Partition between Buffer and Micelles (*k*′) Calculated from MEKC Experiments and the Predicted Log *P*(o/w) Values Obtained from Two Different Models^a^

		predicted log *P*	
	*k*′^37^	ASNN5 model^38^	Osiris Property Explorer^39^
compound	average	log *P*	log *P*
**1a**	0.629 ± 0.009	–0.93 ± 0.78	–0.78
**2b**	3.099 ± 0.149	–1.3 ± 0.78	–0.77
**3**	2.392 ± 0.185	–1.1 ± 0.78	–1.47
**4**	3.383 ± 0.016	1.8 ± 0.78	1.15
**5**	3110	1.2 ± 0.78	1.93
**6**	4050	0.85 ± 0.78	2.69

a Every value of *k*′ was calculated from the results of three independent MEKC experiments (except for compounds **5** and **6**, for which only one experiment was available).

The lipophilicity of the tested compounds was estimated on the basis of MEKC experiments similar to Herbert and Dorsey^37^ (see Table 7). The values estimated in this way were compared with theoretical values obtained from two different methods: for platinum complexes, a previously established method^38^ was used, while for the organic ligand, the Osiris Property Explorer^39^ was employed. For compounds **1a**, **4**, **5**, and **6**, a significant correlation (*p* = 0.01) was found between the estimated micelles-water partition coefficient and the theoretical log(*P*_ow_), however, compounds **2b** and **3** do not fit into the trend.

Comparing the biological activity with the stability profile, the estimated and theoretical lipophilicity, and the solubility in aqueous solutions resulted in no significant correlation. We conclude that adjusting the molecular reactivity of complex **4** compared to **5** is responsible for the improved biological activity.

#### COX Inhibition

Metal complexes containing an ASA moiety have been reported as inhibitors for COX-1/-2 previously.^4,14,40−44^ Hence, an in vitro COX-1/-2 inhibition assay was performed to gain some insight into the mode of action of platinum complex **4**. Hereby, the isolated enzymes were treated with final concentrations of 10 and 25 μM of substances **1**, **4**, **5**, and **6**. Compounds **1**, **4**, and **5** dose-dependently inhibited COX-1 and COX-2 (Figure S38).

**Figure 7:**
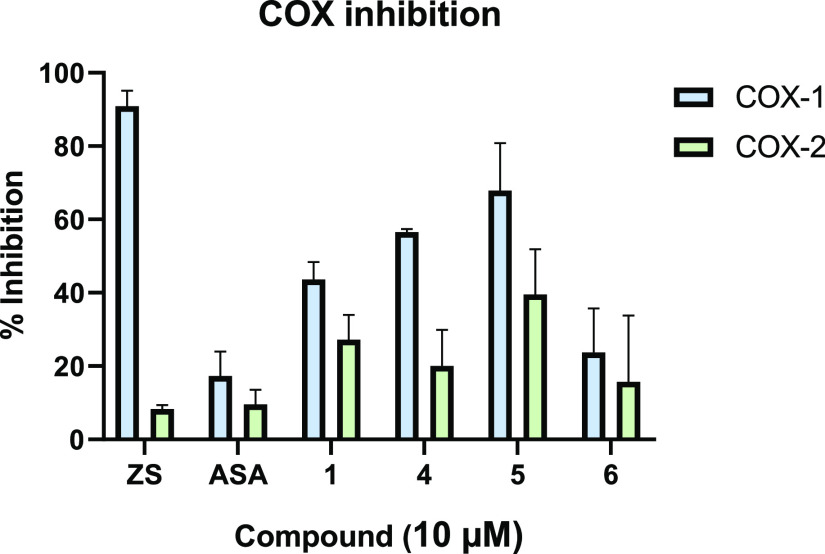
Inhibition of COX-1/-2 isoenzymes by ZS, ASA, **1**, **4**, **5**, and **6** at 10 μM; the mean of three independent experiments ± SEM.

Ligand **6** inhibited the COX isoenzymes to a low extent, which suggests that the platinum ion is important for inhibition of the respective enzymes. All complexes **1**, **4**, and **5** selectively inhibited COX-1. However, compared to ZS, which is a selective COX-1 inhibitor (COX-1 inhibition at 10 μM of 90.83% and COX-2 inhibition of 8.30%), and ASA (COX-1 inhibition at 10 μM of 17.33% and COX-2 inhibition of 9.33%), a shift toward the inhibition of COX-2 can be observed for **1**, **4**, and **5** (COX-2 inhibition at 10 μM of 27.23, 20.04, and 39.56%, respectively. See Figure 7).

The data obtained do not show any statistically relevant correlation between the cytotoxic activity and COX-1 or COX-2 inhibition (Table S18). Complex **4** had good efficacy against the COX-2 expressing MDA-MB-231 and HT-29 cell lines but was also effective on MCF-7 and A2780cis cells that express only limited amounts of COX-2. These observations suggest that the inhibition of COX-2 may play a role in the mode of action of complex **4**, but this enzyme does certainly not represent the exclusive target for this potential antitumor agent. Hence, we assume additional targets are involved in the mode of action of compound **4**.

## Conclusions

This study addresses the stability issues known to affect platinum(II) complexes and especially ZS derivatives. Amino acids were successfully employed as chelating agents to improve the aqueous stability and the cytotoxicity profile of a potential platinum(II) anticancer drug.

Three different amino acids, resulting in different coordination motifs, were chosen to investigate the influence on aqueous stability. Best results were achieved with l-alanine coordinating with the amino group in trans position to the ethylene, forming a 5-membered ring with platinum, having a bite-angle of about 80°, as elucidated by the X-ray diffraction spectroscopy. The ethylene was then exchanged with an ASA-containing ligand to obtain stability-optimized complex **4**. These structural changes also led to improved water solubility and enhanced cytotoxic activity in comparison to the previously published complex **5**. The comparison of the potency of complex **4** with the data obtained for complexes **1** and **5** highlights how both the olefinic and the amino acidic ligands are necessary but not sufficient for the improved biological results, and only the simultaneous presence of both groups grants in vitro activity comparable to that of the reference substance, cisplatin.

Particularly, the amino acid ligand provides higher lipophilicity and H-bond donors and acceptors, which will likely result in increased cellular uptake and enhanced bioavailability. The highly reactive chlorido ligand in complex **4** is replaced by a less reactive and chelating amine, which influences the molecular mode of action.

No significant improvement in the COXs inhibition was observed for complex **4**, compared to complex **5**. Moreover, complex **4** exhibits better cytotoxic activity on both COX-2 positive and negative cancer cell lines. Its cytotoxicity on MDA-MB-231, in particular, was almost identical to that of cisplatin.

However, the exact mechanism of action of this complex is still elusive and will be the subject of further investigations. Considering the results obtained so far, we expect that complexes bearing an amino acid as a chelating ligand may be more resistant against nucleophilic attack to the platinum center from biological species, such as glutathione or other sulfur-containing species. This aspect is also important to shed light on the improved in vitro activity of complex **4** and investigations are planned to fully understand the potential behind this aspect.

## Experimental Section

### Materials

All chemicals were purchased from Sigma-Aldrich, ABCR chemicals, Fluka, or Euriso-Top and used as received. Solvents were purchased in the appropriate purity and used as received. Water was deionized using a Millipore Milli-Q Gradient A10 Water Purification system (Merck Millipore, Billerica, MA, USA). ^1^H-, ^13^C-, and ^195^Pt-NMR spectroscopy was performed on a Bruker AVANCE 4 Neo instrument (^1^H resonance frequency: 400 MHz). For the correct assignment of the signals, [^1^H,^1^H]-COSY, [^1^H,^13^C]-HSQC, and [^1^H,^13^C]-HMBC 2DNMR experiments were carried out. Chemical shifts (δ) were given in parts per million (ppm). The coupling constants (*J*) were given in Hertz (Hz). Chemical shifts of ^1^H- and ^13^C NMR experiments were referenced using the center of the internal residual peak of the solvent signal, which was related to tetramethylsilane as δ = 3.31 (^1^H NMR) and δ = 49.00 (^13^C NMR) for CD_3_OD, δ = 2.05 (^1^H NMR), δ = 29.84 (^13^C NMR) for (CD_3_)_2_CO, and δ = 4.79 (^1^H NMR) for D_2_O.^45^ High-resolution electrospray ionization mass spectrometry (HR-ESI-MS) was performed using an Orbitrap Elite mass spectrometer (Thermo Fisher Scientific, Waltham, MA, USA). IR spectra were recorded with a Bruker ALPHA FT-IR spectrometer equipped with a Platinum-ATR module (diamond crystal) on a neat solid sample, if not stated differently. Capillary electrophoresis (CE) experiments were carried out on a 3D-CE system (Agilent, Santa Clara, CA, USA), which was equipped with an autosampler, a diode array detector (DAD), and a temperature-controlled capillary compartment. Agilent fused-silica capillaries (75 μm inner diameter; 56 cm effective length, 64.5 cm total length) were purchased from VWR (Vienna, Austria). Purity determination and stability investigations were performed with CE experiments. All compounds were >95% pure by CE (see Figures S26–S29).

### Synthesis

#### (Ethylene,*N-trans*)(l-alaninato-N,O)chlorido(η^2^-ethene)platinate(II) (*N-Trans*)[PtCl(l-Ala)(C_2_H_4_)] (**1a**)

This complex was synthesized using the procedure reported by Fujita et al.^20^ ZS (0.5 mmol, 1.0 equiv) and l-alanine (0.5 mmol, 1.0 equiv) were suspended in 1.5 mL of deionized water. The mixture was cooled via an ice bath, and after 5 min, potassium bicarbonate (0.5 mmol, 1.0 equiv) was added in small portions while stirring. After complete dissolution of l-alanine, the mixture was heated to 40 °C for 5 min. After this period of time, the solution was allowed to cool at 0 °C in an ice bath until crystallization occurred (about 6 h). The flask was sealed and put into the fridge for 2 nights. The crystals were then filtered and washed with small amounts of cold water, cold ethanol, and cold diethyl ether.

Yield: 54% as bright yellow crystals; purity: 97.28%; ^1^H NMR (400 MHz, CD_3_OD): δ (ppm) 4.62 (s, 4H, CH_2_=CH_2_, ^2^*J*_H–Pt_ = 29.2 Hz), 3.78 (q, ^2^*J* = 7.2 Hz, 1H, C_α_H), 1.46 (d, ^2^*J* = 7.2 Hz, 3H, –CH_3_); ^13^C NMR (101 MHz, CD_3_OD): δ (ppm) 188.8 (–COO), 76.6 (CH_2_=CH_2_), 54.9 (C_α_), 19.4 (–CH_3_); ^195^Pt-NMR (86 MHz, CD_3_OD): δ (ppm) −2556. Elemental Anal. Calcd for C_5_H_10_ClNO_2_Pt: C, 17.32; H, 2.91; N, 4.04. Found: C, 17.26; H, 2.97; N, 3.68; HR-MS: [M + H]^+^ exp, 347.0116, calcd, 347.0121.

#### (Ethylene,*O-trans*)(β-alaninato-N,O)chlorido(η^2^-ethene)platinate(II) (*O-trans*)[PtCl(β-Ala)(C_2_H_4_)] (**2b**)

Complex **2** was obtained using a method similar to the one reported by Weninger et al.^46^

ZS (0.4 mmol, 1.0 equiv) was dissolved in a vial in 1 mL of cold water. β-Alanine (0.4 mmol, 1.0 equiv) was dissolved in 1 mL of cold water and added in one portion to the platinum solution under stirring. The mixture was cooled in an ice bath. After 15 min, a solid precipitated, which was collected by filtration, washed several times with cold water, and then dried in vacuo to obtain the desired product.

Yield: 43% as a pale-yellow powder; purity: 96.00% ^1^H NMR (400 MHz, CD_3_OD): δ 4.55 (s, 4H, CH_2_=CH_2_, ^2^*J*_H–Pt_ = 29.3 Hz), 3.19 (t, ^2^*J* = 6.6 Hz, 2H, NH_2_–CH_2_–CH_2_, ^3^*J*_H–Pt_ = 16.10 Hz), 2.79 (t, ^2^*J* = 6.6 Hz, 2H, CH_2_–CH_2_–COO); ^13^C NMR (101 MHz, CD3OD): δ 175.1 (–COO), 75.4 (CH_2_=CH_2_), 41.7 (NH_2_–CH_2_–CH_2_), 34.7 (CH_2_–CH_2_–COO); ^195^Pt-NMR (86 MHz, CD3OD): δ −3022. Elemental Anal. Calcd for C_5_H_10_ClNO_2_Pt + HCl: C, 15.67; H, 2.89; N, 3.66. Found: C, 15.59; H, 2.92; N, 3.62; HR-MS: [M + H]^+^ exp, 347.0120, calcd, 347.0121.

#### (l-Histidinato-N,N)chlorido(η^2^-ethene)platinate(II) [PtCl(His)(C_2_H_4_)] (**3**)

155 mg of ZS was dissolved in 0.75 mL of water in a vial protected from light. 62 mg of l-histidine was suspended in 1 mL of water and added at 0 °C. The suspension was allowed to stir at 0 °C for 15 min. The reaction mixture was then filtered, washed 3 times with cold water, and air-dried.

Yield: 78% as pale-yellow powder; purity: 96.16%; NMR characterization was not possible due to the low solubility of the complex; Elemental Anal. Calcd for C_8_H_12_ClN_3_O_2_Pt + HCl: C, 21.34; H, 3.13; N, 9.33. Found: C, 21.43; H, 3.02; N, 9.47; HR-MS: [M]^+^ exp, 413.0341, calcd, 413.0339.

#### (Ethylene,*N-trans*)(l-alaninato-N,O)chlorido(η^2^-(but-3-en-1-yl)-2-acetoxybenzoate)platinate(II) [PtCl(l-Ala)(ASA-Butene)] (**4**)

This complex was synthesized by exchanging the ethylene on complex **1a** with the olefin (but-3-en-1-yl)-2-acetoxybenzoate (ASA-butene, **6**), following the procedure described by Weninger et al.^4^ Absolute ethanol was degassed by 3 cycles of freeze–pump–thaw. A solution of ASA-butene (**6**, 0.36 mmol, 1.2 equiv) in about 2 mL of degassed absolute ethanol was prepared under an argon atmosphere and added dropwise to another solution, protected from light and under an argon atmosphere, of ZS (0.30 mmol, 1.0 equiv) in 8 mL of degassed absolute ethanol. The solution was heated to 48 °C and allowed to stir for 3 h. The reaction mixture was then filtered, and the solvent was removed, obtaining a yellow oil. Sonication of the oil with diethyl ether provided the desired product.

Yield: 54% as beige solid; purity: 97.00%; ^1^H NMR (400 MHz, (CD_3_)_2_CO): δ 8.05 (dd, ^3^*J* = 7.8 Hz, ^4^*J* = 1.8 Hz, 1H, ArH-6), 7.66 (ddd, ^3^*J* = 7.8 Hz, ^3^*J* = 8.1 Hz, ^4^*J* = 1.8 Hz, 1H, ArH-4), 7.40 (ddd, ^3^*J* = 7.8 Hz, ^3^*J* = 7.8 Hz, ^4^*J* = 1.2 Hz, 1H, ArH-5), 7.20 (dd, ^3^*J* = 8.1 Hz, ^4^*J* = 1.2 Hz, 1H, ArH-3), 6.24 (bd, *J*^2^ = 39.8 Hz, 1H, N–H), 5.45 (br s, 1H, N–H), 5.38–5.24 (m, 1H, –CH=CH_2_), 4.74–4.55 (m, 2H, –OCH_2_–), 4.54 (dt, *J*^3^ = 8.4, *J*^2^ = 1.8 Hz, 1H, =CH_α_H_β_), 4.47 (dt, *J*^3^ = 14.0, *J*^2^ = 1.8 Hz, 1H, =CH_α_H_β_), 3.97–3.75 (m, 1H, C_α_H), 2.61–2.47 (m, 1H, –CH_α_H_β_–), 2.30 (s, 3H, –OC(O) CH_3_), 2.28–2.17 (m, 1H, –CH_α_H_β_-), 1.51 (d, *J*^3^ = 7.1 Hz, 3H, –CH_3_); ^13^C NMR (101 MHz, (CD_3_)_2_CO): δ 184.2 (d, *J* = 1.9 Hz, –CH–C(O) O–), 169.8 (-OC(O) CH_3_), 165.0 (–C(O) O–CH_2_–), 151.7 (C2), 134.8 (C4), 132.36 (C6), 126.9 (C5), 124.9 (C3), 124.5 (C1), 95.1 (d, *J* = 28.8 Hz, –CH=CH_2_), 73.0 (d, *J* = 10.0 Hz, –CH=CH_2_), 64.3 (–OCH_2_–), 54.4 (d, *J* = 11.0 Hz, C_α_), 33.5 (d, *J* = 15.7 Hz, –CH_2_–CH=), 21.1 (–OC(O) CH_3_), 19.8 (d, *J* = 6.0 Hz, –CH_3_); ^195^Pt-NMR (86 MHz, (CD_3_)_2_CO): δ −2536. Elemental Anal. Calcd for C_16_H_20_ClNO_6_Pt: C, 34.76; H, 3.65; N, 2.53. Found: C, 34.66; H, 3.82; N, 2.48; HR-MS: [M + H]^+^ exp, 553.0692, calcd, 533.0701.

### Stability

#### Capillary Electrophoresis

A solution of sodium tetraborate (final borate concentration of 50 mM) and sodium dodecyl sulfate (100 mM) was used as a background electrolyte (BGE). The pH of the BGE was adjusted to 9.3 by titration with 1 M NaOH. Every new capillary was rinsed with 1 M NaOH (45 min), water (45 min), and BGE (45 min) before the first use. The capillary was kept on a constant temperature of 25 °C, whereas the sample carousel was temperature-controlled at 37 °C. Sample injection was performed in hydrodynamic mode, applying a pressure of 50 mbar for 2 s on the inlet vial. The separation voltage was set at +20 kV. If not stated differently, a wavelength of 195 nm was employed for the DAD detector. The analytic window was evaluated by using dodecaphenone as a micellar marker and methanol (HPLC grade) as an electroosmotic flow (EOF) marker. The run time required for the analysis was estimated to be 25 min. The capillary was flushed before every run with 0.1 M NaOH (3 min), water (3 min), and BGE (5 min). Every value is calculated from at least three independent measurements. The half-lives were presented as the mean ± standard deviation. All of the samples, buffers, and washing solutions were membrane-filtered (0.22 μm pore size). All samples employed for stability experiments were prepared using benzoic acid as an internal standard (IS).

### Characterization

#### IR Multiple-photon Dissociation Spectroscopy

IRMPD spectra were obtained at the Free Electron Laser for IR eXperiments (FELIX) facility (Nijmegen, The Netherlands) employing a commercial 3D quadrupole ion trap mass spectrometer (Bruker amaZon speed ETD) modified to permit for optical access to the trapped ions.^47^ Samples were directly infused at a 120 μL h^–1^ rate and ionized in positive ion mode using an ESI source. The ions of interest were mass-selected and irradiated by a single IR pulse from the IR free electron laser. The FEL was operated at a 10 Hz repetition rate with a pulse energy of 40–100 mJ in the frequency range of 650–1900 cm^–1^ with a step size of 5 cm^–1^. At each step, 6 replicate mass spectra were averaged. Spectra were recorded at several levels of laser pulse energy attenuation in order to prevent excessive depletion of the parent ions (saturation) and minimize the formation of fragment ions below the low mass cutoff of the MS.^48^ To produce the IRMPD spectrum, the photofragmentation yield *R* (*R* = −ln[*I*_P_/(*I*_P_ + Σ*I*_F_)], where *I*_P_ and *I*_F_ are the abundances of the parent ion and of a fragment ion, respectively) was plotted as a function of the wavenumber.^49^ Finally, the yield was linearly corrected for the frequency-dependent variations in laser pulse energy.^50^

#### Computational Methods

Guess geometries were optimized using the DFT functional B3LYP-D3, and the 6-311++G(d,p) basis set for all atoms but platinum, for which the LanL2TZ basis set was employed.^27^ Harmonic vibrational frequencies were computed at the same theory level to obtain IR spectra and thermodynamic corrections to the electronic energies. In addition, single-point energy calculations at the M06-2X/def2TZVP level were performed to evaluate the influence of a higher percentage of HF exchange on the relative energies of the isomers. B3LYP-D3 thermodynamic corrections were used to obtain the relative enthalpies and Gibbs free energies at the M06-2X level. All DFT calculations were performed using Gaussian 09 rev. D.01.^51^ To plot the calculated spectra, harmonic frequencies were scaled by 0.97 based on their good agreement with the IRMPD spectra.^27,29^ Calculated linear IR spectra were convoluted with a Gaussian profile of 20 cm^–1^ (fwhm).

#### X-ray Crystallography

For single-crystal structure analysis, crystals were measured into a stream of cold N_2_ (173 K) inside a Bruker D8 Quest diffractometer (Photon III C14). The instrument was equipped with an Incoatec Microfocus source generator (multi layered optics monochromatized Mo Kα radiation, λ = 71.073 pm). Multiscan absorption corrections were applied with the program SADABS-2014/5. SHELXT and SHELXL programs^52,53^ were used for structure solution and refinement. Hydrogen atoms at ethylene were found and refined with isotropic displacement parameters and bond restraints (95 pm). Additional details of the crystal structure investigation can be obtained from the Cambridge Crystallographic Data Centre (CCDC). The supplementary crystallographic data of **1a** and **1b** were deposited as CCDC numbers 2262096 and 2262097, respectively. The authors will release the atomic coordinates upon article publication.

#### Biological Testing

##### Cell Lines

The ovarian carcinoma cell line A2780cis was kindly provided by the Department of Gynecology, Medical University Innsbruck.

The breast cancer cell line MCF-7 and the colon carcinoma cell line HT-29 were purchased from DSMZ, German Collection of Microorganisms and Cell Cultures, Braunschweig, Germany. The breast cancer cell line MDA-MB-231 was kindly provided by the Department of Hematology, Medical University of Innsbruck.

The cell lines A2780cis and MDA-MB-231 were cultivated in RPMI 1640 without phenol red (PAN Biotech, Aidenbach, Germany), supplemented with l-glutamine (2 mM), 100 μg/mL penicillin, 100 μg/mL streptomycin, and FCS (10%) (all from Invitrogen Corporation, Gibco, Paisley, Scotland) at 37 °C in a 5% CO_2_/95% air atmosphere, and passaged twice per week. To maintain resistance, A2780cis cells were incubated every second week with cisplatin (1 μM). The cell lines MCF-7 and HT-29 were grown in DMEM without phenol red (PAN Biotech, Aidenbach, Germany), containing l-glutamine, 100 μg/mL penicillin, 100 μg/mL streptomycin, sodium pyruvate (100 mM) (PAN Biotech), and FCS (10%) under the same conditions as the other cell lines.

##### Analysis of Cell Growth Inhibition

The exponentially growing cell lines were seeded at a density of 2000 cells/well for MCF-7 cells, 4000 cells/well for HT-29 cells and MDA-MB-231 cells, and 8000 cells/well for A2780cis cells, respectively, into clear flat-bottom 96-well plates in triplicates. Following 24 h of incubation for adherence at 37 °C in a humidified atmosphere (5% CO_2_/95% air), the compounds were added to reach the desired concentrations between 10 and 100 μM, respectively. All stock solutions were prepared in dimethylformamide at a concentration of 100 mM and were then diluted with the respective cell-culture medium to the appropriate concentrations. After another 72 h of incubation, the cellular metabolic activity was measured by employing a MTT assay. Hereby, the yellow tetrazolium salt is converted to a purple formazan salt by the functioning mitochondria. These purple crystals are then dissolved in DMSO and can be quantified via absorption measurements at 570 and 420 nm. The optical density of the particular medium was subtracted in order to exclude the unspecific staining caused by the FCS-containing medium. The values were calculated with Excel 2019 (Microsoft, Redmond, WA, USA) using nonlinear regression and the decal logarithm of the inhibitor versus variable slope equation, while the top constraint was set to 100%.

##### Determination of the COX-Inhibition

Inhibition of the isolated human recombinant COX-1 and COX-2 isoenzymes by the platinum complexes (10 and 25 μM) was evaluated using an enzyme immunoassay (EIA) (COX Inhibitor Screening Assay, Cayman Chemicals, Ann Arbor, Mi, USA) following the manufacturer’s protocol. The incubation time of the compounds with the respective isoenzymes was exactly 2 min. The results are presented as the mean ± SD of three independent experiments with two replicates of each experiment. The untreated control was set at 0% inhibition of the COX activity.

## Supporting Information Available

NMR characterization spectra, electropherograms, crystal data and structure refinements, NMR experiments setup, IRMPD data, and supplementary biological data (PDF)
Molecular formula strings (CSV)

## Abbreviations

ASA: acetylsalicylic acid
BGE: background electrolyte
CCDC: Cambridge Crystallographic Data Centre
CE: capillary electrophoresis
DAD: diode array detector
DMEM: Dulbecco’s modified Eagle medium
EIA: enzyme immunoassay
EOF: electroosmotic flow
FAP: familial adenomatous polyposis
FCS: fetal calf serum
FEL: free electron laser
IRMPD: infrared multi-photon dissociation
IS: internal standard
MEKC: Micellar electrokinetic chromatography
MTT: 3-(4,5-dimethylthiazol-2-yl)-2,5-diphenyltetrazoliumbromid
ORTEP: Oak Ridge Thermal-Ellipsoid Plot
PG: prostaglandin
*P*_ow_: partition coefficient between *n*-octanol and water
*R*: photofragmentation yield
SD: standard deviation
τ_1/2_: half-life time
TMG: trimethylglycine
ZS: Zeise’s salt
