# Supplementary material for: Amino Acids as Chelating Ligands for Platinum: Enhanced Stability in an Aqueous Environment Promoted by Biocompatible Molecules
Source: J Med Chem. 2023 Nov 8;66(22):15256–68. doi: 10.1021/acs.jmedchem.3c01340 (PMC10683014; doi:10.1021/acs.jmedchem.3c01340)
Supplement: Supplementary file 1 — jm3c01340_si_001.pdf [file jm3c01340_si_001.pdf]

SUPPORTING INFORMATION

Amino acids as chelating ligands for platinum: enhanced stability in aqueous environment promoted by biocompatible molecules

Andrea Cucchiaro[1], Amelie Scherfler[1], Davide Corinti[2], Giel Berden[3], Jos Oomens[3], Klaus Wurst[4], Ronald Gust[1#], Maria Elisa Crestoni[2], Brigitte Kircher[5,6], Monika Cziferszky[1,*]

1. Institute of Pharmacy, Pharmaceutical Chemistry, Center for Molecular Biosciences Innsbruck, University of Innsbruck, Innrain 80-82, A-6020 Innsbruck, Austria
2. Dipartimento di Chimica e Tecnologie del Farmaco, Università di Roma "La Sapienza", P. le A. Moro 5, I-00185 Roma, Italy
3. Radboud University, Institute for Molecules and Materials, FELIX Laboratory, Toernooiveld 7, 6525ED Nijmegen, the Netherlands
4. Institute of General, Inorganic and Theoretical Chemistry, University of Innsbruck, CCB—Centrum for Chemistry and Biomedicine, Innrain 80-82, 6020 Innsbruck, Austria
5. Tyrolean Cancer Research Institute, Innrain 66, 6020 Innsbruck, Austria
6. Immunobiology and Stem Cell Laboratory, Department of Internal Medicine V (Hematology and Oncology), Medical University of Innsbruck, Anichstraße 35, 6020 Innsbruck, Austria

Correspondence: monika.cziferszky@uibk.ac.at

Index

Molecular Formula Strings 2
Characterization of **1a** 2
Characterization of **2b** 6
Characterization of **3** 9
Characterization of **4** 12
DFT optimized structures for **1a**, **1b**, **2a**, and **2b** 15
Electropherograms of **1a** - **4** 16
Crystal data and structure refinements for complex **1a** 18
Crystal data and structure refinements for complex **1b** 27
NMR study for the synthesis of complex **1** 33
NMR investigation of the stability of complex **2** in an aqueous solution 34
NMR study for the dissolution of ZS in a highly concentrated TMG solution 37
Biological data 38
Statistical analysis 38
References 40

Molecular Formula Strings

Compound	SMILES
1a	Cl[Pt]12(OC([C@H](C)[NH2]2)=O)[CH2]=[CH2]1
2b	Cl[Pt]1(OC2=O)([NH2]CC2)[CH2]=[CH2]1
3	Cl[Pt]12([NH2][C@@H](CC3=CNC=[N]32)C(O)=O)[CH2]=[CH2]1
4	Cl[Pt]12(OC([C@H](C)[NH2]2)=O)[CH2]=[CH]1CCOC(C3=CC=CC=C3OC(C)=O)=O
5	Cl[Pt]1(Cl)(Cl)[CH2]=[CH]1CCOC(C2=CC=CC=C2OC(C)=O)=O
6	O=C(C1=CC=CC=C1OC(C)=O)OCCC=C

Characterization of **1a**

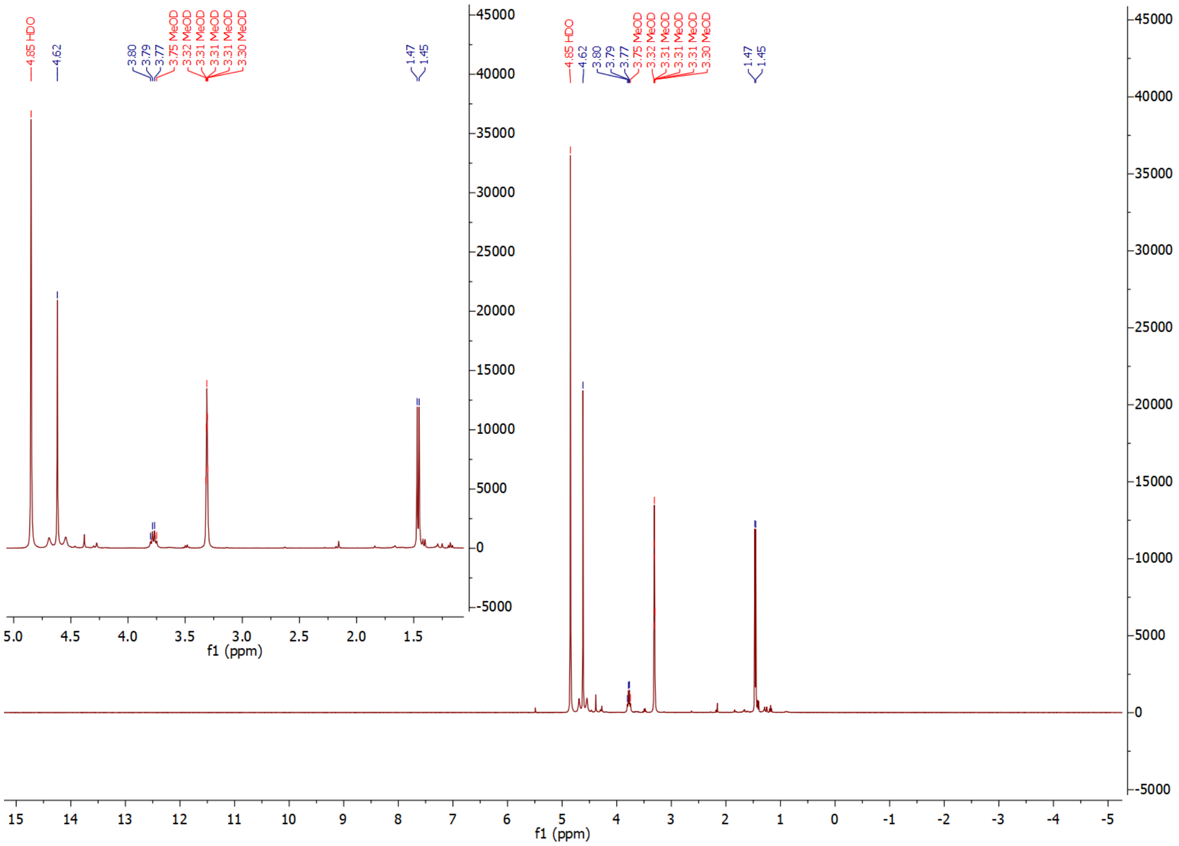

*Figure S1 ^{1}H-NMR spectrum of complex **1a***

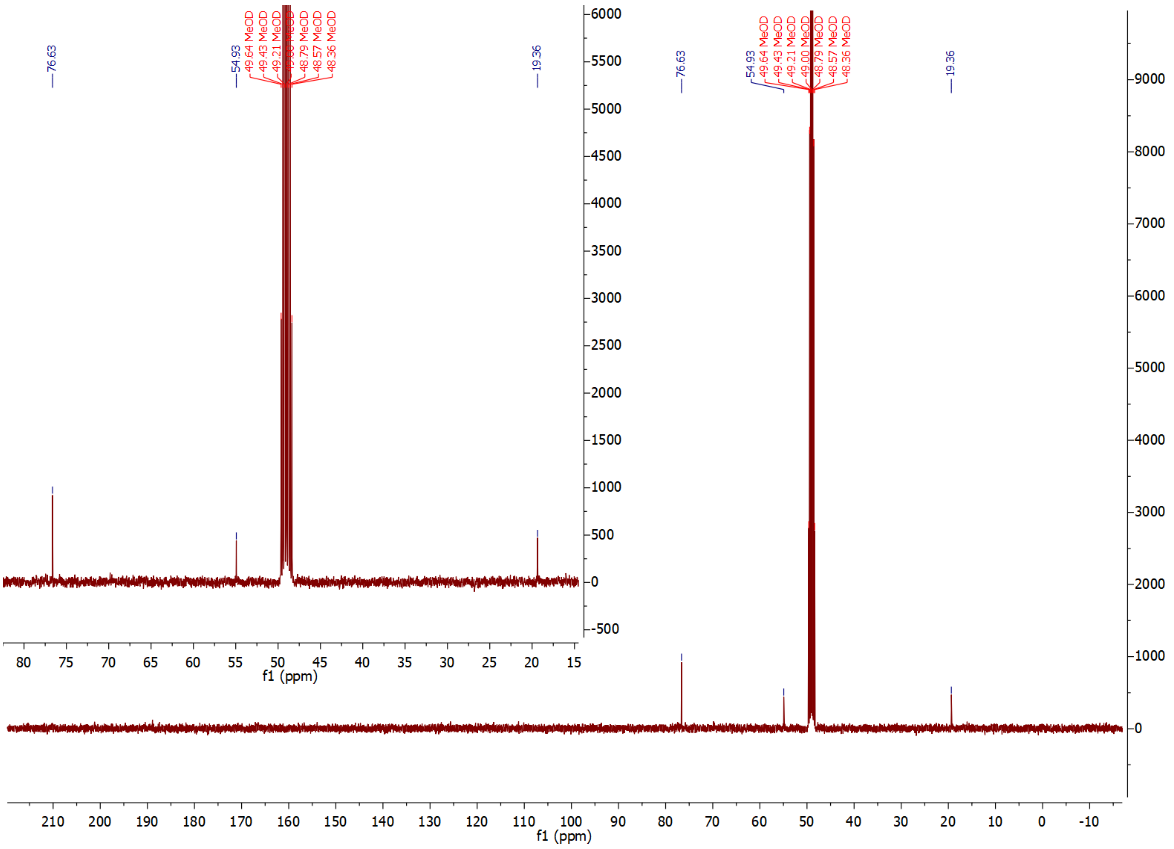

*Figure S2 ^{13}C-NMR spectrum of complex **1a***

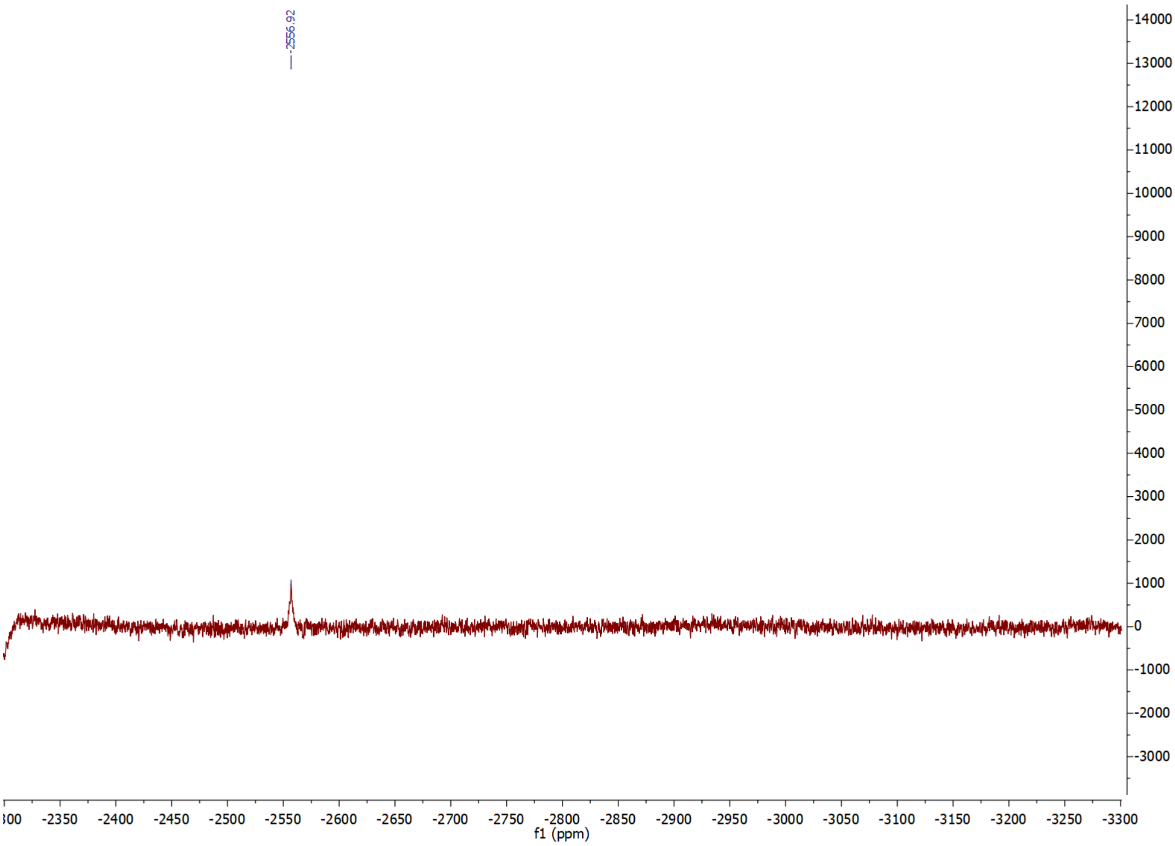

*Figure S3 ^{195}Pt-NMR spectrum of complex **1a***

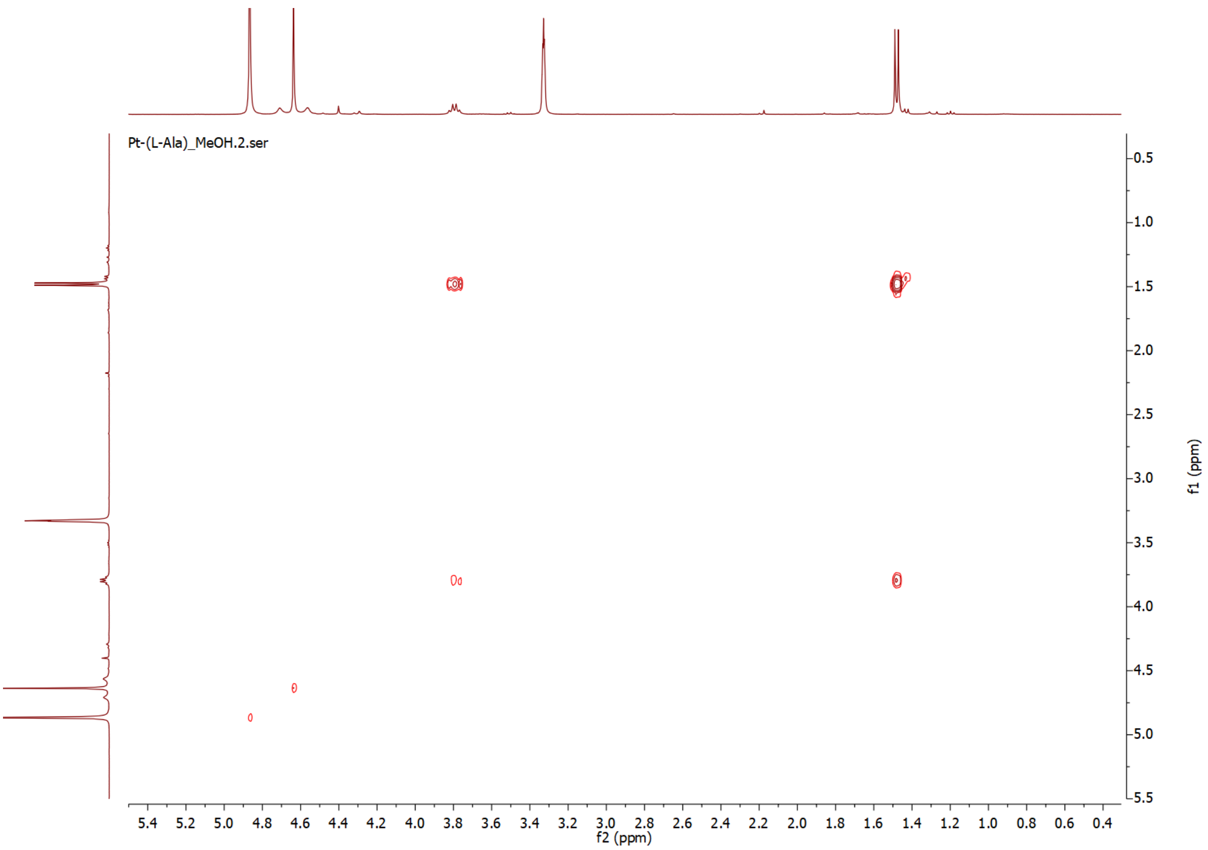

*Figure S4 [^{1}H, ^{1}H]-COSY 2D-NMR spectrum of complex **1a***

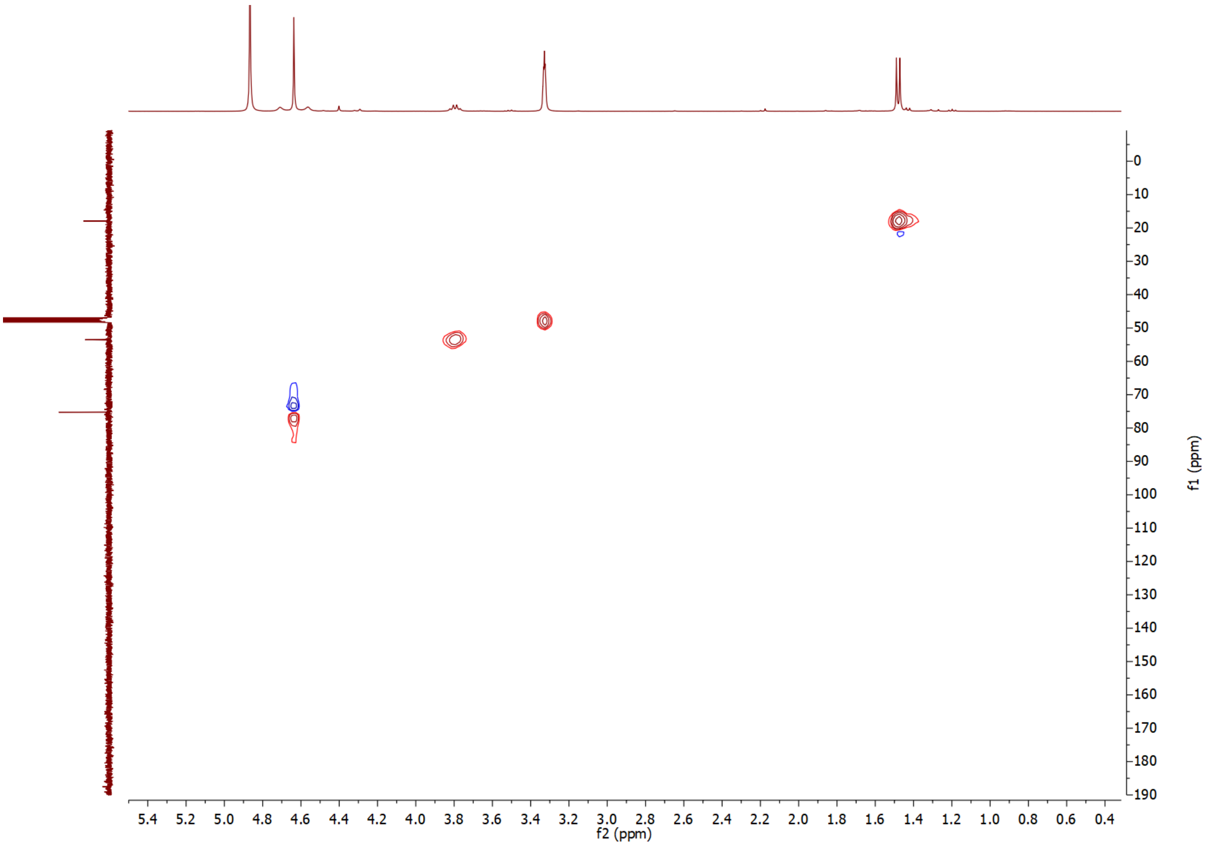

*Figure S5 [^{1}H, ^{13}C]-HSQC 2D-NMR spectrum of complex **1a***

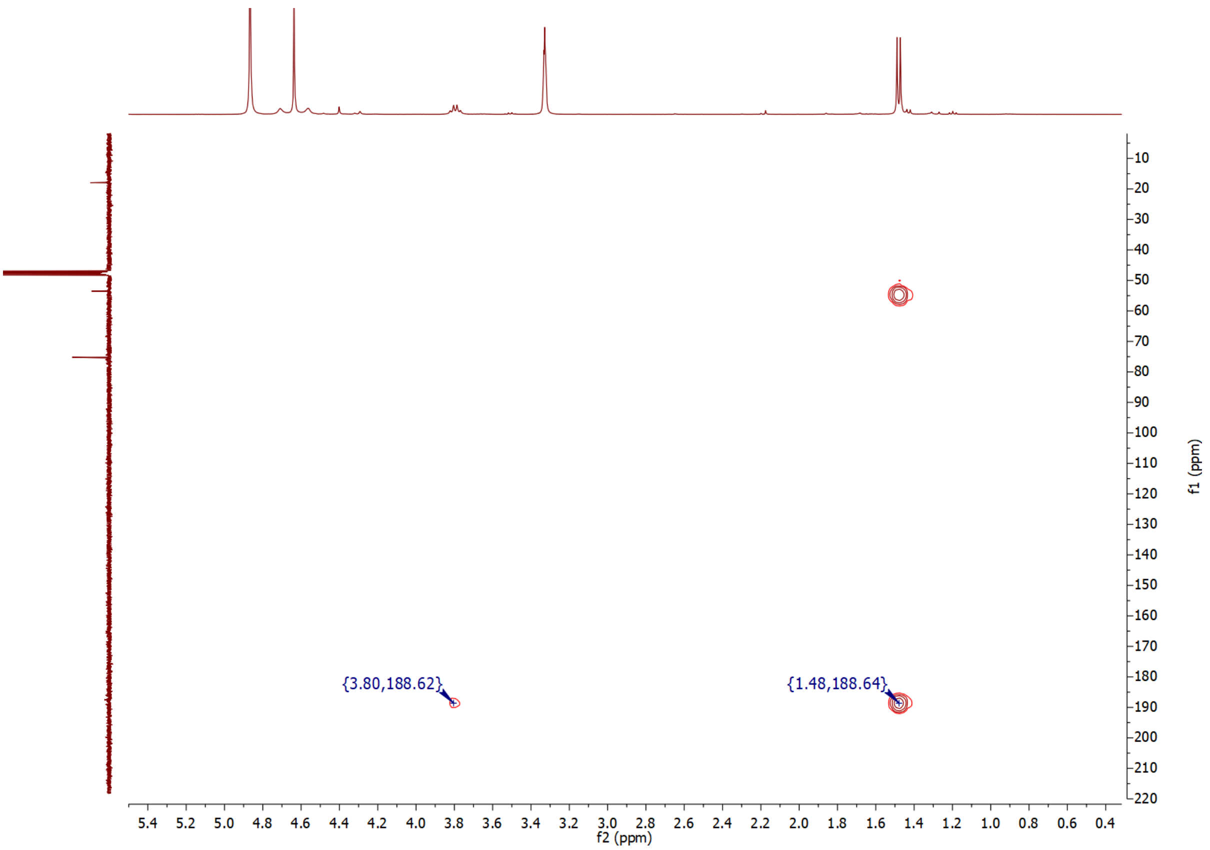

*Figure S6 [^{1}H,^{13}C]-HMBC 2D-NMR spectrum of complex **1a***

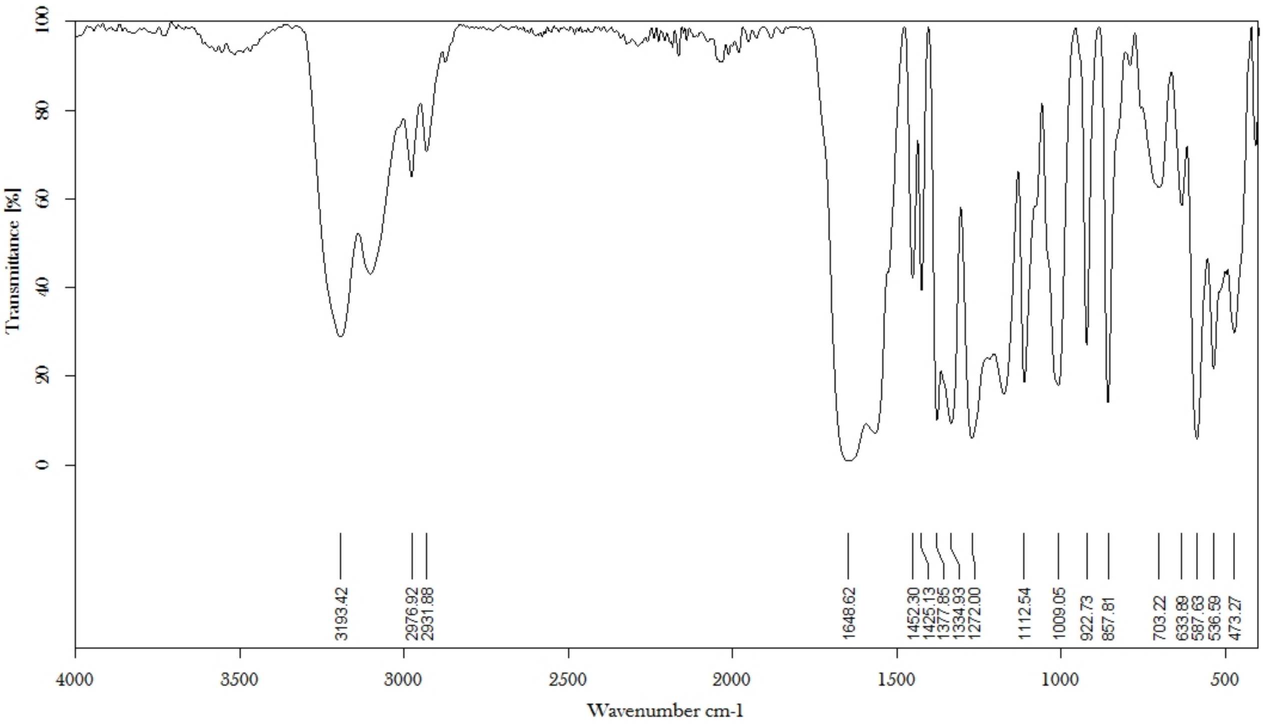

*Figure S7 IR spectrum of complex **1a***

Characterization of **2b**

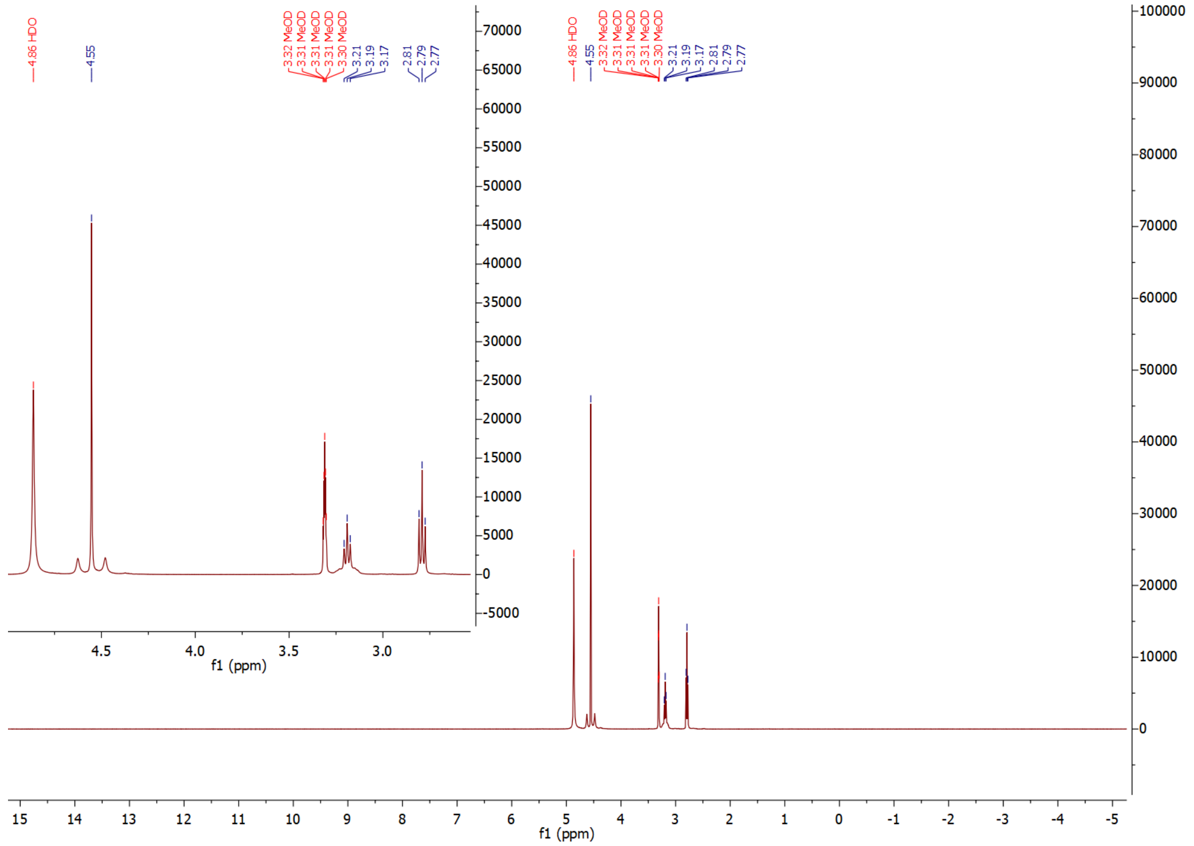

*Figure S8 ¹H-NMR spectrum of complex **2b***

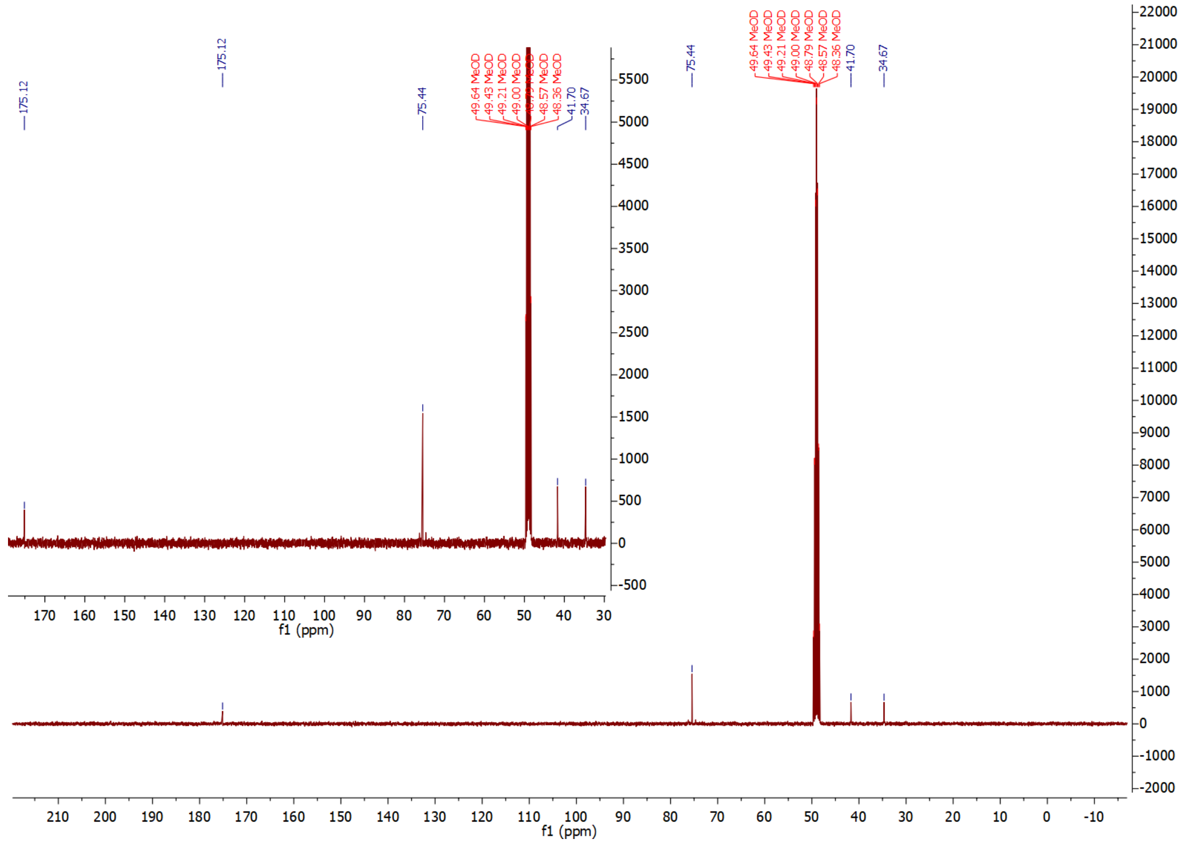

*Figure S9 ¹³C-NMR spectrum of complex **2b***

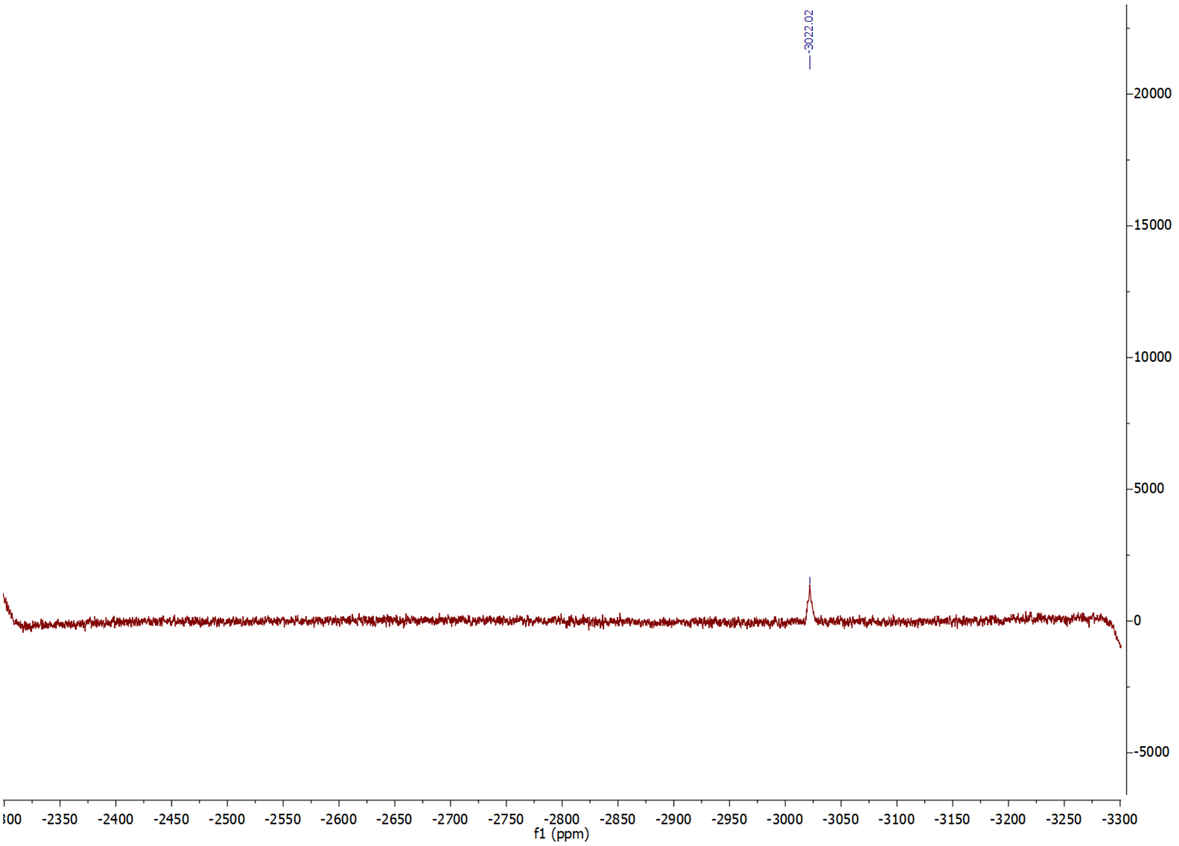

*Figure S10 ^{195}Pt-NMR spectrum of complex **2b***

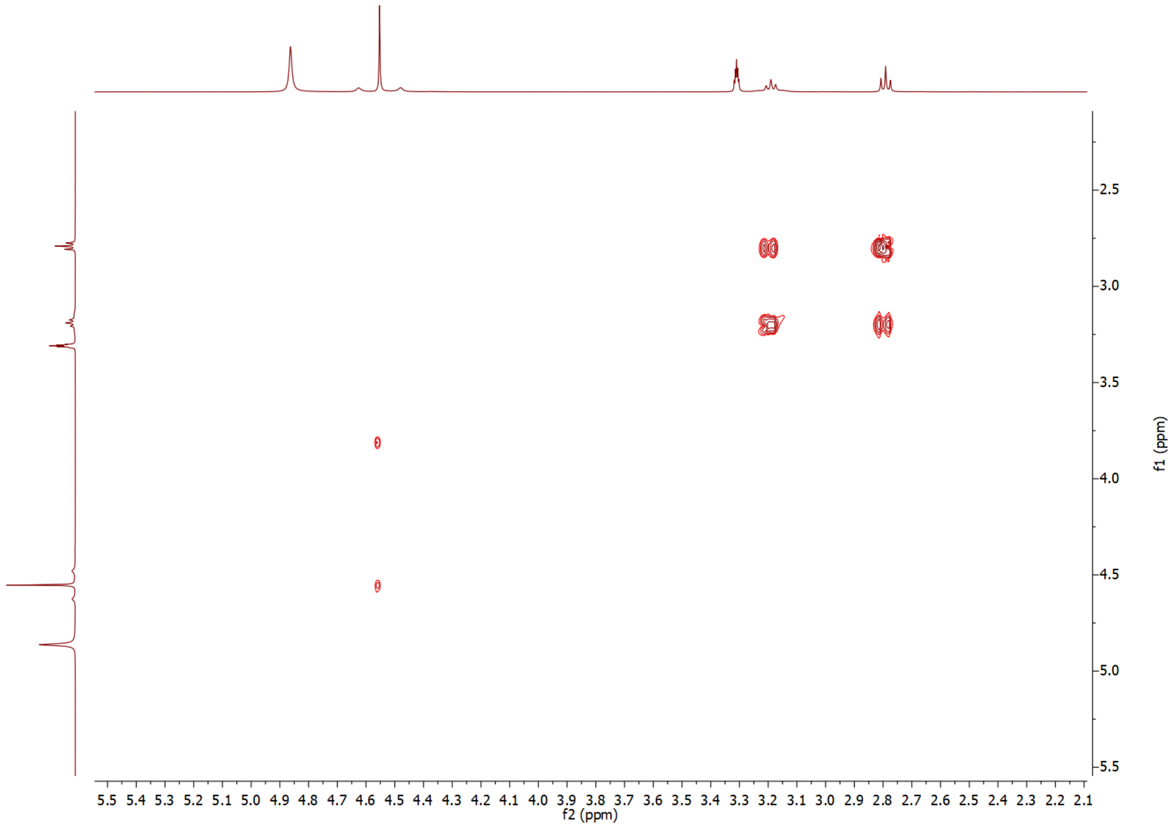

*Figure S11 [^{1}H, ^{1}H]-COSY 2D-NMR spectrum of complex **2b***

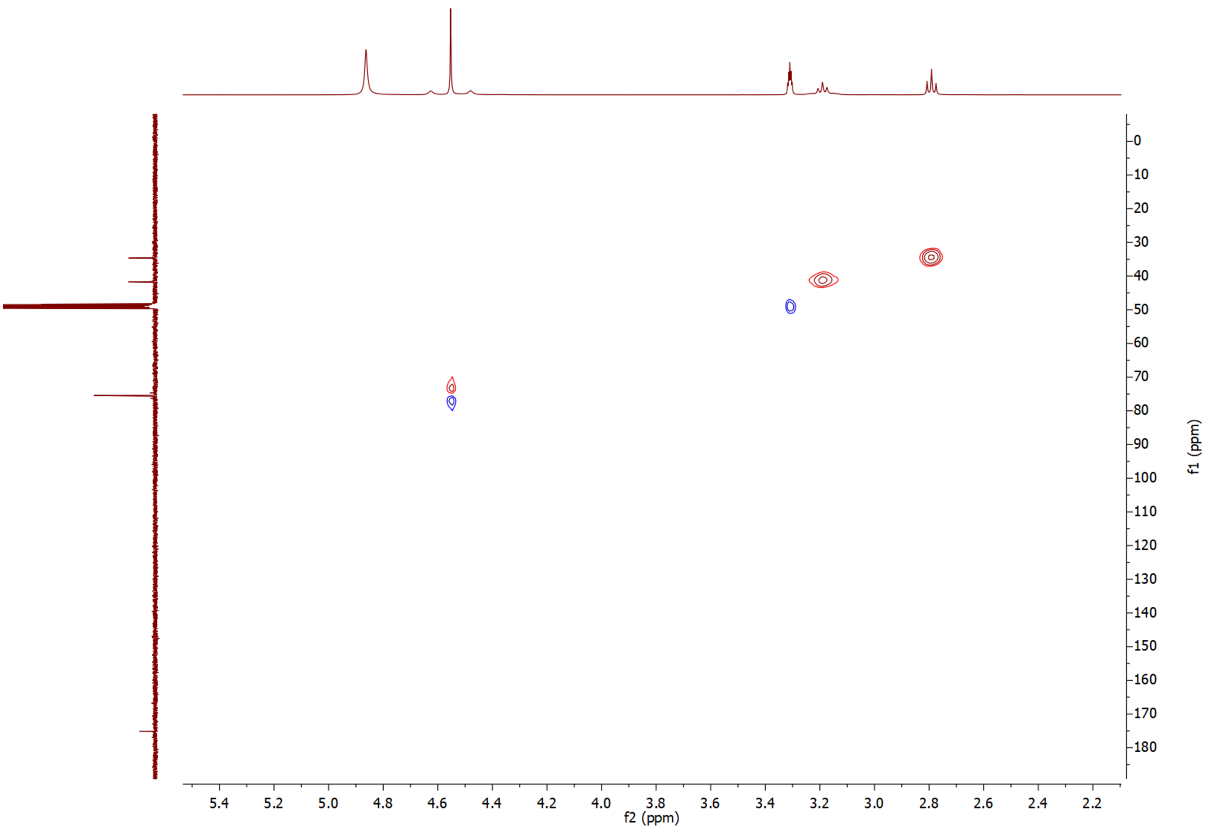

Figure S12 [^{1}H, ^{13}C]-HSQC 2D-NMR spectrum of complex ***2b***

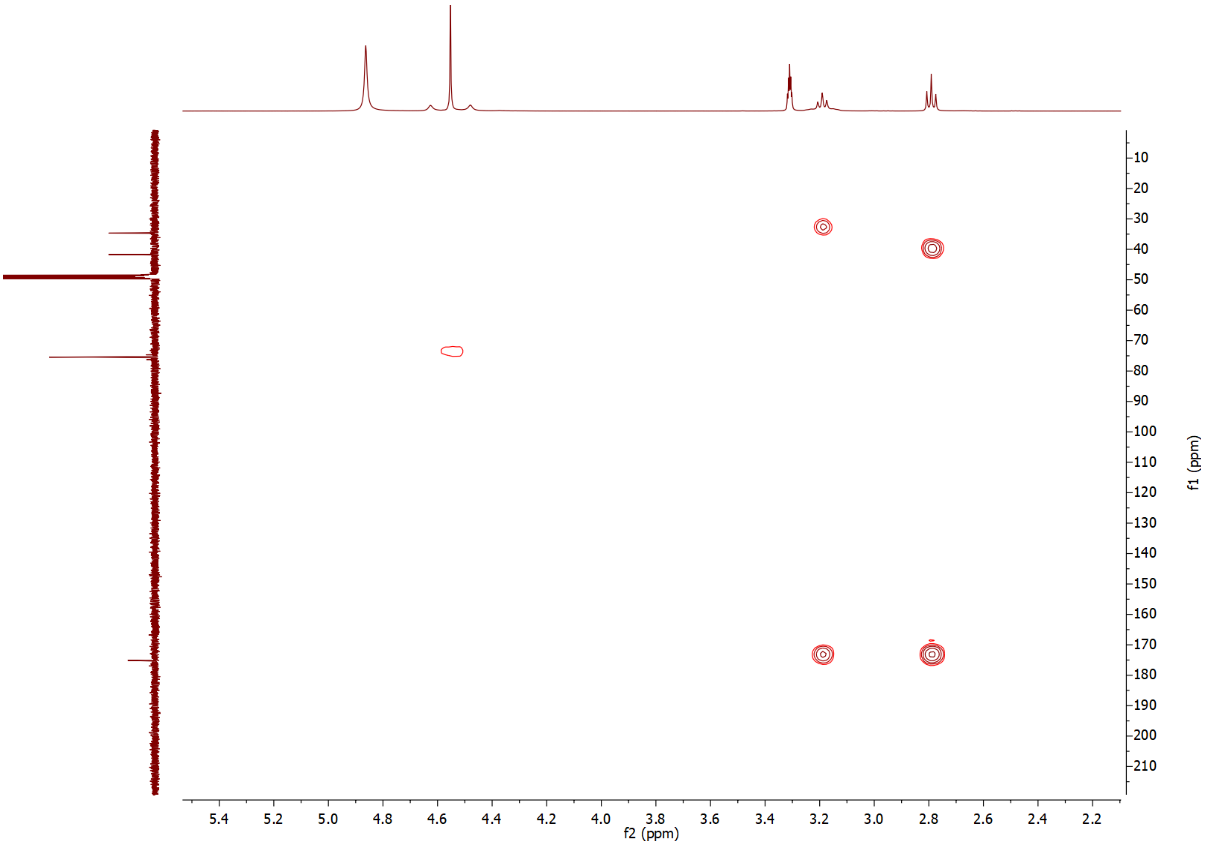

Figure S13 [^{1}H, ^{13}C]-HMBC 2D-NMR spectrum of complex ***2b***

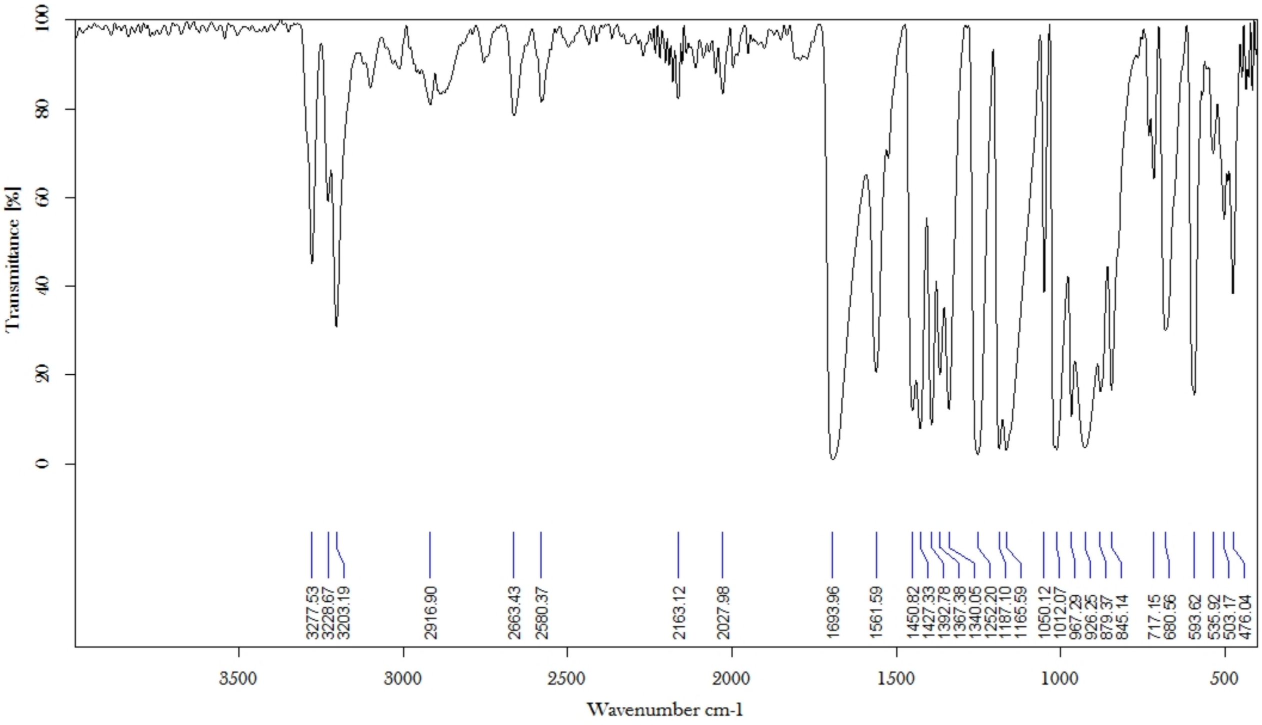

*Figure S14 IR spectrum of complex **2b***

Characterization of **3**

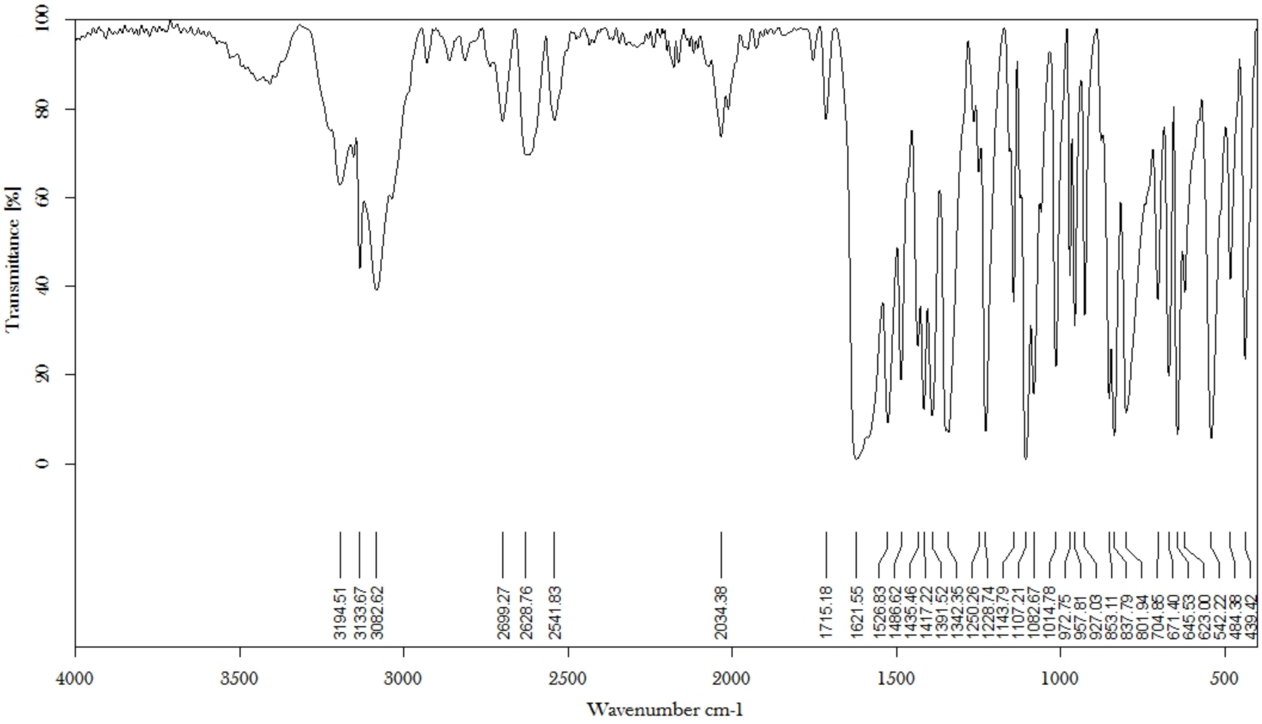

*Figure S15 IR spectrum of complex **3***

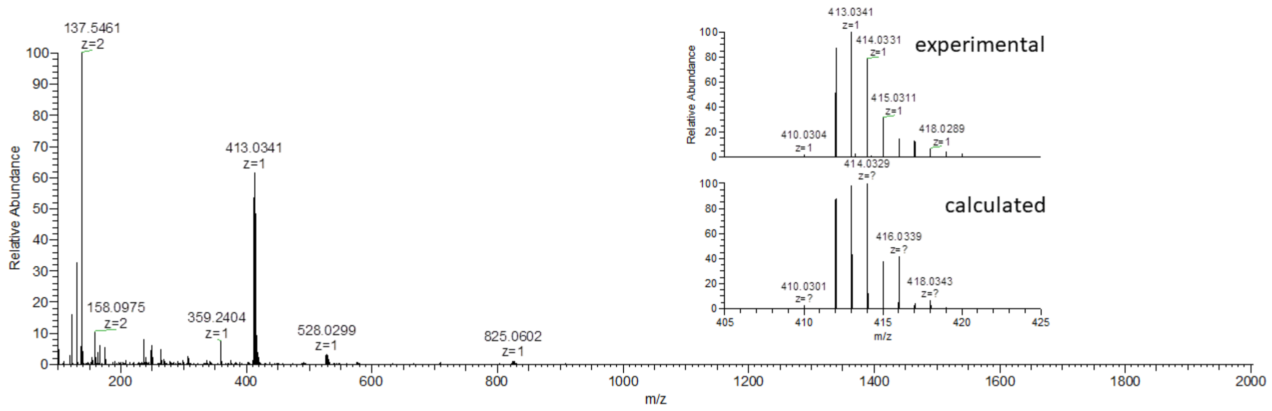

*Figure S16 HR-ESI-MS spectrum of complex **3** recorded in positive mode*

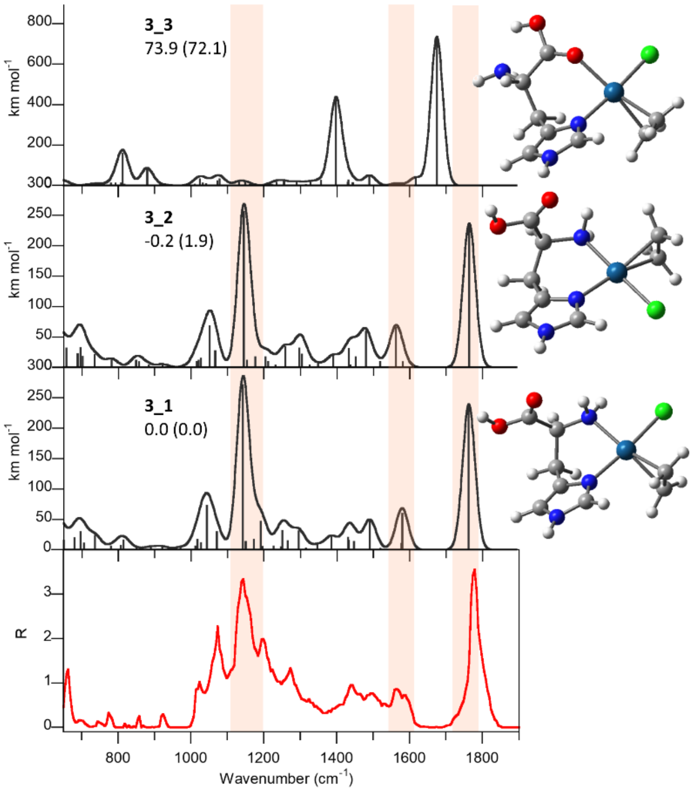

*Figure S17 IRMPD spectra of **3** (red profile), compared with calculated linear IR spectra of the structural isomers **3_1**, **3_2** and **3_3** (black profiles) together with their optimized geometries at the B3LYP level on the right. Relative enthalpies (free energies) at 298 K calculated at the M06-2X level are reported in kJ mol^{-1}.*

Figure S17 shows the IRMPD spectrum of the cluster of signals at *m/z* 413, which is compatible with the mass and the isotopic pattern of the synthetized complex **3**. A few vibrational modes are worth mentioning. The experimental band at 1775 cm^{-1} can be interpreted by the calculated CO stretching mode of **3_1** and **3_2** at 1762 and 1763 cm^{-1}, respectively, while the same vibration is calculated for **3_3** at 1676 cm^{-1}, thus red-shifted due to the interaction of the carboxylic CO with Pt. In addition, the most intense band of the IRMPD spectrum at 1143 cm^{-1} is correctly reproduced only by the computed OH bending mode of **3_1** and **3_2** (1142 and 1145 cm^{-1}, respectively). Further spectroscopic evidence for the binding of the amino nitrogen to platinum is the neat experimental band at 1570 cm^{-1}, which can be assigned to the NH_2 scissoring mode of the amino group when bound to platinum. In contrast, **3_3** presents this vibrational mode at 1617 cm^{-1}. In summary, spectroscopy agrees with the thermodynamic data in assessing the formation of a N,N-bound chelate of histidine to the platinum complex, as in **3_1** and **3_2**, while there is no evidence of the formation of an O-

bound species through the carboxylate (**3_3**). Small discrepancies between the experimental and calculated intensities can be attributed either to deficiencies in the theoretical approach or to the non-linear character of the multiple-photon absorption process as extensively described in literature.[1–5]

*Table S1 IRMPD features and theoretical IR frequencies (cm^{-1}), infrared absorption intensities (km mol^{-1}) in parenthesis, of **3_1** and **3_2** and vibrational normal modes obtained by DFT calculations at the B3LYP level.*

**Experimental**	**Theoretical**		
**3**	**3_1**	**3_2**	**Assignment**
1775	1762 (240)	1763 (238)	CO stretching
1570	1579 (59)	1561 (65)	NH_2 scissoring
1495	1490 (49)	1480 (58)	CH(imi) ip bending
1435	1431 (20)	1433 (30)	CH_2 scissoring
1390	1385 (22)	1390 (20)	CH bending
1272	1295 (30)	1297 (31)	NH_2 twisting
1198	1191 (46)	1257 (33)	CH bending
		1203 (17)	
1143	1142 (271)	1145 (256)	OH bending
1070	1043 (72)	1051 (68)	CN stretching
1021	1017 (16)	1027 (14)	CH_2(eth) wagging
858	815 (16)		CH_2(eth) rocking
778		781 (14)	CH(imi) oop bending
712	732 (23)	736 (21)	CH(imi) oop bending
698	696 (29)	696 (31)	CH_2(eth) twisting
660	659 (31)	659 (31)	NH(imi) oop bending

Characterization of **4**

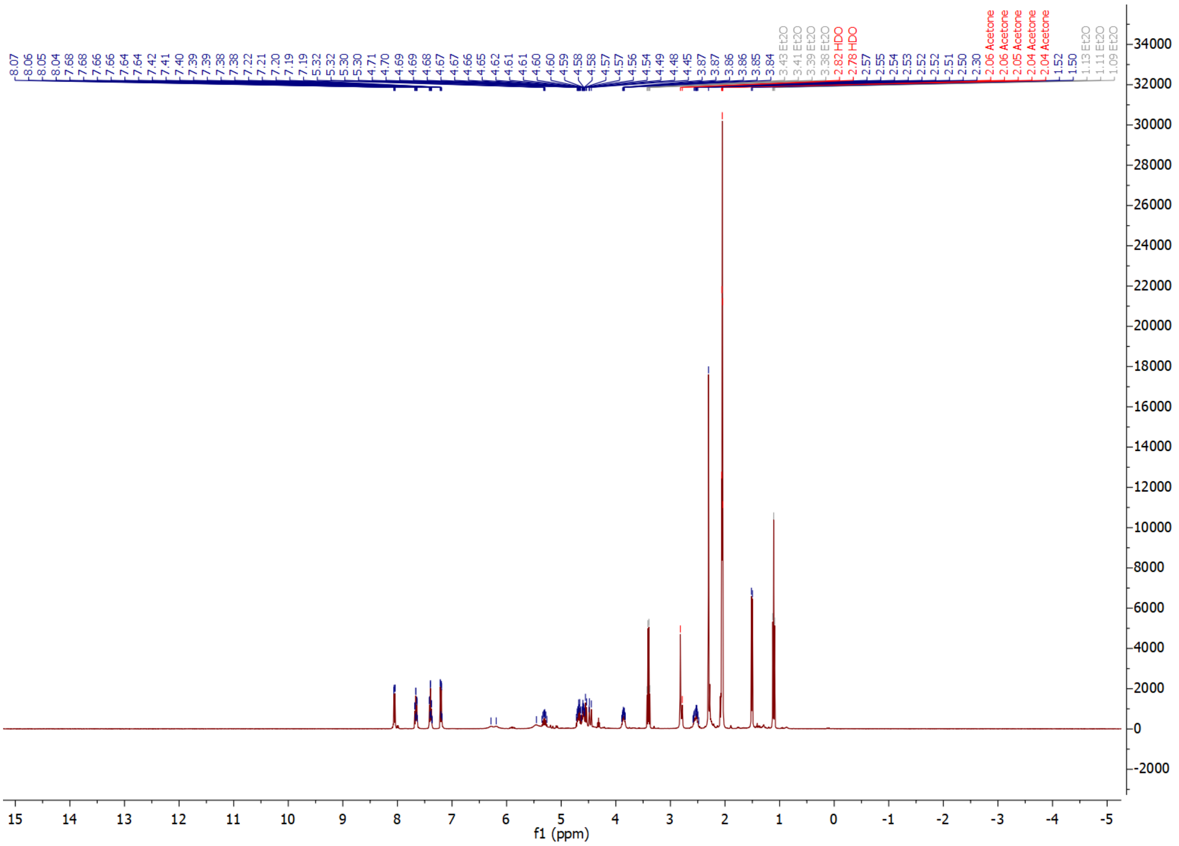

*Figure S18 ^{1}H-NMR spectrum of complex **4***

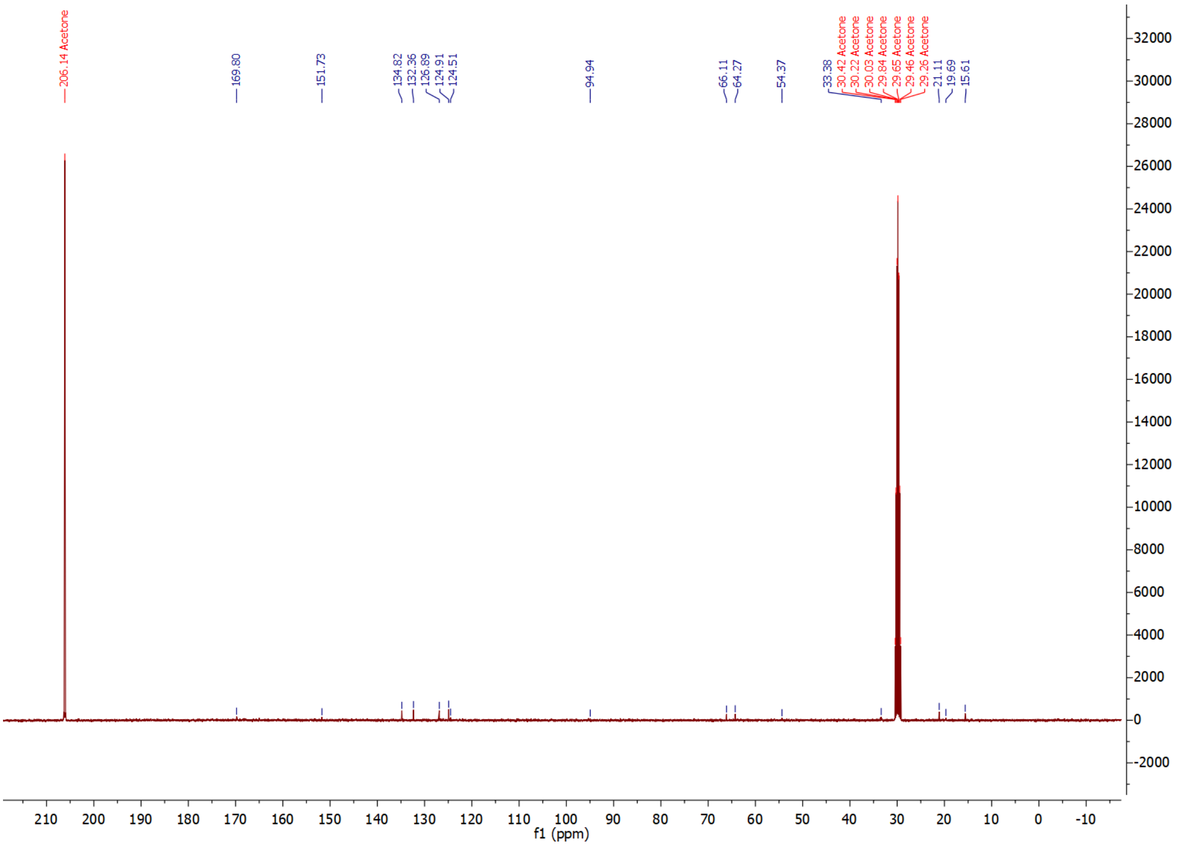

*Figure S19 ^{13}C-NMR spectrum of complex **4***

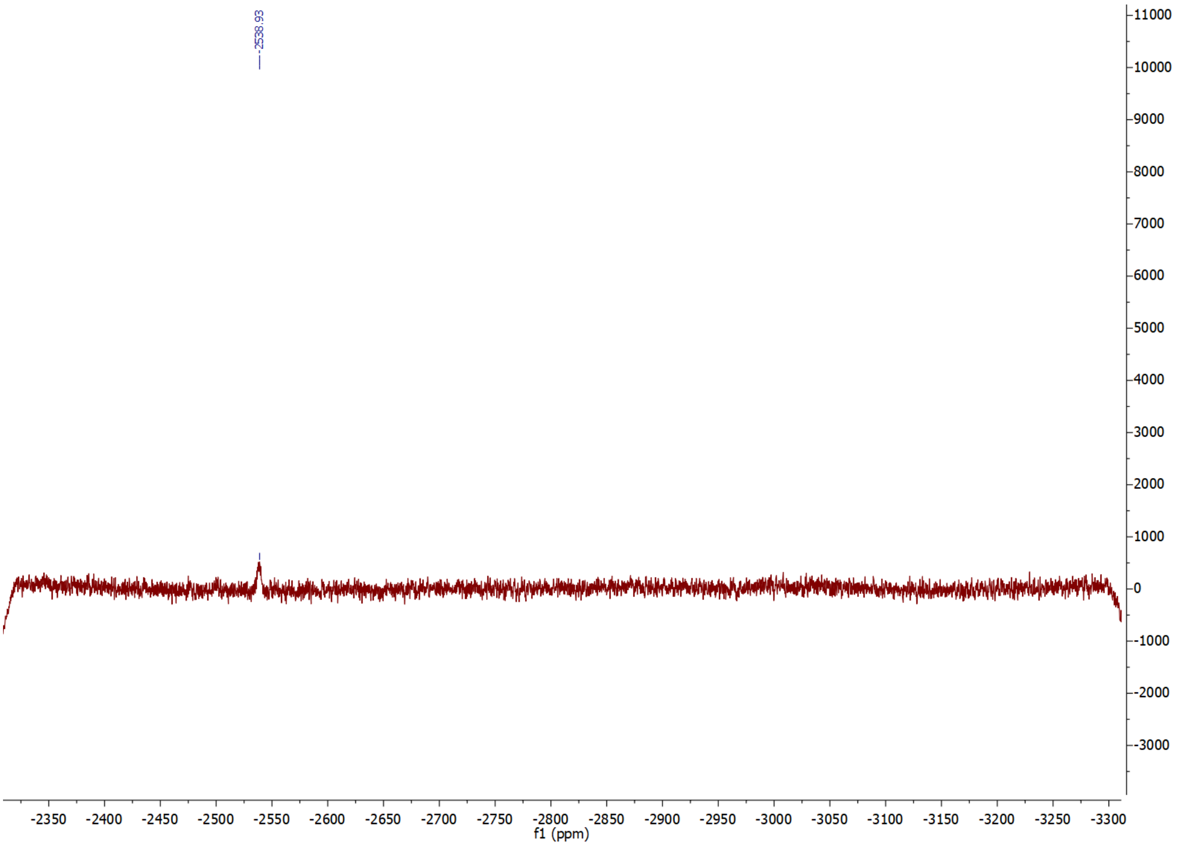

*Figure S20 ^{195}Pt-NMR spectrum of complex **4***

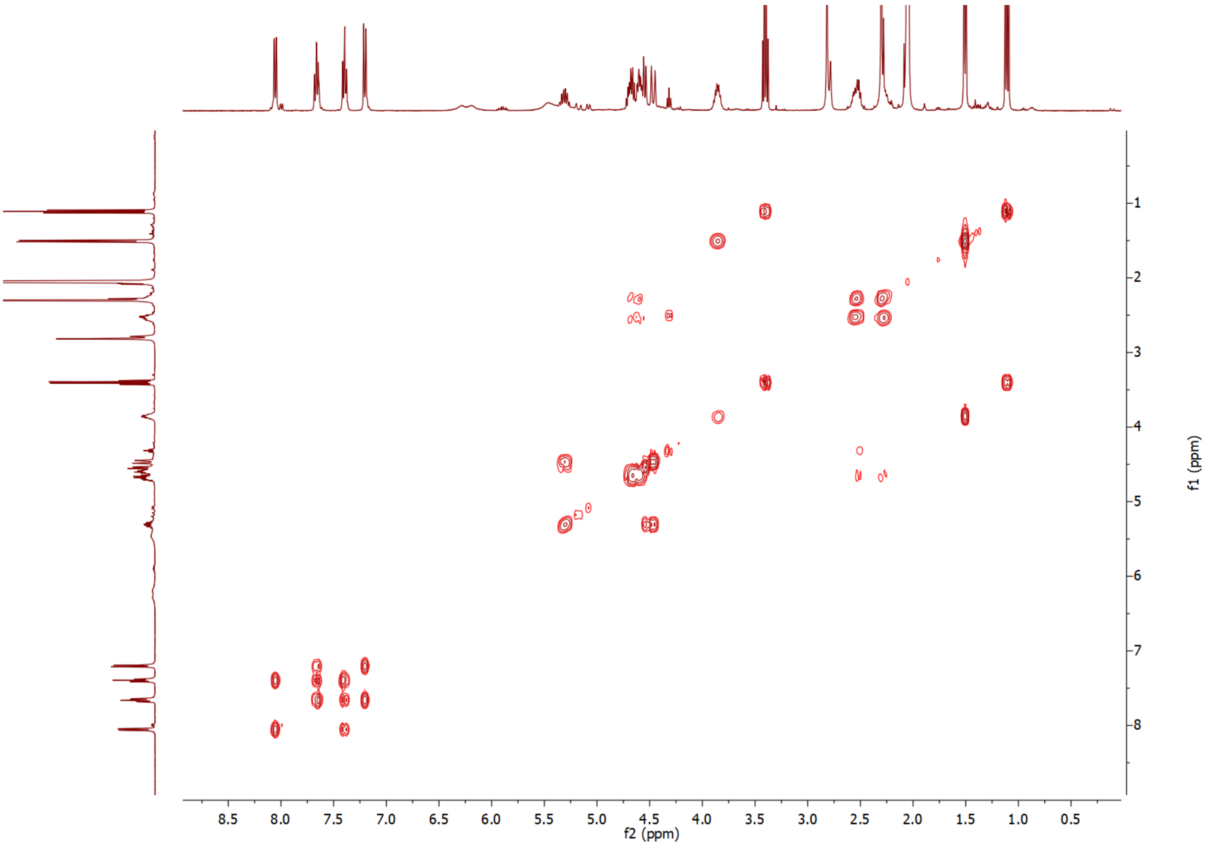

*Figure S21 [^{1}H, ^{1}H]-COSY 2D-NMR spectrum of complex **4***

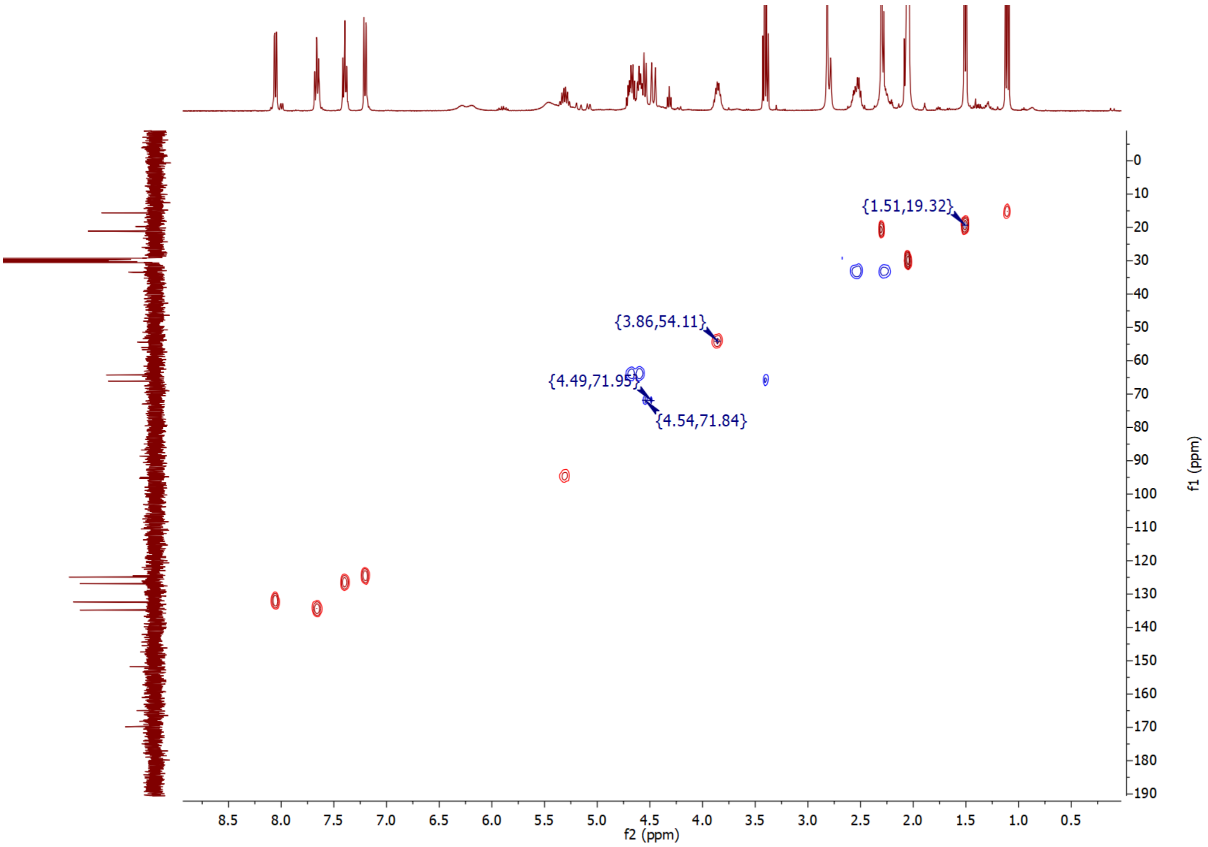

Figure S22 [^{1}H, ^{13}C]-HSQC 2D-NMR spectrum of complex ***4***

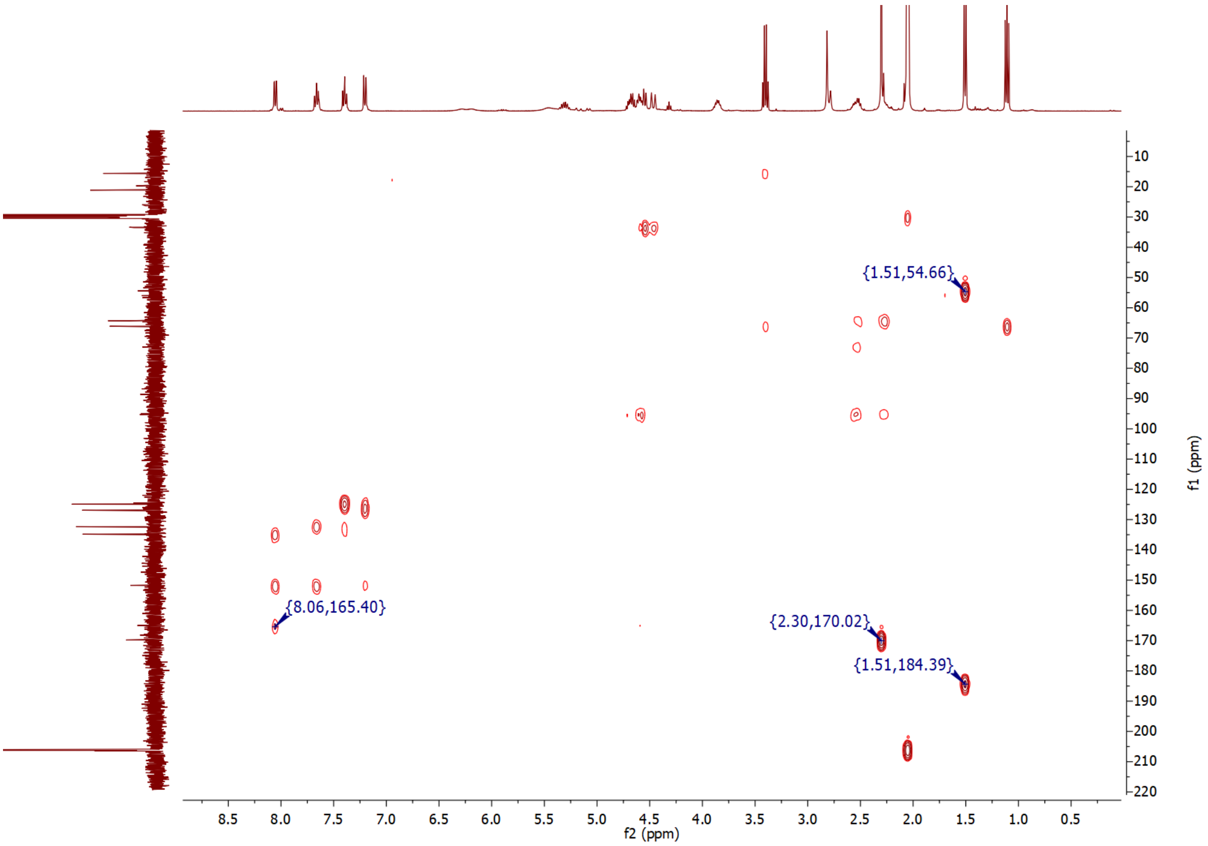

Figure S23 [^{1}H, ^{13}C]-HMBC 2D-NMR spectrum of complex ***4***

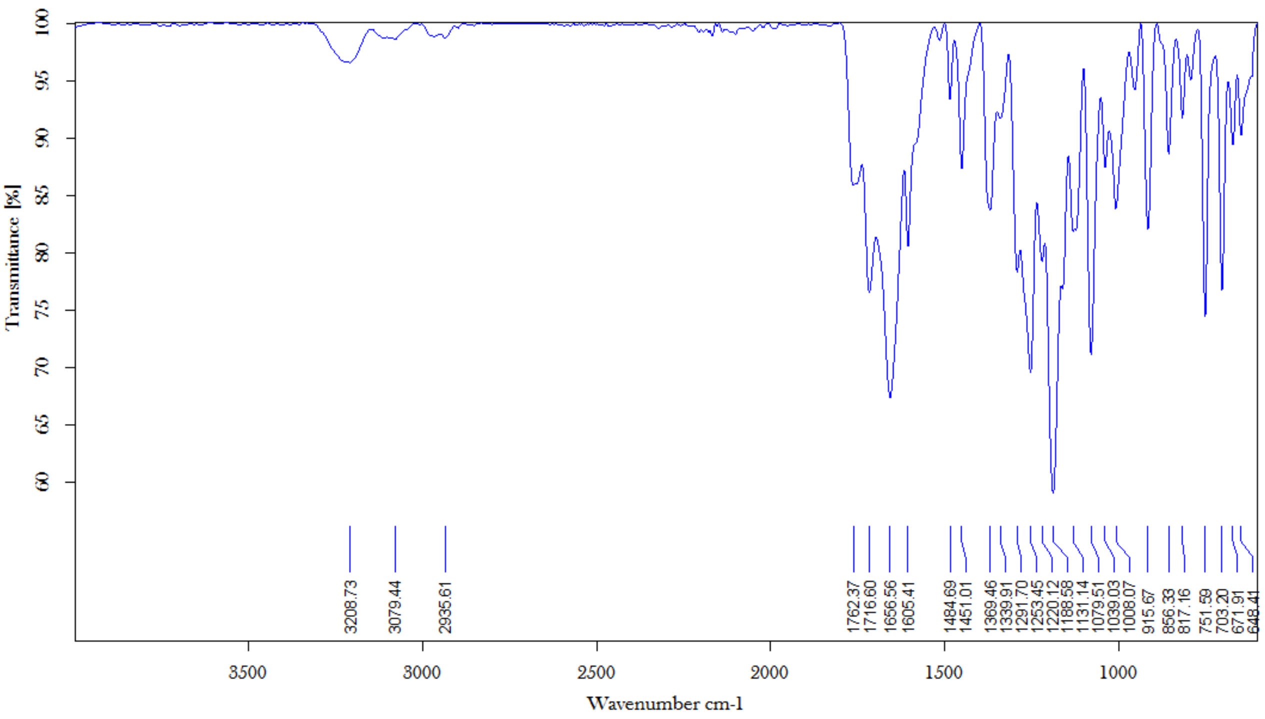

*Figure S24 IR spectrum of complex **4***

DFT optimized structures for **1a**, **1b**, **2a**, and **2b**

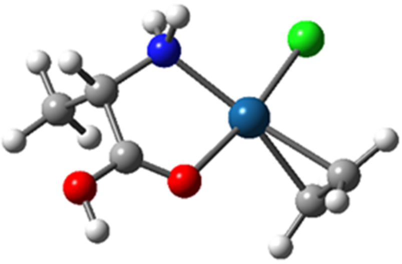

1a
0.0 (0.0)

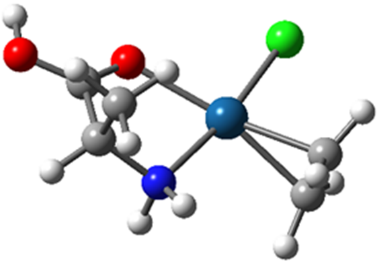

1b
15.4 (16.3)

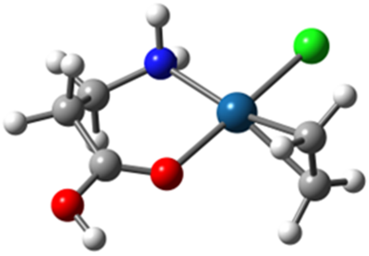

2a
0.0 (0.0)

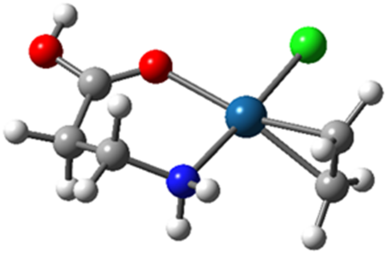

2b
19.2 (18.2)

*Figure S25 Optimized structures at the B3LYP level of isomers **1a**, **1b**, **2a** and **2b**. Relative enthalpies (free energies) at 298 K calculated at the M06-2X level are reported in kJ mol^{-1}.*

Electropherograms of **1a** - **4**

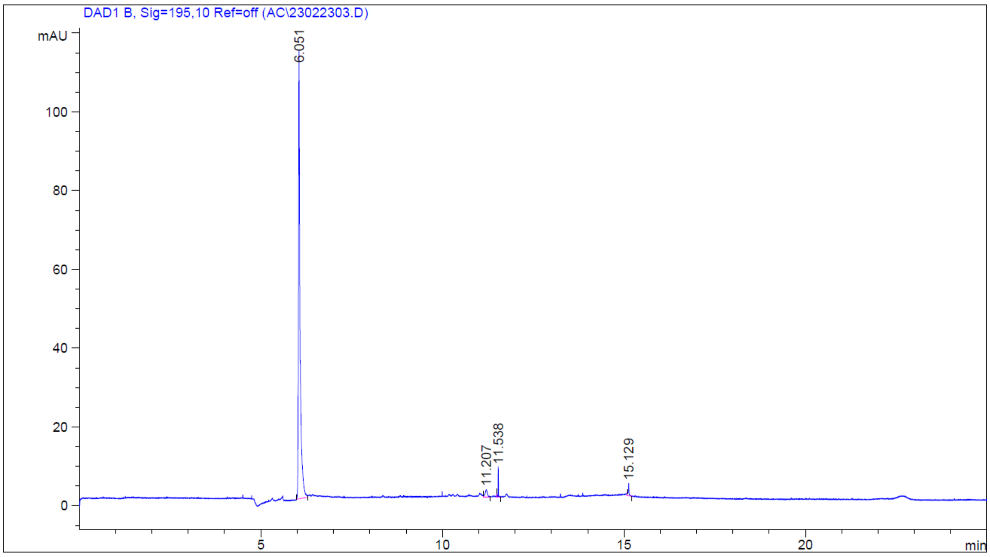

Signal 1: DAD1 B, Sig=195,10 Ref=off

Peak #	MigTime [min]	Type	Width [min]	CorrArea [mAU]	Height [mAU]	CorrArea %
1	6.051	PB	0.0462	9.61728e-1	112.39963	97.2820
2	11.207	VB	0.0703	1.20313e-2	1.73698	1.2170
3	11.538	BB	0.0180	9.12343e-3	7.34031	0.9229
4	15.129	BP	0.0302	5.71564e-3	2.98951	0.5782
Totals :				9.88599e-1	124.46643	

*Figure S26 Electropherogram of complex **1a***

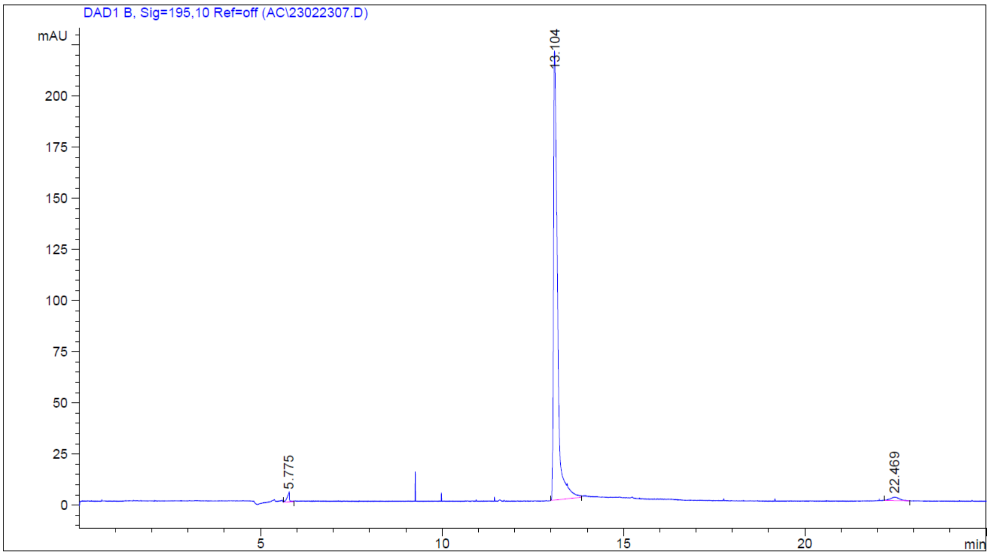

Signal 1: DAD1 B, Sig=195,10 Ref=off

Peak #	MigTime [min]	Type	Width [min]	CorrArea [mAU]	Height [mAU]	CorrArea %
1	5.775	VB	0.0704	6.63043e-2	4.43643	3.0162
2	13.104	PB	0.1148	2.11034	217.93184	95.9987
3	22.469	BB	0.2167	2.16574e-2	1.68014	0.9852
Totals :				2.19831	224.04841	

*Figure S27 Electropherogram of complex **2b***

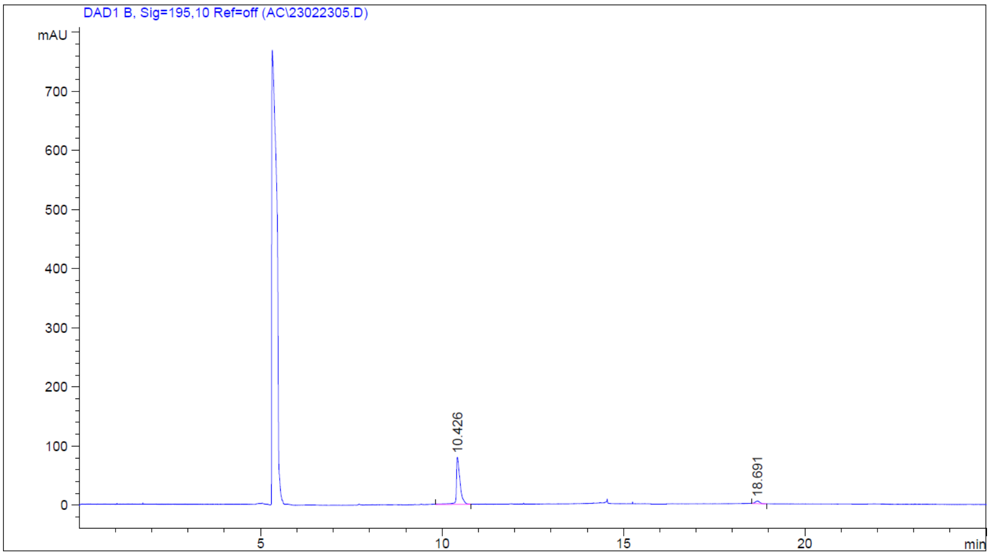

Signal 1: DAD1 B, Sig=195,10 Ref=off

Peak #	MigTime [min]	Type	Width [min]	CorrArea [mAU]	Height [mAU]	CorrArea %
1	10.426	BB	0.1073	9.05186e-1	79.31863	96.1691
2	18.691	BP	0.1329	3.60581e-2	4.58547	3.8309

Totals : 9.41244e-1 83.90410

*Figure S28 Electropherogram of complex **3**, TMG is responsible for the peak at about 5 min*

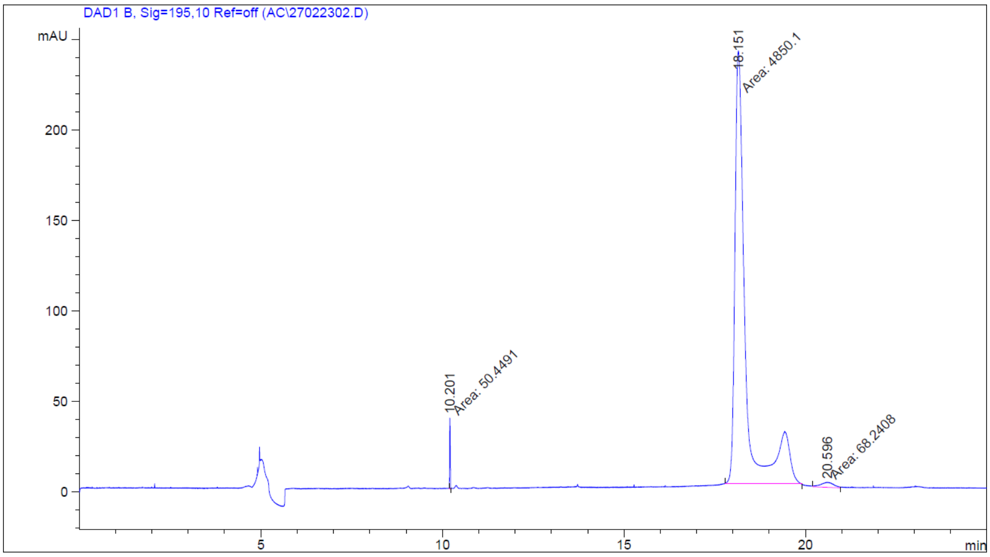

Signal 1: DAD1 B, Sig=195,10 Ref=off

Peak #	MigTime [min]	Type	Width [min]	CorrArea [mAU]	Height [mAU]	CorrArea %
1	10.201	MM	0.0215	8.24233e-2	39.15310	1.7953
2	18.151	MM	0.3378	4.45342	239.30373	97.0019
3	20.596	MM	0.4020	5.52222e-2	2.82933	1.2028

Totals : 4.59107 281.28615

*Figure S29 Electropherogram of complex **4**. The anomalous shape of the peak is probably caused by some stacking disturbance or by the non-optimal pH of the buffer. The peak at 20.60 min is due to some residual free ligand. No other impurities are detectable.*

Crystal data and structure refinements for complex **1a**

*Table S2 Crystal data and structure refinement for **1a**.*

Identification code	1a	
Empirical formula	C5 H10 Cl N O2 Pt	
Formula weight	346.68	
Temperature	173.00 K	
Wavelength	0.71073 Å	
Crystal system	Monoclinic	
Space group	$P2_1$ (no. 4)	
Unit cell dimensions	a = 9.0486(4) Å	α= 90°.
	b = 9.4748(4) Å	β= 101.6199(15)°.
	c = 10.1422(4) Å	γ = 90°.
Volume	851.71(6) $Å^3$	
Z	4	
Density (calculated)	2.704 Mg/m^3	
Absorption coefficient	16.732 mm^{-1}	
F(000)	632	
Crystal size	0.18 x 0.15 x 0.12 mm^3	
Theta range for data collection	2.050 to 27.499°.	
Index ranges	-11<=h<=11, -12<=k<=12, -13<=l<=13	
Reflections collected	20487	
Independent reflections	3898 [R(int) = 0.0677]	
Completeness to theta = 25.242°	99.8 %	
Absorption correction	Semi-empirical from equivalents	
Max. and min. transmission	0.2346 and 0.1375	
Refinement method	Full-matrix least-squares on F^2	
Data / restraints / parameters	3898 / 9 / 216	
Goodness-of-fit on F^2	1.013	
Final R indices [I>2sigma(I)]	R1 = 0.0200, wR2 = 0.0448	
R indices (all data)	R1 = 0.0210, wR2 = 0.0450	
Absolute structure parameter	-0.011(7)	
Extinction coefficient	0.0011(3)	
Largest diff. peak and hole	1.059 and -1.051 e.$Å^{-3}$	

*Table S3 Atomic coordinates (x 10^4) and equivalent isotropic displacement parameters ($Å^2$x 10^3) for complex **1a**. U(eq) is defined as one third of the trace of the orthogonalized U^{ij} tensor.*

	x	y	z	U(eq)
Pt(1)	3208(1)	7136(1)	564(1)	17(1)
Cl(1)	3688(2)	9155(2)	1795(2)	33(1)
O(1)	2618(5)	5316(5)	-457(4)	21(1)
O(2)	1102(5)	3479(5)	-611(5)	26(1)
N(1)	1474(6)	6580(6)	1490(5)	19(1)
C(1)	4341(8)	7838(10)	-986(7)	31(2)
C(2)	5400(7)	7244(10)	17(8)	30(1)
C(3)	1620(6)	4561(7)	-36(6)	20(1)
C(4)	1150(7)	5040(6)	1280(6)	21(1)
C(5)	-477(8)	4699(9)	1302(8)	37(2)
Pt(2)	2997(1)	2980(1)	5498(1)	18(1)
Cl(2)	3270(2)	872(2)	6613(2)	31(1)
O(3)	2614(5)	4849(5)	4561(4)	23(1)
O(4)	1165(5)	6747(5)	4301(4)	26(1)
N(2)	1310(7)	3660(6)	6430(6)	26(1)
C(6)	5151(8)	2747(9)	4913(9)	33(2)
C(7)	4047(8)	2149(11)	3932(7)	33(1)
C(8)	1494(7)	5584(7)	4830(6)	22(1)
C(9)	547(7)	4932(7)	5744(6)	21(1)
C(10)	111(8)	5978(8)	6733(7)	33(2)

*Table S4 Bond lengths [Å] and angles [°] for complex **1a**.*

Pt(1)-Cl(1)	2.2775(16)
Pt(1)-O(1)	2.027(4)
Pt(1)-N(1)	2.054(5)
Pt(1)-C(1)	2.148(7)
Pt(1)-C(2)	2.167(6)
O(1)-C(3)	1.290(8)
O(2)-C(3)	1.225(8)
N(1)-H(1A)	0.9100
N(1)-H(1B)	0.9100
N(1)-C(4)	1.495(8)
C(1)-H(1C)	0.95(3)
C(1)-H(1D)	0.95(3)
C(1)-C(2)	1.370(11)
C(2)-H(2A)	0.94(3)
C(2)-H(2B)	0.94(3)
C(3)-C(4)	1.548(8)
C(4)-H(4)	1.0000
C(4)-C(5)	1.512(9)
C(5)-H(5A)	0.9800
C(5)-H(5B)	0.9800
C(5)-H(5C)	0.9800
Pt(2)-Cl(2)	2.2833(16)
Pt(2)-O(3)	2.007(4)
Pt(2)-N(2)	2.055(5)
Pt(2)-C(6)	2.160(7)
Pt(2)-C(7)	2.157(7)
O(3)-C(8)	1.303(8)
O(4)-C(8)	1.235(8)
N(2)-H(2C)	0.9100
N(2)-H(2D)	0.9100
N(2)-C(9)	1.489(9)
C(6)-H(6A)	0.95(3)
C(6)-H(6B)	0.95(3)
C(6)-C(7)	1.382(12)
C(7)-H(7A)	0.95(3)
C(7)-H(7B)	0.95(3)
C(8)-C(9)	1.515(8)

C(9)-H(9)	1.0000
C(9)-C(10)	1.518(9)
C(10)-H(10A)	0.9800
C(10)-H(10B)	0.9800
C(10)-H(10C)	0.9800
O(1)-Pt(1)-Cl(1)	174.91(13)
O(1)-Pt(1)-N(1)	82.44(19)
O(1)-Pt(1)-C(1)	90.3(3)
O(1)-Pt(1)-C(2)	94.0(3)
N(1)-Pt(1)-Cl(1)	92.50(16)
N(1)-Pt(1)-C(1)	159.3(3)
N(1)-Pt(1)-C(2)	162.3(3)
C(1)-Pt(1)-Cl(1)	94.3(2)
C(1)-Pt(1)-C(2)	37.0(3)
C(2)-Pt(1)-Cl(1)	91.0(2)
C(3)-O(1)-Pt(1)	115.6(4)
Pt(1)-N(1)-H(1A)	109.8
Pt(1)-N(1)-H(1B)	109.8
H(1A)-N(1)-H(1B)	108.3
C(4)-N(1)-Pt(1)	109.3(4)
C(4)-N(1)-H(1A)	109.8
C(4)-N(1)-H(1B)	109.8
Pt(1)-C(1)-H(1C)	104(6)
Pt(1)-C(1)-H(1D)	106(6)
H(1C)-C(1)-H(1D)	125(8)
C(2)-C(1)-Pt(1)	72.2(4)
C(2)-C(1)-H(1C)	117(6)
C(2)-C(1)-H(1D)	115(5)
Pt(1)-C(2)-H(2A)	108(6)
Pt(1)-C(2)-H(2B)	114(5)
C(1)-C(2)-Pt(1)	70.8(4)
C(1)-C(2)-H(2A)	136(6)
C(1)-C(2)-H(2B)	125(5)
H(2A)-C(2)-H(2B)	97(8)
O(1)-C(3)-C(4)	117.1(5)
O(2)-C(3)-O(1)	122.2(6)
O(2)-C(3)-C(4)	120.7(6)
N(1)-C(4)-C(3)	109.2(5)

N(1)-C(4)-H(4)	107.7
N(1)-C(4)-C(5)	111.7(5)
C(3)-C(4)-H(4)	107.7
C(5)-C(4)-C(3)	112.8(5)
C(5)-C(4)-H(4)	107.7
C(4)-C(5)-H(5A)	109.5
C(4)-C(5)-H(5B)	109.5
C(4)-C(5)-H(5C)	109.5
H(5A)-C(5)-H(5B)	109.5
H(5A)-C(5)-H(5C)	109.5
H(5B)-C(5)-H(5C)	109.5
O(3)-Pt(2)-Cl(2)	175.84(13)
O(3)-Pt(2)-N(2)	82.4(2)
O(3)-Pt(2)-C(6)	92.2(3)
O(3)-Pt(2)-C(7)	91.9(3)
N(2)-Pt(2)-Cl(2)	93.60(16)
N(2)-Pt(2)-C(6)	162.5(3)
N(2)-Pt(2)-C(7)	158.7(3)
C(6)-Pt(2)-Cl(2)	91.9(2)
C(7)-Pt(2)-Cl(2)	91.5(3)
C(7)-Pt(2)-C(6)	37.3(3)
C(8)-O(3)-Pt(2)	116.1(4)
Pt(2)-N(2)-H(2C)	109.5
Pt(2)-N(2)-H(2D)	109.5
H(2C)-N(2)-H(2D)	108.1
C(9)-N(2)-Pt(2)	110.9(4)
C(9)-N(2)-H(2C)	109.5
C(9)-N(2)-H(2D)	109.5
Pt(2)-C(6)-H(6A)	109(5)
Pt(2)-C(6)-H(6B)	109(5)
H(6A)-C(6)-H(6B)	112(6)
C(7)-C(6)-Pt(2)	71.2(4)
C(7)-C(6)-H(6A)	130(5)
C(7)-C(6)-H(6B)	114(5)
Pt(2)-C(7)-H(7A)	108(4)
Pt(2)-C(7)-H(7B)	105(5)
C(6)-C(7)-Pt(2)	71.5(4)
C(6)-C(7)-H(7A)	123(4)
C(6)-C(7)-H(7B)	118(5)

H(7A)-C(7)-H(7B)	117(7)
O(3)-C(8)-C(9)	118.0(6)
O(4)-C(8)-O(3)	121.0(6)
O(4)-C(8)-C(9)	120.9(6)
N(2)-C(9)-C(8)	110.5(5)
N(2)-C(9)-H(9)	106.9
N(2)-C(9)-C(10)	112.2(5)
C(8)-C(9)-H(9)	106.9
C(8)-C(9)-C(10)	113.1(6)
C(10)-C(9)-H(9)	106.9
C(9)-C(10)-H(10A)	109.5
C(9)-C(10)-H(10B)	109.5
C(9)-C(10)-H(10C)	109.5
H(10A)-C(10)-H(10B)	109.5
H(10A)-C(10)-H(10C)	109.5
H(10B)-C(10)-H(10C)	109.5

Symmetry transformations used to generate equivalent atoms:

*Table S5 Anisotropic displacement parameters ($Å^2 x 10^3$) for complex **1a**. The anisotropic displacement factor exponent takes the form: $-2\pi^2[h^2 a^{*2}U^{11} + ... + 2 h k a^* b^* U^{12}]$*

	U^{11}	U^{22}	U^{33}	U^{23}	U^{13}	U^{12}
Pt(1)	21(1)	15(1)	17(1)	0(1)	6(1)	-1(1)
Cl(1)	52(1)	19(1)	30(1)	-8(1)	13(1)	-9(1)
O(1)	24(2)	19(2)	21(2)	-2(2)	9(2)	-1(2)
O(2)	25(2)	23(2)	30(2)	-10(2)	6(2)	-5(2)
N(1)	23(2)	15(2)	20(2)	-1(2)	5(2)	-1(2)
C(1)	39(4)	34(4)	26(3)	2(3)	19(3)	-8(3)
C(2)	25(3)	28(4)	41(4)	-5(4)	17(3)	-1(3)
C(3)	19(3)	19(3)	21(3)	-1(2)	2(2)	4(2)
C(4)	29(3)	18(3)	16(3)	-1(2)	7(2)	1(2)
C(5)	34(4)	44(4)	40(4)	-16(3)	24(3)	-16(3)
Pt(2)	21(1)	16(1)	17(1)	-1(1)	6(1)	-1(1)
Cl(2)	43(1)	20(1)	34(1)	7(1)	16(1)	5(1)
O(3)	26(2)	19(2)	29(2)	4(2)	14(2)	4(2)
O(4)	33(2)	21(3)	26(2)	4(2)	8(2)	4(2)
N(2)	35(3)	19(3)	25(3)	2(2)	14(2)	0(2)
C(6)	29(3)	29(4)	47(4)	0(3)	19(3)	6(3)
C(7)	42(4)	32(4)	29(3)	-2(4)	19(3)	5(4)
C(8)	25(3)	23(3)	18(3)	-1(3)	7(2)	-4(2)
C(9)	23(3)	26(3)	16(3)	1(2)	7(2)	2(2)
C(10)	40(4)	32(4)	30(3)	6(3)	13(3)	22(3)

*Table S6 Hydrogen coordinates (x 10^4) and isotropic displacement parameters (Å^{2}x 10^3) for complex **1a**.*

	x	y	z	U(eq)
H(1A)	1734	6770	2387	23
H(1B)	635	7090	1139	23
H(1C)	3940(100)	7260(80)	-1740(60)	50(20)
H(1D)	4310(100)	8840(30)	-980(90)	40(20)
H(2A)	6100(80)	7580(100)	760(60)	50(30)
H(2B)	5850(90)	6360(50)	-40(80)	40(20)
H(4)	1796	4520	2043	25
H(5A)	-698	4970	2174	55
H(5B)	-648	3684	1162	55
H(5C)	-1140	5222	583	55
H(2C)	621	2957	6416	31
H(2D)	1706	3870	7305	31
H(6A)	5630(80)	3640(50)	4930(80)	25(19)
H(6B)	5740(80)	2090(70)	5500(60)	26(19)
H(7A)	3870(80)	1160(30)	3870(70)	22(18)
H(7B)	3640(80)	2700(80)	3160(50)	30(20)
H(9)	-412	4601	5154	26
H(10A)	-568	5520	7243	50
H(10B)	-402	6787	6240	50
H(10C)	1021	6302	7355	50

*Table S7 Torsion angles [°] for complex **1a**.*

Pt(1)-O(1)-C(3)-O(2)	175.7(4)
Pt(1)-O(1)-C(3)-C(4)	-7.2(6)
Pt(1)-N(1)-C(4)-C(3)	-26.7(5)
Pt(1)-N(1)-C(4)-C(5)	-152.1(4)
O(1)-C(3)-C(4)-N(1)	23.1(7)
O(1)-C(3)-C(4)-C(5)	147.9(6)
O(2)-C(3)-C(4)-N(1)	-159.8(5)
O(2)-C(3)-C(4)-C(5)	-35.0(8)
Pt(2)-O(3)-C(8)-O(4)	-179.8(4)
Pt(2)-O(3)-C(8)-C(9)	-3.5(7)
Pt(2)-N(2)-C(9)-C(8)	-15.7(6)
Pt(2)-N(2)-C(9)-C(10)	-142.9(5)
O(3)-C(8)-C(9)-N(2)	13.1(8)
O(3)-C(8)-C(9)-C(10)	139.8(6)
O(4)-C(8)-C(9)-N(2)	-170.6(5)
O(4)-C(8)-C(9)-C(10)	-43.9(8)

Symmetry transformations used to generate equivalent atoms:

*Table S8 Hydrogen bonds for complex **1a** [Å and °].*

D-H...A	d(D-H)	d(H...A)	d(D...A)	<(DHA)
N(1)-H(1A)...O(4)	0.91	2.11	2.924(7)	149.0
N(1)-H(1B)...O(2)#1	0.91	2.04	2.937(7)	170.0

Symmetry transformations used to generate equivalent atoms:

#1 -x,y+1/2,-z

Crystal data and structure refinements for complex **1b**

*Table S9 Crystal data and structure refinement for **1b**.*

Identification code	1b	
Empirical formula	C5 H10 Cl N O2 Pt	
Formula weight	346.68	
Temperature	173.00 K	
Wavelength	0.71073 Å	
Crystal system	Orthorhombic	
Space group	$P2_12_12_1$ (no. 19)	
Unit cell dimensions	a = 7.3886(2) Å	α= 90°.
	b = 9.2775(3) Å	β= 90°.
	c = 11.7149(3) Å	γ = 90°.
Volume	803.03(4) $Å^3$	
Z	4	
Density (calculated)	2.868 Mg/m^3	
Absorption coefficient	17.746 mm^{-1}	
F(000)	632	
Crystal size	0.22 x 0.17 x 0.05 mm^3	
Theta range for data collection	2.801 to 28.697°.	
Index ranges	-9<=h<=9, -12<=k<=12, -15<=l<=15	
Reflections collected	18312	
Independent reflections	2070 [R(int) = 0.0458]	
Completeness to theta = 25.242°	99.4 %	
Absorption correction	Semi-empirical from equivalents	
Max. and min. transmission	0.4044 and 0.2318	
Refinement method	Full-matrix least-squares on F^2	
Data / restraints / parameters	2070 / 4 / 109	
Goodness-of-fit on F^2	1.058	
Final R indices [I>2sigma(I)]	R1 = 0.0177, wR2 = 0.0402	
R indices (all data)	R1 = 0.0194, wR2 = 0.0413	
Absolute structure parameter	0.023(11)	
Extinction coefficient	0.0027(3)	
Largest diff. peak and hole	1.239 and -0.711 e.$Å^{-3}$	

*Table S10 Atomic coordinates (x 10^4) and equivalent isotropic displacement parameters ($Å^2$x 10^3) for complex **1b**. U(eq) is defined as one third of the trace of the orthogonalized U^{ij} tensor.*

	x	y	z	U(eq)
Pt(1)	7487(1)	2545(1)	5396(1)	18(1)
Cl(1)	7231(2)	614(1)	4174(1)	28(1)
O(1)	7492(7)	3977(3)	4080(2)	23(1)
O(2)	7502(8)	6301(3)	3709(3)	37(1)
N(1)	7852(6)	4387(4)	6315(3)	20(1)
C(1)	8385(8)	1186(9)	6742(6)	26(1)
C(2)	6524(8)	1301(9)	6805(6)	26(2)
C(3)	7398(9)	5320(5)	4392(3)	23(1)
C(4)	7094(6)	5622(5)	5662(4)	21(1)
C(5)	7844(8)	7049(6)	6048(4)	29(1)

Table S11 Bond lengths [Å] and angles [°] for complex **1b**.

Pt(1)-Cl(1)	2.3018(12)
Pt(1)-O(1)	2.035(3)
Pt(1)-N(1)	2.037(4)
Pt(1)-C(1)	2.125(7)
Pt(1)-C(2)	2.136(7)
O(1)-C(3)	1.301(5)
O(2)-C(3)	1.215(5)
N(1)-H(1A)	0.9100
N(1)-H(1B)	0.9100
N(1)-C(4)	1.488(6)
C(1)-H(1C)	0.94(3)
C(1)-H(1D)	0.95(3)
C(1)-C(2)	1.382(7)
C(2)-H(2A)	0.94(3)
C(2)-H(2B)	0.94(2)
C(3)-C(4)	1.530(6)
C(4)-H(4)	1.0000
C(4)-C(5)	1.505(7)
C(5)-H(5A)	0.9800
C(5)-H(5B)	0.9800
C(5)-H(5C)	0.9800
O(1)-Pt(1)-Cl(1)	92.10(9)
O(1)-Pt(1)-N(1)	81.54(13)
O(1)-Pt(1)-C(1)	161.6(2)
O(1)-Pt(1)-C(2)	159.8(2)
N(1)-Pt(1)-Cl(1)	172.99(11)
N(1)-Pt(1)-C(1)	93.7(2)
N(1)-Pt(1)-C(2)	95.1(2)
C(1)-Pt(1)-Cl(1)	91.4(2)
C(1)-Pt(1)-C(2)	37.8(2)
C(2)-Pt(1)-Cl(1)	91.9(2)
C(3)-O(1)-Pt(1)	114.4(3)
Pt(1)-N(1)-H(1A)	109.9
Pt(1)-N(1)-H(1B)	109.9
H(1A)-N(1)-H(1B)	108.3
C(4)-N(1)-Pt(1)	108.9(3)

C(4)-N(1)-H(1A)	109.9
C(4)-N(1)-H(1B)	109.9
Pt(1)-C(1)-H(1C)	105(5)
Pt(1)-C(1)-H(1D)	111(4)
H(1C)-C(1)-H(1D)	118(5)
C(2)-C(1)-Pt(1)	71.5(5)
C(2)-C(1)-H(1C)	120(4)
C(2)-C(1)-H(1D)	119(4)
Pt(1)-C(2)-H(2A)	111(4)
Pt(1)-C(2)-H(2B)	112(3)
C(1)-C(2)-Pt(1)	70.7(5)
C(1)-C(2)-H(2A)	122(4)
C(1)-C(2)-H(2B)	119(3)
H(2A)-C(2)-H(2B)	113(5)
O(1)-C(3)-C(4)	117.1(4)
O(2)-C(3)-O(1)	122.0(4)
O(2)-C(3)-C(4)	120.9(4)
N(1)-C(4)-C(3)	107.7(4)
N(1)-C(4)-H(4)	107.6
N(1)-C(4)-C(5)	112.6(4)
C(3)-C(4)-H(4)	107.6
C(5)-C(4)-C(3)	113.5(4)
C(5)-C(4)-H(4)	107.6
C(4)-C(5)-H(5A)	109.5
C(4)-C(5)-H(5B)	109.5
C(4)-C(5)-H(5C)	109.5
H(5A)-C(5)-H(5B)	109.5
H(5A)-C(5)-H(5C)	109.5
H(5B)-C(5)-H(5C)	109.5

Symmetry transformations used to generate equivalent atoms:

*Table S12 Anisotropic displacement parameters ($Å^2$x 10^3) for complex **1b**. The anisotropic displacement factor exponent takes the form: $-2\pi^2[h^2 a^{*2}U^{11} + ... + 2 h k a^* b^* U^{12}]$*

	U^{11}	U^{22}	U^{33}	U^{23}	U^{13}	U^{12}
Pt(1)	22(1)	20(1)	13(1)	-1(1)	-1(1)	0(1)
Cl(1)	31(1)	26(1)	26(1)	-10(1)	-5(1)	4(1)
O(1)	32(2)	25(2)	12(1)	-1(1)	0(2)	-1(2)
O(2)	65(3)	29(2)	16(1)	5(1)	0(3)	-1(3)
N(1)	27(2)	22(2)	10(2)	4(1)	-2(2)	-1(2)
C(1)	33(3)	25(4)	21(3)	5(3)	-7(3)	0(3)
C(2)	31(3)	29(4)	18(3)	6(3)	2(2)	-3(3)
C(3)	25(2)	32(2)	11(2)	0(2)	-4(2)	0(3)
C(4)	24(2)	21(2)	18(2)	4(2)	-2(2)	0(2)
C(5)	43(3)	26(2)	17(2)	2(2)	-6(2)	-1(2)

*Table S13 Hydrogen coordinates (x 10^4) and isotropic displacement parameters ($Å^2$x 10^3) for complex **1b**.*

	x	y	z	U(eq)
H(1A)	7283	4310	7002	24
H(1B)	9053	4530	6445	24
H(1C)	9120(70)	1780(70)	7200(50)	35(19)
H(1D)	8910(80)	320(50)	6440(50)	32(19)
H(2A)	5940(70)	1890(60)	7350(40)	24(16)
H(2B)	5800(50)	520(40)	6570(40)	3(12)
H(4)	5758	5639	5798	25
H(5A)	9154	7069	5916	43
H(5B)	7599	7180	6863	43
H(5C)	7269	7828	5615	43

*Table S14 Torsion angles [°] for complex **1b**.*

Pt(1)-O(1)-C(3)-O(2)	175.3(6)
Pt(1)-O(1)-C(3)-C(4)	-6.4(7)
Pt(1)-N(1)-C(4)-C(3)	-34.1(5)
Pt(1)-N(1)-C(4)-C(5)	-160.0(3)
O(1)-C(3)-C(4)-N(1)	27.4(7)
O(1)-C(3)-C(4)-C(5)	152.8(6)
O(2)-C(3)-C(4)-N(1)	-154.3(6)
O(2)-C(3)-C(4)-C(5)	-28.9(8)

Symmetry transformations used to generate equivalent atoms:

*Table S15 Hydrogen bonds for complex **1b** [Å and °].*

D-H...A	d(D-H)	d(H...A)	d(D...A)	<(DHA)
N(1)-H(1A)...O(2)#1	0.91	2.08	2.887(5)	146.5
N(1)-H(1B)...Cl(1)#2	0.91	2.46	3.286(4)	150.8

Symmetry transformations used to generate equivalent atoms:

#1 -x+3/2,-y+1,z+1/2   #2 x+1/2,-y+1/2,-z+1

NMR study for the synthesis of complex **1**

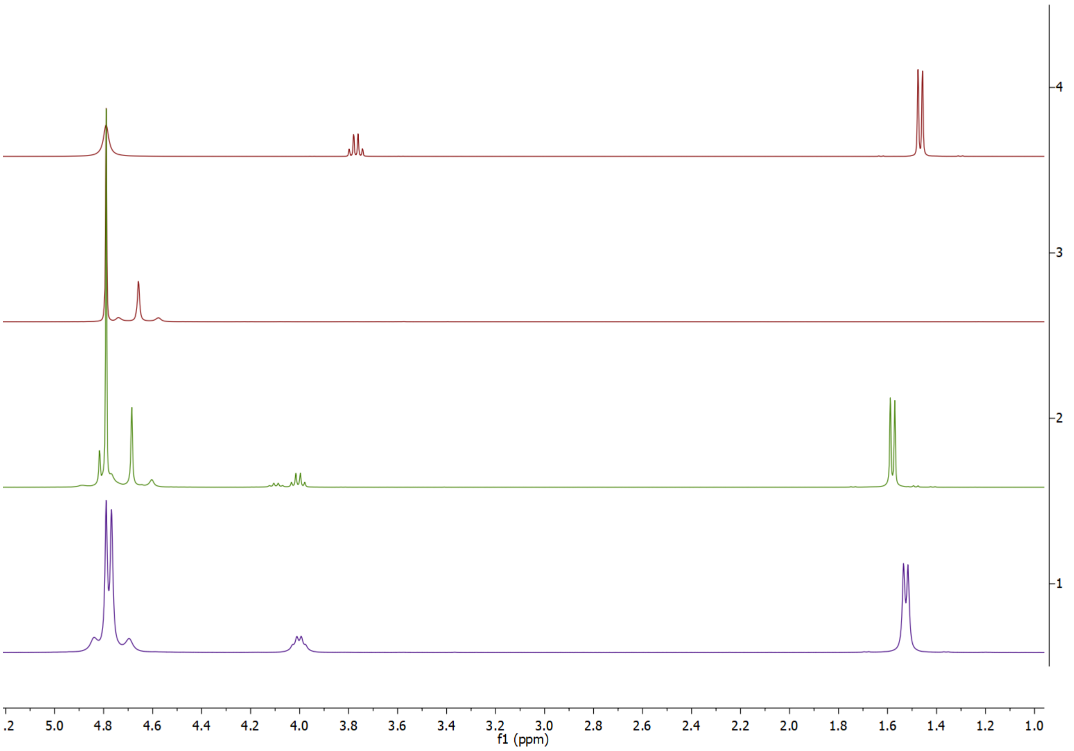

*Figure S30 from above: 4) ^{1}H-NMR spectrum of L-alanine in D_2O; 3) ^{1}H-NMR spectrum of ZS in D_2O; 2) ^{1}H-NMR of the synthesis of complex **1** without base; 1) ^{1}H-NMR of the precipitate from the synthesis of complex **1** with base in D_2O.*

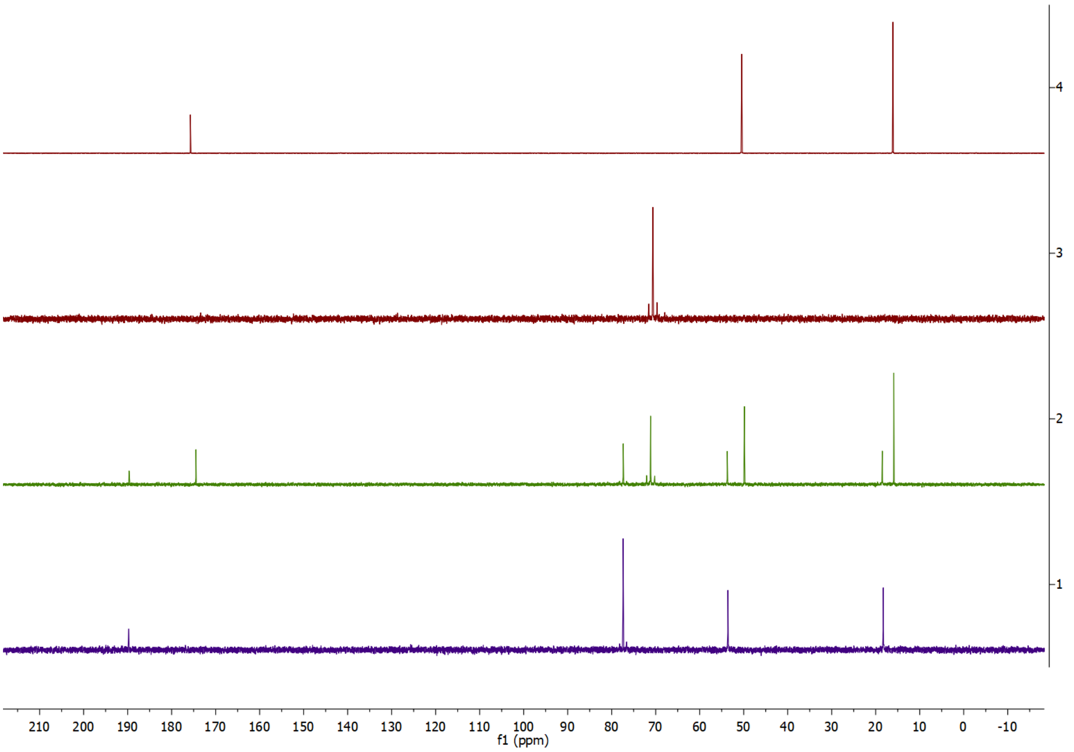

*Figure S31 from above: 4) ^{13}C-NMR spectrum of L-alanine in D_2O; 3) ^{13}C-NMR spectrum of ZS in D_2O; 2) ^{13}C-NMR of the synthesis of complex **1** without base; 1) ^{13}C-NMR of the precipitate from the synthesis of complex **1** with base in D_2O.*

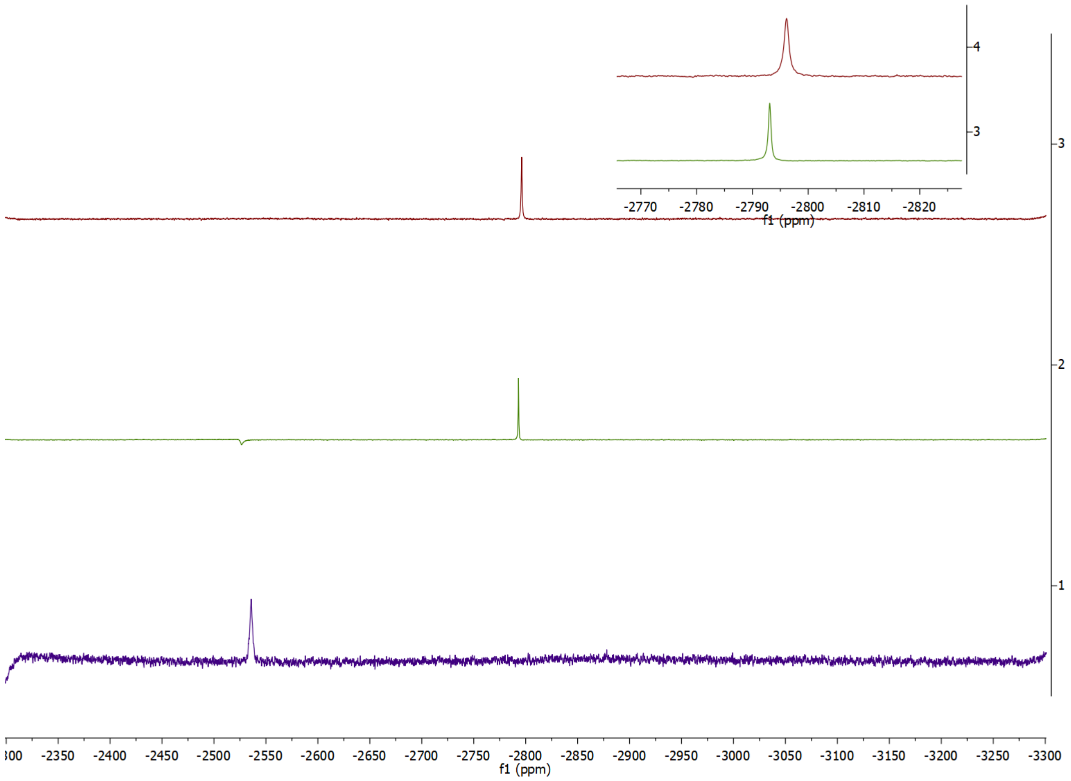

*Figure S32 from above: 3) ^{195}Pt-NMR spectrum of ZS in D_2O; 2) ^{195}Pt-NMR of the synthesis of complex **1** without base; 1) ^{195}Pt-NMR of the precipitate from the synthesis of complex **1** with base in D_2O.*

NMR investigation of the stability of complex **2** in an aqueous solution

A sample was prepared by dissolving 15 mg of complex **2** in 500 µL of deuterated methanol (CD_3OD). ^{1}H- and ^{195}Pt-NMR spectra were recorded as references. The same sample was divided in 2 portions of 250 µL each. The first portion (A) was diluted with 250 µL of CD_3OD. The second (B) was diluted with 250 µL of deuterated water (D_2O). ^{1}H- and ^{195}Pt-NMR spectra were recorded at different time points. The NMR tubes were stored at room temperature (rt) with the solution protected from light between one experiment and the other.

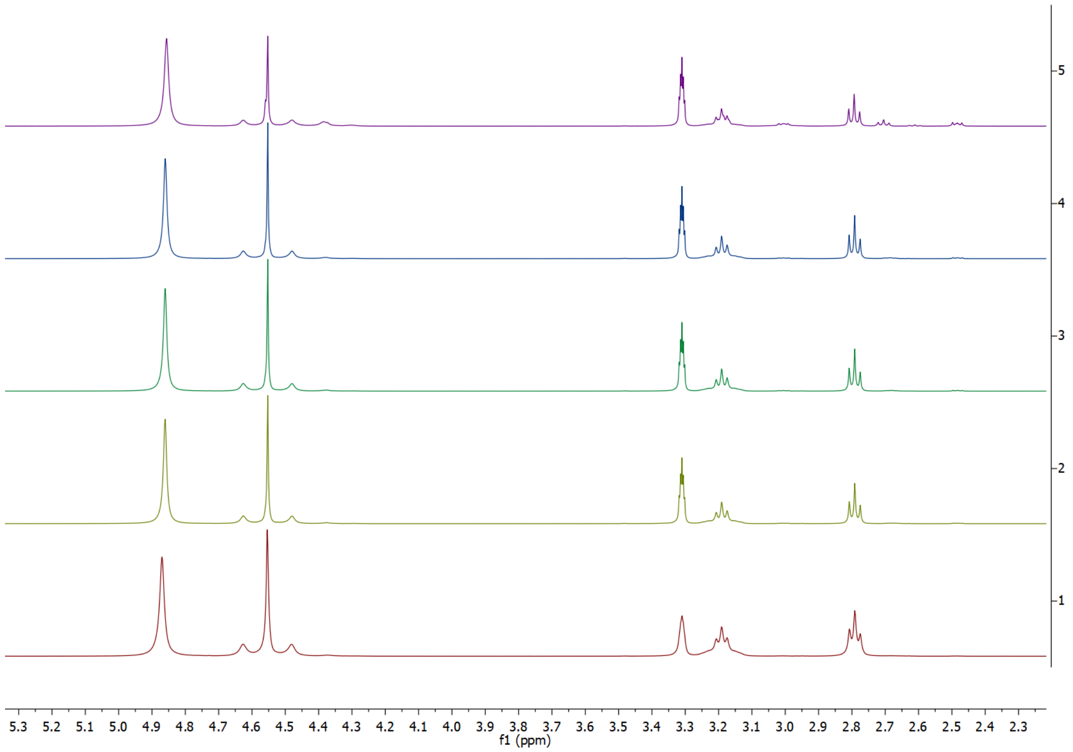

*Figure S33 Stacked ^{1}H-NMR spectra of complex **2** in CD_3OD at different time points: 1) reference 2) t = 0 h; 3) t = 1 h; 4) t = 2 h; 5) t = 72 h*

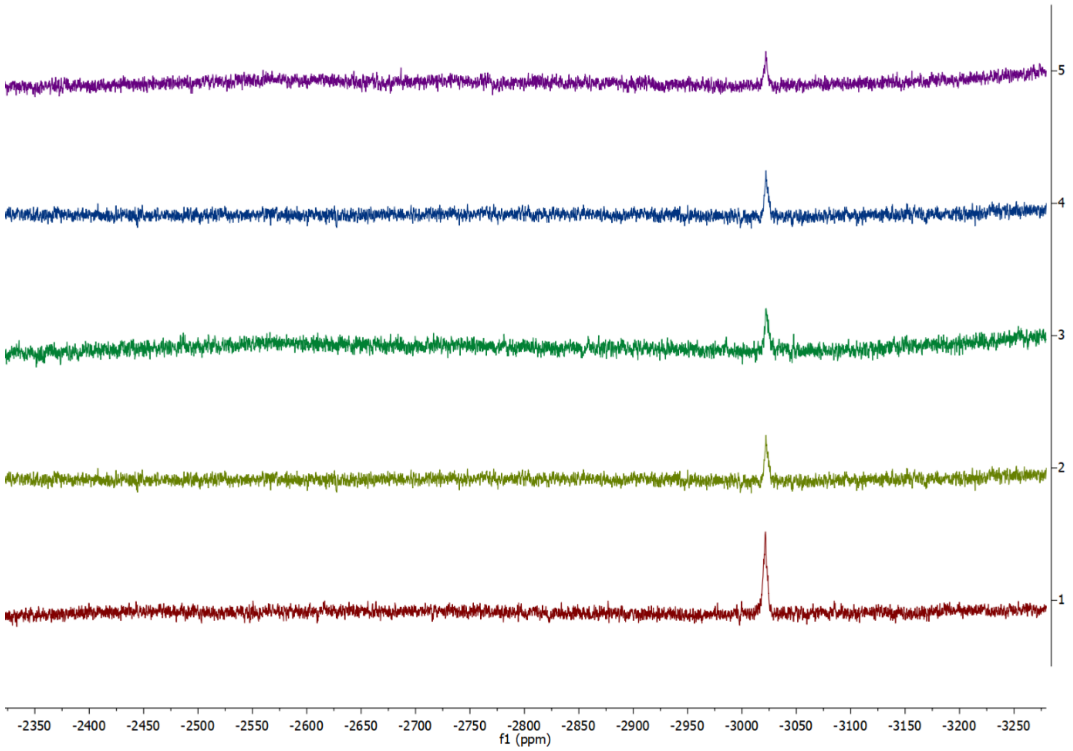

*Figure S 34 Stacked ^{195}Pt-NMR spectra of complex **2** in CD_3OD at different time points: 1) reference 2) t = 0 h; 3) t = 1 h; 4) t = 2 h; 5) t = 72 h*

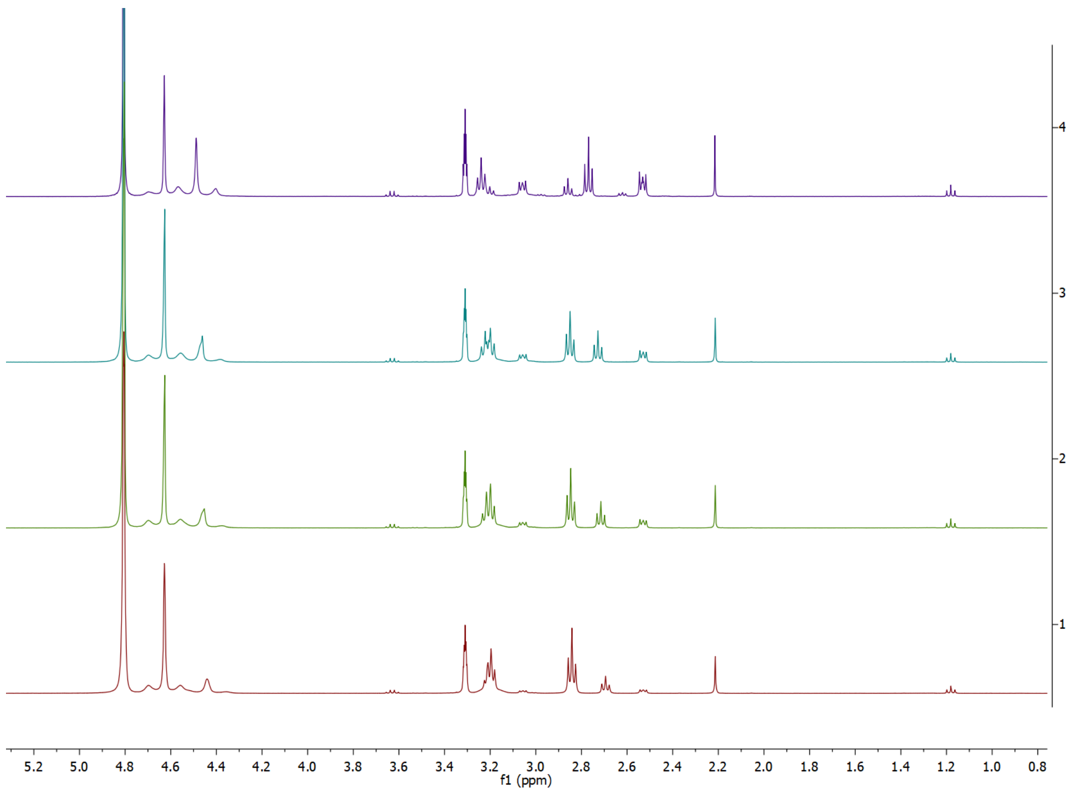

*Figure S35 Stacked ^{1}H-NMR spectra of complex **2** in D_2O\\CD_3OD 1:1 at different time points: 1) t = 0 h; 2) t = 1 h; 3) t = 2 h; 4) t = 72 h*

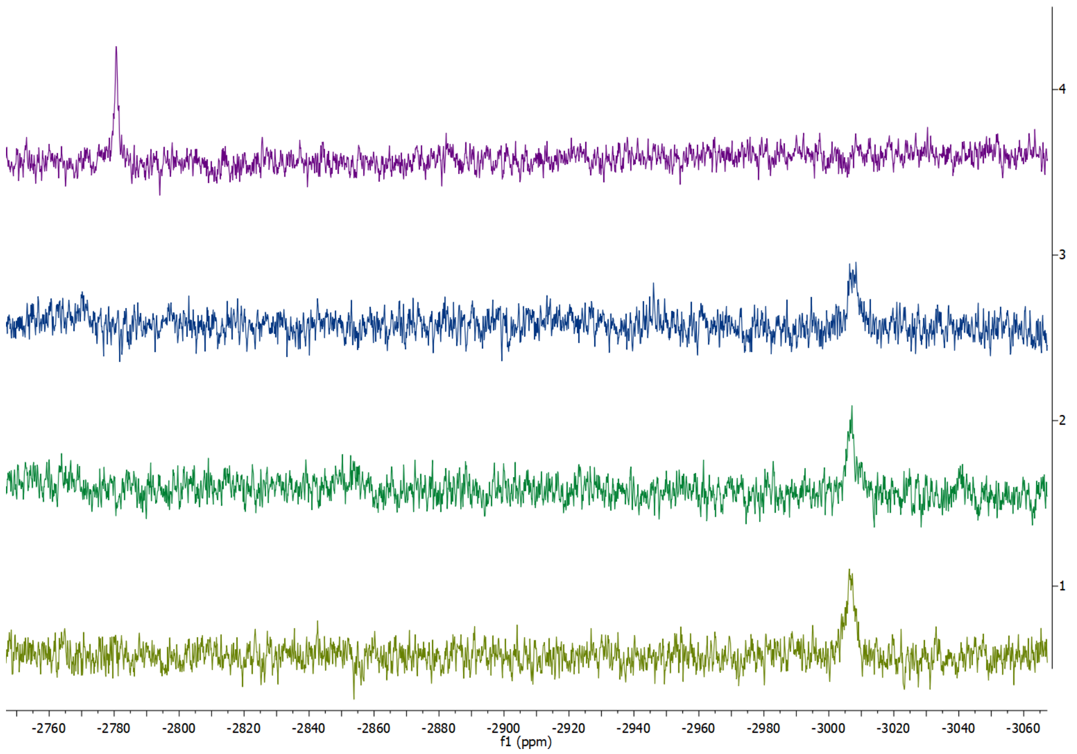

Figure S36 Stacked ^{195}Pt-NMR spectra of complex 2 in D2O\CD3OD 1:1 at different time points: 1) t = 0 h; 2) t = 1 h; 3) t = 2 h; 4) t = 72 h

NMR study for the dissolution of ZS in a highly concentrated TMG solution

To evaluate if the coordination of TMG to platinum is responsible for the diminished stability of the compounds tested, we incubated ZS with a highly concentrated betaine solution and followed the reaction via NMR spectroscopy. As first attempt, we tried to stick to the experimental conditions employed by Kadokawa et al.[6], dissolving 60 µmol of ZS in 1 mL of 50 % w/v TMG solution, prepared using deuterium oxide as solvent, and recording ^{195}Pt-NMR spectra at different time-points. Unfortunately, the high concentration of TMG completely suppressed the signal in the platinum spectrum. For this reason, we decided to decrease the amount of TMG (200 mM), while keeping it in a large excess compared to ZS (60 µmol).

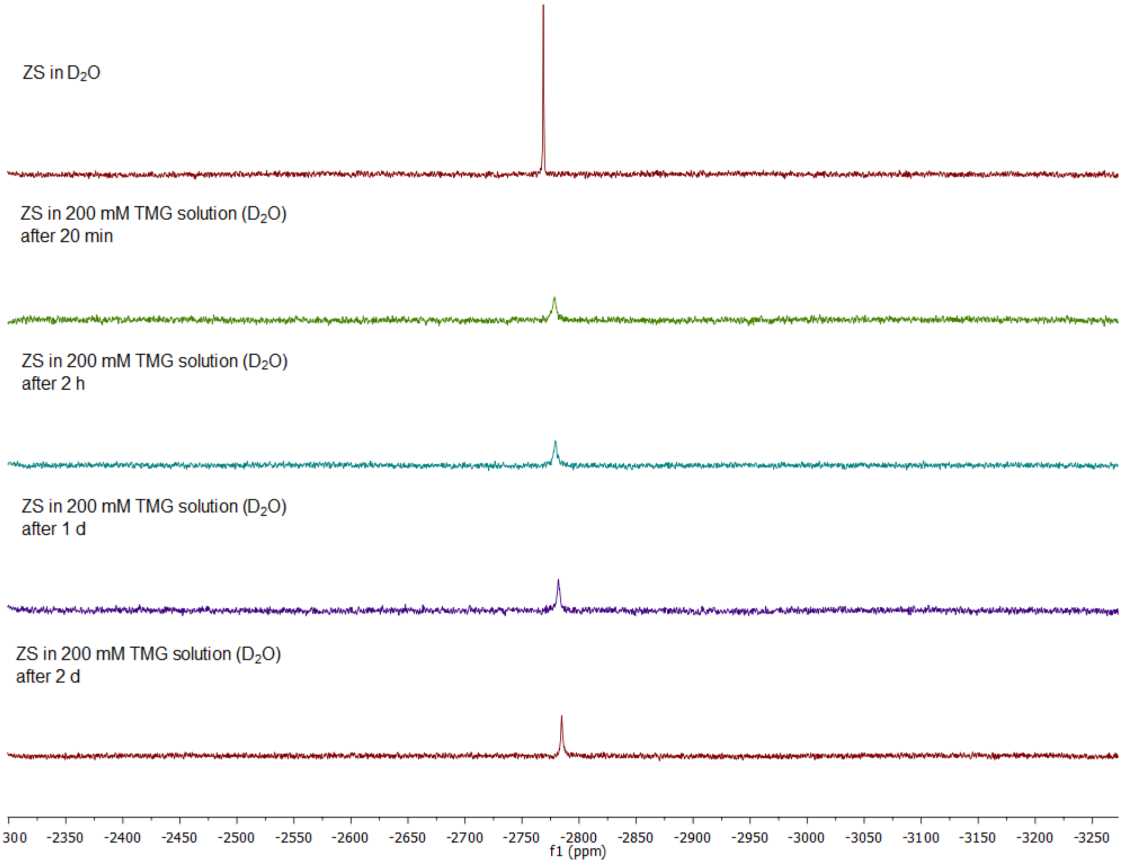

Figure S37 Comparison of the ^{195}Pt-NMR spectra of ZS in deuterium oxide and of ZS in a concentrated solution (200 nM) of TMG in deuterium oxide. The latter solution was monitored over-time up to 2 days after dissolution of ZS.

Biological data

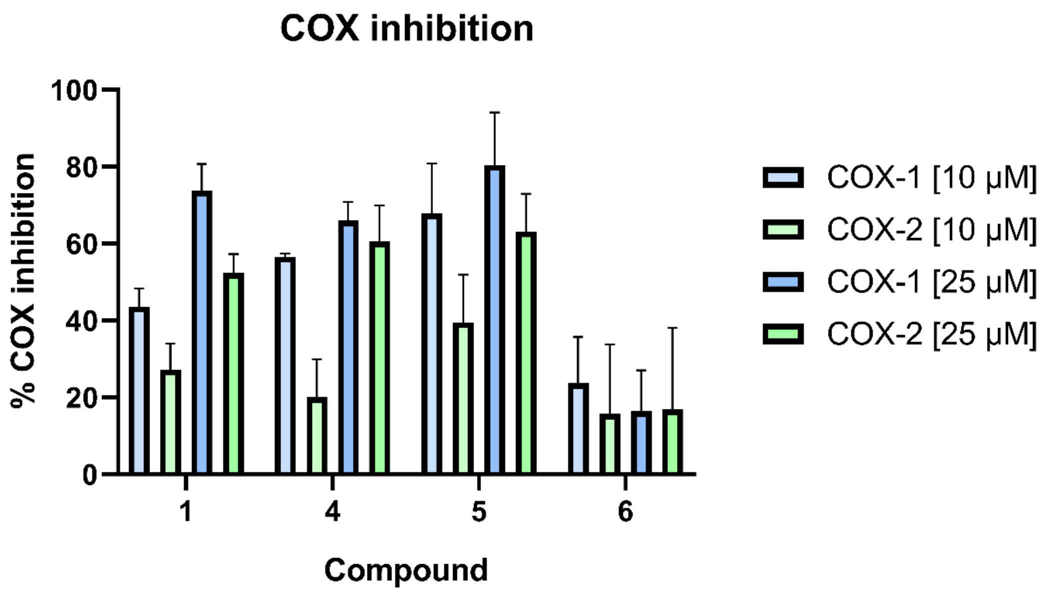

*Figure S38 Inhibition of COX-1/-2 isoenzymes by **1**, **4**, **5** and **6** at 10 µM and 25 µM; mean of 3 independent experiments ± SEM*

Statistical analysis

Identification of outliers:

Groups investigated: single values of residual metabolic activity (%) of MDA-MB-231 and A2780cis after treatment with 25 µM of compounds **1**, **4**, **5**, **6**, and cisplatin.

Method employed: Grubbs (Alpha = 0.05)

Results: in case of A2780cis cell line, one outlier (from a pool of five values) was found for compound **5** and therefore excluded from the calculations.

Multiple comparison:

Groups investigated: The means calculated from the refined values of residual metabolic activity (%) of MDA-MB-231 and A2780cis after treatment with 25 µM of compounds **1**, **4**, **5**, **6**, and cisplatin.

Method employed: An ordinary one-way ANOVA test was employed using the Sidak statistical hypothesis testing and a confidence interval of 95 %.

Results: The results are depicted in Table S17:

*Table S17 Significance of values determined for MDA-MB-231 and A2780cis incubated with compounds **1**, **4**, **5**, and **6** at 25 µM concentration. The significance is calculated in relation to the reference (cisplatin). Diff stays for difference; CI stays for Confidence Interval.*

Sidak's multiple comparisons test	Mean Diff	95,00% CI of diff	Significance	Summary	Adjusted P Value
MDA-MB-231					
Cisplatin vs. **1**	-100,5	-125,2 to -75,78	Yes	****	<0,0001
Cisplatin vs. **4**	0,996	-23,71 to 25,71	No	ns	>0,9999
Cisplatin vs. **5**	-90,69	-115,4 to -65,98	Yes	****	<0,0001
Cisplatin vs. **6**	-91,45	-116,2 to -66,74	Yes	****	<0,0001
A2780cis					
Cisplatin vs. **1**	-102,1	-138,4 to -65,75	Yes	****	<0,0001
Cisplatin vs. **4**	-28,41	-64,74 to 7,907	No	ns	0,1641
Cisplatin vs. **5**	-107,5	-145,5 to -69,48	Yes	****	<0,0001
Cisplatin vs. **6**	-103,5	-139,8 to -67,15	Yes	****	<0,0001

Parameters correlation:

Groups investigated: 3 groups of data were considered: a) the COX-1 inhibitory effect, b) the COX-2 inhibitory effect, c) the residual metabolic activity of MDA-MB-231 cell lines. For each group the mean values were considered for compounds **1**, **4**, and **5**. Only the values obtained from the incubation of cells or enzymes with the above-mentioned compounds at a concentration of 25 µM were considered. Since only MDA-MB-231 expresses both COX-1 and COX-2 isoenzymes, data obtained employing the A2780cis cell lines were excluded.

Method employed: Pearson correlation coefficient was employed using two-tailed P-value settings and a confidence interval of 95 %.

Results: The results are depicted in Table S18

*Table S18 Correlation of the % of residual metabolic activity of MDA-MB-231 cells after incubation with compounds **1**, **4**, and **5**, with the corresponding values of COX-1 and COX-2 inhibition*

	% Metabolic Activity (MDA-MB-231) vs. COX-1 (%)	% Metabolic Activity (MDA-MB-231) vs. COX-2 (%)
Pearson r	0,8453	-0,3682
R squared	0,7146	0,1356
P (two-tailed)	0,3588	0,7599
P value summary	ns	ns
Significance (alpha = 0.05)	No	No

# Current address: Sagl 26, A-6410 Telfs, Austria

References

(1) Cismesia, A. P.; Bell, M. R.; Tesler, L. F.; Alves, M.; Polfer, N. C. Infrared Ion Spectroscopy: An Analytical Tool for the Study of Metabolites. *Analyst* **2018**, *143* (7), 1615–1623. https://doi.org/10.1039/C8AN00087E.

(2) Corinti, D.; De Petris, A.; Coletti, C.; Re, N.; Chiavarino, B.; Crestoni, M. E.; Fornarini, S. Cisplatin Primary Complex with L-Histidine Target Revealed by IR Multiple Photon Dissociation (IRMPD) Spectroscopy. *ChemPhysChem* **2017**, *18* (3), 318–325. https://doi.org/https://doi.org/10.1002/cphc.201601172.

(3) Paciotti, R.; Corinti, D.; Maitre, P.; Coletti, C.; Re, N.; Chiavarino, B.; Crestoni, M. E.; Fornarini, S. From Preassociation to Chelation: A Survey of Cisplatin Interaction with Methionine at Molecular Level by IR Ion Spectroscopy and Computations. *J. Am. Soc. Mass Spectrom.* **2021**, *32* (8), 2206–2217. https://doi.org/10.1021/jasms.1c00152.

(4) Polfer, N. C. Infrared Multiple Photon Dissociation Spectroscopy of Trapped Ions. *Chem. Soc. Rev.* **2011**, *40* (5), 2211. https://doi.org/10.1039/c0cs00171f.

(5) Parneix, P.; Basire, M.; Calvo, F. Accurate Modeling of Infrared Multiple Photon Dissociation Spectra: The Dynamical Role of Anharmonicities. *J. Phys. Chem. A* **2013**, *117* (19), 3954–3959. https://doi.org/10.1021/jp402459f.

(6) Kadokawa, R.; Fujie, T.; Sharma, G.; Ishibashi, K.; Ninomiya, K.; Takahashi, K.; Hirata, E.; Kuroda, K. High Loading of Trimethylglycine Promotes Aqueous Solubility of Poorly Water-Soluble Cisplatin. *Sci. Rep.* **2021**, *11* (1), 1–6. https://doi.org/10.1038/s41598-021-89144-0.
